# Effectiveness of legally mandated non-custodial drug and alcohol treatment orders for improved health, well-being, global functioning and quality of life: a systematic review and meta-analysis

**DOI:** 10.1186/s40352-025-00354-4

**Published:** 2026-01-27

**Authors:** Pauline Campbell, Julie Cowie, Bridget Davis, Candida Fenton, Alex Todhunter-Brown, Hilda Bissozo Hernandez, Louise Hoyle, Hannah Carver, Catriona Connell, Joshua Dumbrell, Rosie Hill, Fiona Blacklaw, NIHR Evidence Synthesis Scotland InitiativE (NESSIE), Emma. F. France

**Affiliations:** 1 Glasgow Caledonian University, Glasgow, United Kingdom; 2 University of Edinburgh, Edinburgh, United Kingdom; 3 University of Stirling, Stirling, United Kingdom; 4 Patient and Public Involvement Member, Scotland, United Kingdom

**Keywords:** Adverse events, Anxiety, Criminal justice, Depression, Family members, Global functioning, Meta-analysis, Quality of life, Substance use, Systematic review, Treatment order

## Abstract

**Background:**

Adults in the criminal justice system are disproportionately more likely to use alcohol and drugs compared to the general population. Legally mandated alcohol and drug treatment orders have been proposed as an alternative to prison. However, little is known about how treatment orders affect the health and well-being of this population.

**Methods:**

A systematic review and meta-analysis. We searched 14 electronic databases (last searched November 2023) for studies comparing adults in legally mandated non-custodial drug and alcohol treatment orders to those receiving mandatory treatment orders or usual care. Global functioning, quality of life, drug or alcohol use measures, dependence severity, depression/anxiety outcomes, family member/significant other outcomes, and adverse events were selected based on a minimum core outcome set. We performed a meta-analysis using mean differences and risk ratios with 95% confidence intervals. We assessed the certainty of the evidence using GRADE. Equity-related factors were mapped to the PROGRESS-plus framework. People with lived experience provided input throughout the review process.

**Results:**

From 6917 records, 11 studies involving 4643 individuals (70% men; seven randomised controlled trials (RCTs)) met the eligibility criteria. All studies were conducted in high-income countries and involved drug and alcohol courts. The main outcomes of global functioning and quality of life were not reported. Poor reporting limited the meta-analysis. There were no differences between the groups receiving the intervention and those in the control group regarding number of positive drug screenings (MD -0.80, 95% CI -3.60 to 2.00, 10 participants, *p* = 0.58); depression (RR 0.93, 95% CI 0.78 to 1.10, 1533 participants, *p* = 0.38); or serious adverse events (RR 0.33, 95% CI 0.02 to 6.65, 10 participants, *p* = 0.47). We judged the evidence as very-low. The equity criteria most frequently reported were age, sex and race/ethnicity.

**Conclusions:**

The evidence is insufficient to draw judgements about the effectiveness of treatment orders for health and well-being. We found no evidence relating to global functioning, quality of life, anxiety, and outcomes specific to family members or significant others. High-quality RCTs are urgently needed. Future studies should involve people with lived experience in the design and conduct of new trials.

Study protocol registration.

The protocol for this study was registered on PROSPERO: CRD42023484923.

**Supplementary Information:**

The online version contains supplementary material available at 10.1186/s40352-025-00354-4.

## Background

Adults who use alcohol and drugs are over-represented in the criminal justice system (CJS) (Clark et al., 2017; Gallagher, 2014; Kinner & Young, 2018; Newbury-Birch et al., 2022; SAMHSA, 2021, 2023). Worldwide, nearly one in four newly incarcerated individuals will experience a serious alcohol use problem, and almost 40% will have a problem with drugs (Favril et al., 2024). The consequences of substance use problems are extensive, with more than 200 disease- and injury-related conditions described in the literature (Fazel et al., 2022; GBD 2016 Alcohol and Drug Use Collaborators, 2018; United Nations, 2022; World Health Organization, 2024). People involved in the CJS have a poorer health profile compared to the general population (Favril et al., 2024; GBD 2016 Alcohol and Drug Use Collaborators, 2018). Mortality rates are also higher in adults involved in the CJS compared with the general population (Borschmann et al., 2024; Favril et al., 2024; ONS, 2023; SAMHSA, 2023). The economic burden, loss of productivity and impact of drug- and alcohol-related crimes in our society are substantial (UK Government, 2020). The direct and indirect costs borne by individuals, families and wider society are incalculable (McLaughlin et al., 2024).

The global prison population is conservatively estimated to exceed 10 million individuals (Clark et al., 2017; McLeod et al., 2020). However, the turnover rate within prison populations may be at least three times higher than current estimates (Clark et al., 2017). It is widely acknowledged that prisons frequently lack the resources to adequately address the needs of justice-involved individuals who have drug- and alcohol-use problems (Kinner & Young, 2018; McLeod et al., 2020). Prison overcrowding occurs when the prison population exceeds its designed capacity, leading to significant negative consequences (MacDonald, 2018; Warner & Kramer, 2009). Overcrowding is associated with extended cell time, fewer opportunities to engage in developing skills necessary for successful reintegration, and compromised delivery of rehabilitation programmes (MacDonald, 2018; Penal Reform International, 2025). People in prisons often report that they have trouble getting medications like opioid agonist treatments, such as methadone (Komalasari et al., 2021; Marshall et al., 2023). They also face higher risks of overdose linked to short periods of incarceration (Borschmann et al., 2024; Bukten et al., 2017), as well as starting to use drugs again after being released (Borschmann et al., 2024; Keen et al., 2021). Custodial sentences have also been widely criticised for their failure to rehabilitate, as they do not sufficiently address key underlying contributors to offending, such as poor mental health, trauma or homelessness (Eaton & Mews, 2019; McLeod et al., 2020; Trebilcock, 2011; Wermink et al., 2023).

Legally mandated non-custodial drug and alcohol ‘treatment orders’ have emerged as an alternative to incarceration (Clark et al., 2017; Perkins et al., 2022). Treatment orders are initiatives in which individuals are mandated by CJS or CJS diversion programs to undertake, participate in, and complete healthcare treatment for drug or alcohol problems. These mandatory interventions are likely to comprise a mix of pharmacological and/or psychosocial treatments delivered across a range of healthcare contexts. Examples include:

- Alcohol abstinence monitoring requirements. These enable courts to mandate that an individual refrain from consuming alcohol for a specified duration and undergo regular testing and monitoring to verify compliance (United States Courts, 2025).
- Alcohol monitoring tags, which are also referred to as sobriety tags or alcohol abstinence tags (Bainbridge, 2019), or wearable alcohol biosensors, are electronic devices worn on the ankle or wrist (DiMartini et al., 2025). Regular sweat samples collection ensures a continuous record of alcohol consumption.
- Community payback orders which require individuals to adhere to a maximum of nine distinct requirements, which may include fulfilling unpaid labour, providing restitution, or being under the supervision of a social worker (Scottish Government, 2023).
- Drug treatment and testing orders are non-custodial penalties that empower the court (with consent of the justice-involved adult), to mandate the individual to undergo treatment, including compulsory testing, for drug use disorders (Scottish Government, 2023).
- Specialised courts, such as drug and alcohol courts, are led by judges who have expertise in these non-traditional court systems. These courts were formed to engage and supervise individuals with substance use problems, allowing them to receive the necessary treatment (Baughman et al., 2019; Green & Rempel, 2012; Lindenfeld et al., 2022).

Non-custodial judicial treatment orders originated in the United States with the establishment of the first drug court in Miami-Dade County, Florida, in 1989 (Hibbard, 2022) and were later implemented across other countries. However, research has focused almost solely on whether these interventions have reduced recidivism (Bright & Martire, 2013; Justice Analytical Services, 2023; Logan & Link, 2019; Trood et al., 2021; Werb et al., 2016; Zanis et al., 2003). The impact of treatment orders on the health and well-being of justice-involved individuals with drug and/or alcohol use problems remains uncertain. Addressing the health needs of justice-involved individuals offers the possibility to reduce offending and reoffending rates with considerable societal advantages and cost savings for healthcare, social services, law enforcement, and the CJS.

We have conducted two complementary systematic reviews: one bringing together quantitative evidence about the effects of mandatory treatment on the health, well-being, global functioning and quality of life of justice-involved people with substance use problems and a qualitative evidence synthesis. The goal of the qualitative evidence synthesis was to explore the perceived impacts on health and well-being of treatment orders and the perceived barriers and facilitators to implementation from the perspectives of justice-involved adults, their family members/significant others, and staff delivering/mandating the treatment. The qualitative review is reported elsewhere (France et al., 2025), and also includes the integration of findings of both reviews Health & Justice, 13 (1). 10.1186/s40352-025-00361-5.

In this paper, we present our findings from the quantitative evidence synthesis. Specifically, this paper sought to answer the following research question: Are drug and/or alcohol treatment orders more effective for improving health and well-being outcomes for adults mandated to participate in treatment as part of non-custodial sentence conditions compared with no mandatory treatment, or usual justice system processes (i.e., treatment as usual)?

## Methods

### Study design

We conducted a systematic review and meta-analysis (where there were suitably comparable studies) using established high-quality methods for Cochrane reviews of effectiveness (Higgins et al., 2023). We developed the protocol with input from topic experts (CC, HC, JD), patient and public contributors (RH, FB), and methodological experts (PC, BD, EF, ATB). The protocol was published in PROSPERO: CRD42023484923 (see https://www.crd.york.ac.uk/PROSPERO/view/CRD42023484923). We present our findings following the relevant reporting guidelines (Page et al., 2021) (see Additional file 1).

## Patient and public involvement (PPI)

Involving individuals with lived experience in the synthesis of evidence that is relevant to them enhances equity, accessibility, and the overall quality of the synthesis (Pollock et al., 2019). We had input from people with lived experience of treatment orders, as well as affected family members and significant others, alongside professional knowledge and experts throughout the entire review process. We use key checklists for PPI (Pollock et al., 2019; Staniszewska et al., 2017) to report details of involvement (see Additional file 2).

## Search strategy and selection criteria

### Eligibility criteria

Table 1 summarises the eligibility criteria. We searched the Core Outcome Measures in Effectiveness Trials (COMET) database (https://www.comet-initiative.org/) to identify a minimum core outcome set for use in this review. One core outcome set was identified that was directly relevant to this review (International Consortium for Health Outcomes Measurement (ICHOM), 2022). We selected the outcomes for this review based on the recommended minimum core outcome reported in this publication (International Consortium for Health Outcomes Measurement (ICHOM), 2022) and following an in-depth discussion with members of our professional knowledge expertise group (Table 1).

**Table 1:** Eligibility criteria

	**Inclusion**	**Exclusion**
Participants	• Adults aged 18 years and over who had received mandatory drug or alcohol treatment as part of a non-custodial sentence • Participants could have entered the intervention at any stage of the treatment order	• Participants who were juvenile offenders • Studies which focused on families
Intervention	• Any non-custodial intervention that included a mandatory treatment component within it. This included (but was not limited to): drug treatment and testing orders, community payback orders, alcohol tags, and specialist courts e.g., drug and alcohol courts	• Family Alcohol and Drug Court orders as they do meet the criteria for mandatory treatment • Studies which did not report any health and well-being outcomes
Comparator	No mandatory treatment order as treatment as usual (as described by authors)	• An active treatment delivered outwith a treatment order
Outcomes	Based on core outcome set (International Consortium for Health Outcomes Measurement (ICHOM), 2022): Primary outcomes: (1) Global functioning: substance-use specific (e.g. Substance Use Recovery Evaluator and generic measures (e.g. WHODAS 2.0, PROMIS-10, Global Assessment of Functioning) (2) Quality of life: substance-use specific (e.g. Addiction Severity Index) and generic QoL measures (e.g. SF-36, SF-12, EQ-5D, WHOQOL-BREF) Secondary outcomes: (1) Drug or alcohol use measures reported as: • self‐reported frequency and quantity drug or alcohol use (e.g., Addiction Severity Index composite scores, timeline follow back method, Alcohol Use Disorders Identification Test; or • biological alcohol and/or drug use (e.g. measured by testing urine, saliva or analysing hair for drugs, breathalyser for alcohol) (2) Severity of dependence (e.g. Leeds Dependence Questionnaire, Severity of Alcohol Dependence, Severity of Dependence Scale, Addiction Severity Index composite scores) (3) Depression and anxiety measured using, for example, the Hospital Anxiety and Depression Scale, Beck Depression Inventory (4) Family member/significant other outcomes measured using, for example, depression and anxiety (5) Adverse events/unintended consequences (examples may include accidental drug overdose, suicide)	• All other outcomes not listed in the inclusion criteria
Study design	A recent review of community sentencing (Justice Analytical Services, 2023) highlighted a paucity of studies using the most rigorous methodologies such as RCTs. Therefore we included quantitative evidence from a broader range of study designs following the Cochrane Effective Practice and Organisation of Care (Cochrane Effective Practice & Organisation of Care, 2017) guidance. This included evidence from RCTs, quasi-RCTs, non-randomised controlled trials, and controlled before-and-after studies	All other study designs were excluded i.e. systematic reviews and evidence syntheses, cohort studies, case–control studies, surveys/cross-sectional studies, case-series, commentaries, and opinion articles
Language	Studies published in English only	Studies published in languages other than English were excluded due to the additional resources and time required for translation
Date	No date restrictions were applied	Not applicable

### Searches

Searches were developed and run by an experienced Information Specialist (CF). We searched 12 electronic databases (MEDLINE, Embase, CINAHL, PsycINFO, Web of Science, LexisPSL, Westlaw UK, National Criminal Justice Reference Service, Applied Social Science Index and Abstracts, International Bibliography of Social Science, Policy Commons, Social Care Online) and clinical trial registers (World Health Organization International Clinical Trials Registry Platform and ClinicalTrials.gov) (date last searched, November 2023, see Additional File 3). We restricted searches by language, only including English-language publications. No date limitations were applied (Table 1).

In addition, we searched the reference lists of included studies and relevant systematic reviews for eligible studies and conducted forward citation searching for included studies (Haddaway et al., 2021).

## Data collection and analysis

### Selection of studies

Search results were combined and de-duplicated using Endnote (The Endnote Team, 2013) and imported to Covidence (Covidence, 2024). Two reviewers (PC, BD, EF, JC, ATB, LH) independently screened titles, abstracts, and full-text publications. We resolved disagreements through discussion with a third topic expert reviewer (CC, HC) when necessary.

## Data extraction and coding

Two review authors (PC, JC) independently extracted data from all studies using a pre‐developed data extraction form within Covidence (Covidence, 2024). We extracted and categorised data for the following items:

- study details: including author, year, aim, design, country, inclusion/exclusion criteria
- demographic characteristics: including age, sex, race and/or ethnicity and any other equity factors that stratify health opportunities and outcomes reported using the PROGRESS-plus framework (Cochrane Methods Equity, 2021)), alcohol and drug use, criminal history, post-traumatic stress disorder/trauma, adverse life experiences (if reported)
- clinical factors: including any reported co-morbid physical or mental health conditions coded using the ICD-11 (ICD-11, 2023), hospitalisations, frequency of health service contacts/use
- intervention characteristics: described using the TIDieR (template for intervention description and replication) framework (Hoffmann et al., 2014), programme theory underpinning the intervention and any details about the therapeutic relationship
- comparator characteristics: using the TIDieR (Template for Intervention Description and Replication) framework (Hoffmann et al., 2014)
- outcomes: as described in Table 1.
- study funding and conflict of interest.

## Assessment of methodological quality

### Quality of included studies

Risk of bias was assessed by two independent reviewers (PC, JC) using the risk of bias criteria recommended by the Cochrane Effective Practice and Organisation of Care group (Cochrane Effective Practice and Organisation of Care, 2017a). Studies were judged as being at high, low or unclear risk of bias for the following nine domains:

- random sequence generation
- allocation concealment
- baseline characteristics
- baseline outcome measurements
- knowledge of the allocated interventions adequately prevented during the study
- protection against contamination
- incomplete outcome data (attrition bias)
- selective outcome reporting (reporting bias)
- other risks of bias.

When the original report provided insufficient details, we sought data from the study authors. Disagreements were resolved through discussion, involving a third review author when necessary. We used the Robvis web app to create a risk of bias assessment visualisation (McGuinness & Higgins, 2021).

## Data synthesis and statistical analysis

The data was tabulated and summarised narratively, supported by evidence tables. Where suitable statistical summary data were available, we conducted a pairwise meta‐analysis for all primary and secondary outcomes listed (Table 1). We estimated pooled effect sizes (with 95% confidence intervals) using data from individual arms of included trials. We calculated risk ratios for binary outcomes, and mean differences for continuous outcomes (or standardised mean differences if different measures of the same outcomes were used in different trials). Randomised and non‐randomised studies were meta-analysed separately (Higgins et al., 2023). Further details of our analysis plan are reported in the protocol (https://www.crd.york.ac.uk/PROSPERO/view/CRD42023484923.) and any changes from the protocol are documented in Additional file 4.

## Certainty assessment

We used the Grading of Recommendation, Assessment, Development and Evaluation (GRADE) system to highlight the confidence in quantitative evidence findings. The evidence was assessed across five domains: methodological limitations (Guyatt, Oxman, Vist, et al., 2011); imprecision (Schunemann et al., 2022; Zeng et al., 2022); inconsistency of results (Guyatt, Oxman, Kunz, Woodcock, Brozek, Helfand, Alonso-Coello, Glasziou, et al., 2011); indirectness of evidence (Guyatt, Oxman, Kunz, Woodcock, Brozek, Helfand, Alonso-Coello, Falck-Ytter, et al., 2011); and publication bias (Guyatt, Oxman, Montori, et al., 2011). We used these assessments to arrive at an overall judgement (high, moderate, low or very-low) regarding the quality of the evidence for each outcome. We constructed a summary of findings table using GRADE to provide a summary of the key findings alongside a summary of the volume of the data, effect size and overall evidence quality.

## Results

### Results of the search

**Fig. 1:**
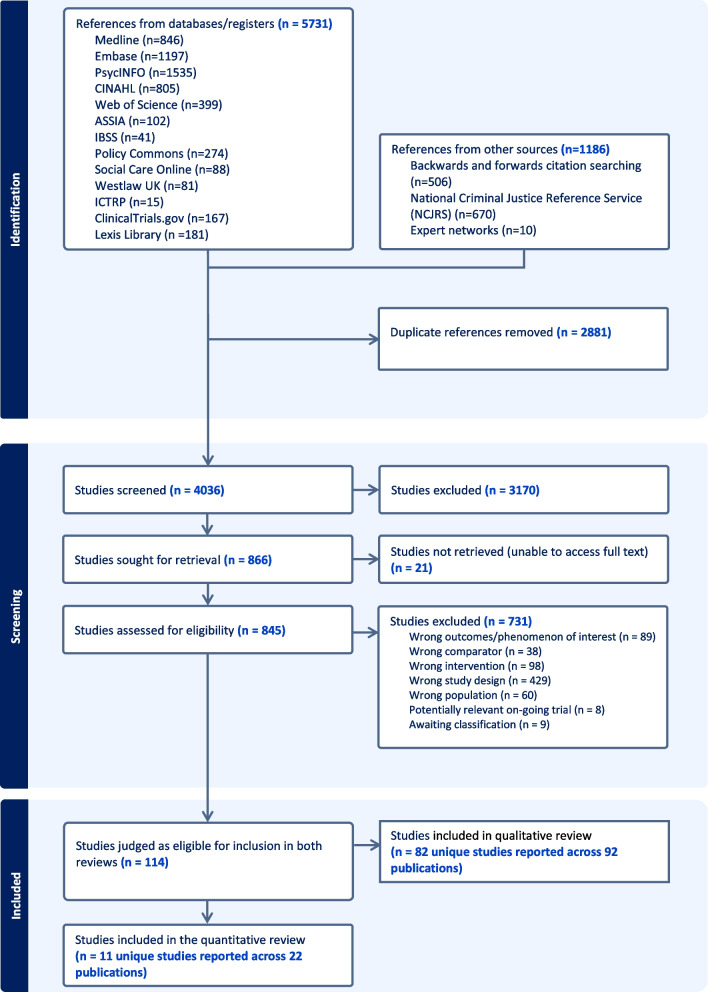
PRISMA flowchart

Our searches identified 6917 records (Fig. 1). After title and abstract screening, 845 records underwent full-text screening, of which 731 were excluded (Additional file 5). Twenty-two publications met the eligibility criteria, representing 11 unique studies (Table 2).

**Table 2:** Key study characteristics

First author (year) (Main study reference)	Study design (Sample size, n)	Country	Participants				Intervention*	Comparator
			***Age (years)***	***Sex (M/F)***	***Ethnicity/*** ***Race***	***Education***		
**Deschenes 1995** (Deschenes et al., 1995)	RCT (*n* = 630)	USA	**IG:** 29.2 **CG:** 30.2	491/139	**IG:** African American: 21.6%; Hispanic: 27.8%; White: 48.3% **CG:** African American: 18.7%; Hispanic: 23.3%; White: 55.7%	**IG:** No high school diploma: 56.3% **CG:** No high school diploma: 47.7%	Drug Court	Routine probation
**Desland 1992** (Desland & Batey, 1992)	NRT (*n* = 92)	Australia	**IG**: Male age (SD): 22.9 (6.5); Female age (SD): 21.2 (4.7) **CG**: Male age (SD): 26.6 (4.2); Female age (SD): 24.8 (4.4)	51/41	NR	**IG**: Education (range): males 8–10 years; females 7–12 years **CG:** Education (range): males 9–16 years; females 10–16 years	Drug Court	Self-referred
**Festinger 2016** (Festinger et al., 2016)	RCT (*n* = 200)	USA	**IG:** Mean age (SD): 25.37 (7.69) **CG:** Mean age (SD): 23.77 (6.61)	163/37	**IG:** African-American: 66%; Caucasian: 12%; NR/missing: 22% **CG:** African-American: 62%; Caucasian: 11%	NR	Computerised HIV prevention intervention delivered in drug court	Attention control
**Gottfredson 2002** (Gottfredson & Exum, 2002) (Gottfredson et al., 2005, 2006, 2007; Kearley, 2018; Kearley et al., 2019)	RCT (*n* = 235)	USA	**IG:** Mean age (SD): 34.8 (7.5) **CG:** Mean age (SD): 34.7 (7.9)	174/61	**IG:** African-American: 89.2% **CG:** African-American: 89.6%	**IG:** 47% had at least a high school education **CG:** NR	Drug Court (Baltimore Drug Treatment Court)	Treatment as usual
**Green 2012** (M. Green & M. Rempel, 2012)	NRT (*n* = 1474)	USA	**IG:** 33.69 **CG:** 34.28	1027/447	**IG:** White: 55%, Black/African American: 32%, Hispanic/Latino: 6%, Other: 7% **CG:** White: 54%, Black/African American: 25%, Hispanic/Latino: 5%, Other: 6%	**IG:** High school degree/GD or higher: 59% **CG:** High school degree/GD or higher: 60%	Drug Court	Comparison
**Harrell 1998** (Harrell et al., 1998)	RCT (*n* = 691)	USA	Sanctions docket (median age): 33 Treatment docket (median age): 29.6 Standard docket (median age): 31.2	589/102	Sanctions docket (African-American): 96%, Treatment docket (African-American): 99%, Standard docket (African-American): 96%	NR	Drug Court: 2 groups (i) Sanctions docket and (ii) Treatment docket	Standard docket
**Harrell 2001** (Harrell et al., 2001)	NRT (*n* = 397)	USA	**IG:** 35.3 **CG:** 36	0/397	**IG:** African-American:70% **CG:** African-American: 66.7%	NR	Drug Court i.e. Brooklyn Treatment Court	Comparison
Jones, 2013 (Jones, 2013)	RCT (*n* = 136)	Australia	**IG** (mean age):32.2 **CG** (mean age):32.5	114/22	**IG:** Indigenous: 13.6% **CG:** Indigenous: 8.6%	NR	Intensive Judicial Supervision delivered in Drug Court	Supervision as usual
**MacDonald 2007** (MacDonald et al., 2007)	RCT (*n* = 236)	USA	**IG:** 36.6 **CG:** 34.2	217/19	**IG:** Hispanic: 83.7% **CG:** Hispanic: 87.3%	**IG:** Education (years of school completed): 10.9 **CG:** Education (years of school completed): 10.8	DUI Court	Mandatory minimums
**NCT02978417** (NCT0, 2978417, 2016)	RCT (*n* = 10)	USA	**IG:** 36.2 (9.4) **CG:** 37.4 (13.4)	7/3	**IG:** Black or African-American: 20%; White: 80% **CG:** Black or African-American: 20%; White: 80%	NR	Drug Court (Vivitrol)	Treatment as usual
**Rodriguez-Monguio 2021** (Rodriguez-Monguio et al., 2021)	NRT (*n* = 542)	USA	**IG** (mean age at court intake, SD): 31.5 (8.54) **CG** (mean age at court intake, SD): 35.99 (11.43)	417/125	**IG:** White: 82.3%, African American: 6.6%, Hispanic: 9.2%, Other: 1.5%, Missing: 0.4% CG: White: 83.4%, African American: 5.5%, Hispanic: 10.3%, Other: 0.7%, Missing: 0%	NR	Drug Courts	Traditional Courts

*Key*: *CG* control group, *DUI* driving under the influence, *F* female, *IG* intervention group, *NR* not reported, *NRT* non-randomised trial, *HIV* Human Immunodeficiency Virus, *M* male, *RCT* randomised controlled trial

Sanctions refer penalties for failing drug tests, Treatment docket refers to sequential treatment programs offered, standard docket refers to a standard program of drug testing, judicial monitoring and encouragement to seek community-based treatment programs

^*^ Interventions are profiled using the TIDieR framework in Additional File 9

## Key characteristics of included studies

The 11 included studies were all conducted in high-income economies (World Bank Group, 2022). Nine of the studies were conducted in the US and two studies in Australia (see Table 2; Additional file 6). Six studies used a parallel-group RCT design. One study used a multi-arm (3-arm) RCT (Table 2); we present data from this study as two randomised paired comparisons—Harrell (1998) (i): sanctions docket vs standard docket and (ii): treatment docket vs standard docket. The remaining four studies employed a non-randomised design (Table 2).

## Participant characteristics

The data within the included studies represent 4643 participants, of which 2536 received a treatment order (Table 2). Age, sex and race and/or ethnicity were reported in all studies. The average age of participants ranged across studies from 21.7 years to 37.4 years, and the majority of participants were men (*n* = 3250/4643; 70%). One study exclusively presented data from women (Harrell et al., 2001). Most studies included adults from more than one race and/or ethnic background. Over half of our studies (*n* = 6) reported that 38.2–85.1% of participants had sought treatment for substance use before they entered the drug court. Additional file 7 contains further details on alcohol and drug use, as well as criminal history. Additional file 8 present equity data mapped to the PROGRESS-plus framework.

## Interventions

All studies involved a drug court system with varying degrees of mandated alcohol and drug treatments or programmes (see Additional file 9). Participants received a combination of court supervision and treatment programmes. Participants received a combination of court supervision and treatment programmes. Specialised alcohol and drug treatment programmes were multi-component and included a mixture of education classes, skills training, community-based treatments (e.g., 12-step meetings), outpatient treatments (e.g., individual or group counselling), and inpatient treatments, including pharmacotherapy and, in some cases, detoxification treatment.

Court supervision included monitoring of behavioural contracts which included a variety of rewards or sanctions, probation officer/case management contacts, and random urine tests. Intervention programmes varied across the studies depending upon the geographical area or type of court the participant was enrolled in.

Intervention providers were described in eight studies, with drug court judges (Gottfredson & Exum, 2002; Harrell et al., 1998, 2001; Jones, 2013) and clinical staff/other treatment providers (Harrell et al., 2001; Jones, 2013; NCT0, 2978417, 2016; Rodriguez-Monguio et al., 2021) delivering the intervention in four studies. A variety of other personnel were also involved including: case managers/case management services (Harrell et al., 1998; Rodriguez-Monguio et al., 2021), community organisations (Gottfredson & Exum, 2002), government employees working in employment or social security departments (Harrell et al., 1998), parole and probation officers/employees (Deschenes et al., 1995; Gottfredson & Exum, 2002), private agencies (Deschenes et al., 1995; Desland & Batey, 1992) and prosecutors/defence attorneys (Harrell et al., 2001). Intervention provider qualifications or training to deliver the intervention were not reported in any study.

In most studies, it was difficult to determine how the intervention was delivered, how many treatment sessions were delivered, and the duration of intervention (at the level of the drug court and for individual treatment components) because of poor reporting or the data not being captured. The intervention was tailored for individual participants in most studies, usually based on the outcome of a urine test.

## Comparators

The interventions that participants in the control groups received were poorly documented and were reported as usual care in most studies (*n* = 10) and attention control in one study (Festinger et al., 2016). However, very few comparison groups were solely usual care or no treatment (Additional file 9). For instance, usual care included routine probation (Deschenes et al., 1995), treatment/supervision delivered in a drug court as usual (Gottfredson & Exum, 2002; M. Green & M. Rempel, 2012; Harrell et al., 1998; Harrell et al., 2001; Jones, 2013; Rodriguez-Monguio et al., 2021); mandatory minimums (MacDonald et al., 2007); and self-referral to drug court (Desland & Batey, 1992) (see Additional file 9).

## Outcomes measures

Our primary outcomes of interest (global functioning and quality of life) were not reported by any of the studies. Nine studies reported our secondary outcome measures of interest (see Additional File 10). No studies reported family members'or significant others’ outcomes. Additional file 11 summarises various additional outcomes that fell outside the scope of this review.

Most of the studies report outcomes from six months to 24 months. One RCT (Gottfredson & Exum, 2002) was also linked to a series of studies that reported data at three years (Gottfredson et al., 2005, 2006, 2007) and 15 years follow-up (Kearley, 2018; Kearley et al., 2019). We have not included this data in our meta-analyses because it was outside the scope of our review.

## Sources of funding and potential conflicts of interest

Most studies disclosed funding sources (*n* = 9). Two studies declared no conflicts of interest, while the remaining studies did not report their conflicts of interest (Additional file 6).

## Risk of bias in included studies

**Fig. 2:**
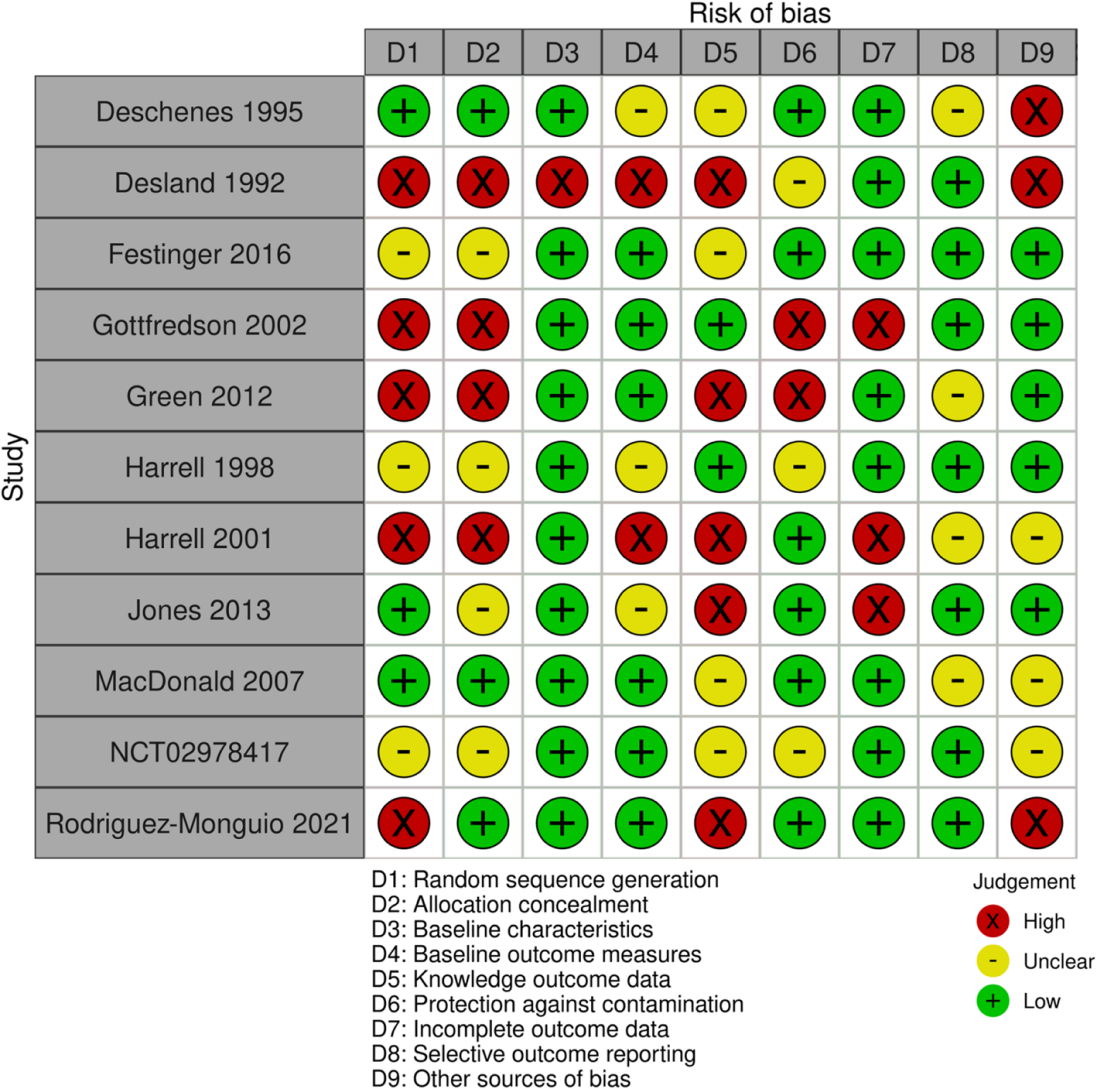
ROB analysis

The risk of bias for included studies is summarised in Fig. 2, and the consensus judgements underpinning the risk of bias assessments (based on two independent reviewers) for each study are reported in Additional file 12.

## Synthesis of results

In the following section, we present the findings reported across our studies. Table 3 provides a summary of findings, detailing the certainty of evidence using GRADE for each of our predefined outcomes. Forest plots are presented in Additional file 13. A summary table of results, as reported by the study authors, for studies relevant to a specific outcome that lacked suitable data for meta-analysis is provided in Additional file 10.

## Primary outcomes

*Global functioning or QoL outcomes*: *substance use specific outcomes.*

Not reported in any study.

*Global functioning or QoL outcomes*: *generic outcomes.*

Not reported in any study.

## Other secondary outcomes

### Drug or alcohol use measures: self-reported frequency of drug use

Two RCTs investigated the consumption of alcohol and/or drug use using self-reported measurements (Harrell et al., 1998; MacDonald et al., 2007). Neither of these studies had statistical data suitable for inclusion within the review meta-analyses.

Harrell (1998) assessed drug use after the intervention period in individuals assigned to a sanctions docket or a standard docket (691 participants). The measurement was based on self-reported data regarding the quantity and variety of drugs used in the twelve months following the sentence. Study authors reported no notable differences between the individuals eligible for either type of docket, even after accounting for other variables in their statistical model. Furthermore, they indicate that there were no notable reductions in the use of more potent drugs among those assigned to the sanctions docket during the year after their sentencing when compared to participants enrolled in the standard docket.

MacDonald (2007) surveyed 236 participants about the number of days drinking five or more drinks at 24 months. They found that being assigned to the DUI court did not reduce the number of days (in the prior 30 days) that a participant drank more than five alcoholic beverages (MacDonald et al., 2007).

Two non-randomised studies also reported data for drug use and serious drug use in the past 30 days and past six months (Harrell et al., 2001), methadone use (Desland & Batey, 1992) and number of days experiencing problems with alcohol or drugs (Harrell et al., 2001). (Additional file 10).

### Drug or alcohol use measures: biological measures of alcohol and/or drug use

The number of positive tests for alcohol/drug use was reported in three RCTs (Deschenes et al., 1995; Jones, 2013; NCT0, 2978417, 2016). However, two studies lacked appropriate statistical data for inclusion in the meta-analyses (Deschenes et al., 1995; Jones, 2013).

The number of positive drug screens was reported in one RCT (NCT0, 2978417, 2016). There were no statistical differences between the groups after 12 months in terms of the number of positive drug tests (MD −0.80, 95% CI −3.60 to 2.00, 10 participants, *p* = 0.58). We judged to be of very-low certainty. We downgraded the evidence once for risk of bias and twice for imprecision (Additional File 13).

Urinalysis data (Desland & Batey, 1992) and relapse based on the number of positive drug tests (Rodriguez-Monguio et al., 2021) were also reported in two non-randomised studies (see Additional file 10).

### Severity of dependence

One RCT (236 participants) reported dependence severity (MacDonald et al., 2007). Suitable statistical data permitting inclusion within the review meta-analyses were unavailable. MacDonald (2007) reported no significant difference in rates of binge drinking or alcohol issues in participants who received the drug court intervention compared to the control group at 24 months (Additional file 10).

### Depression and anxiety measures

The frequency of depression symptoms was measured in one non-randomised study (M. Green & M. Rempel, 2012). There was no significant difference in the number of drug court participants who reported feeling depressed compared to participants in the control group at six months (RR 0.93, 95% CI 0.78 to 1.10, 1533 participants, *p* = 0.38) or at 18 months (RR 0.93, 95% CI 0.78 to 1.10, 1474 participants, *p* = 0.40). We judged the evidence to be of very-low certainty. We downgraded the evidence twice for serious risk of bias and once for indirectness due to the different treatments delivered to individuals (Additional file 13).

No study reported any anxiety outcomes.

### Family member/significant other outcomes

No study reported outcomes related to family members or significant others.

### Adverse events/unintended consequences

All-cause mortality, serious adverse events, and other events (e.g., injury, poisoning and procedural complications) were reported at six months in one RCT (*n* = 10 participants) (NCT0, 2978417, 2016). No deaths were reported in either the drug court group or the control group (NCT0, 2978417, 2016). Participants in the drug court intervention had fewer serious adverse events compared with participants in the control group, but the difference was not significant (RR 0.33, 95% CI 0.02 to 6.65, 10 participants, *p* = 0.47). We judged the evidence to be of very-low certainty. We downgraded the evidence once for risk of bias and twice for imprecision.

We did not combine data for other events within the meta-analysis (see Additional file 13).

## Discussion

### Summary of findings

To the best of our knowledge, this is the first systematic review and meta-analysis to investigate the effectiveness of alcohol and drug treatment orders in improving health and well-being outcomes for adults legally required to participate in treatment as part of a non-custodial sentence. Our review found 11 studies (7 RCTs; 4643 justice-involved adults: 70% male with an average age range of 21 to 37 years); all treatment order interventions were provided in specialist court settings, such as drug and alcohol courts, and studies were conducted in high-income countries (Australia and the US). We identified no other treatment order interventions.

We found no high-or-moderate-certainty evidence about the effectiveness of legally mandated non-custodial drug and alcohol treatment orders on health and well-being outcomes. Our meta-analyses found little evidence (with very-low certainty) for the following outcomes: the number of positive drug tests, the frequency of depression symptoms, and serious adverse events. In most cases, we were unable to conduct a meta-analysis due to a lack of studies, inadequately reported outcome data, and methodological concerns about the study's design and quality of reporting. In addition, few comparison groups were genuinely receiving usual care or no treatment. Consequently, there is insufficient evidence to support any conclusions about the effectiveness of treatment orders in the drug court setting. We found an absence of evidence relating to the following outcomes: global functioning, quality of life, anxiety and outcomes specifically concerning family members or significant others. Our finding represents a major gap in the evidence base.

Substance use (alcohol and drug) was identified as a significant issue among participants, with 38.2% to 85.1% of individuals in six studies reporting seeking treatment before entering drug court. However, most of the included studies focused on metrics associated with criminal behaviour and recidivism, prioritising the reduction of alcohol- and drug-related crime over treatment or rehabilitation outcomes. The studies included in the review seldom looked at the results mentioned in a new core-outcome set (Black et al., 2024) and usually did not explain the complicated details of the treatments or additional support provided to participants in both the intervention and control groups. This constrains the conclusions derived from the existing evidence.

A major strength of this review is that we profiled equity and wider determinants of health reported in the included studies, using the PROGRESS-plus framework. Age, sex and race/ethnicity were the most frequently reported equity-related factors. One of our studies involved only women (Harrell et al., 2001). No studies reported sexual orientation or religious identity. Other demographic characteristics, including case variables and equity factors as recommended in the recent consensus set of outcomes, were poorly reported (Black et al., 2024). The paucity of data in this area and poor reporting meant that we were unable to examine equity-related factors in more detail or conduct planned subgroup analysis. Improved reporting across multiple dimensions of inequality and social stratifiers is necessary to allow in-depth data profiling and provide a richer context for understanding which intervention works best and for whom.

## PPI reflection

There is a growing recognition that involving individuals with lived experience in synthesising evidence that is meaningful to them improves equity and accessibility and the overall quality of the synthesis (Pollock et al., 2019). Engagement can identify evidence gaps, reduce obstacles to using evidence, improve how results are shared and used, and help create research recommendations (Brett et al., 2014; Cottrell et al., 2014).

Our review included the involvement of people with lived and family member experience, as well as those with professional knowledge and expertise, during the review process (Additional file 2). The combined input of professionals with expertise and knowledge of the topic area and people with lived/family member experiences proved extremely valuable. The professional knowledge and expertise were essential in conceptualising the review, writing and finalising the protocol, study selection, and analysis/interpretation of findings.

The participation of members of our lived experience group was a critical part of the review. They generously contributed their knowledge and insights throughout the review process. They were key in helping us understand the complexities of treatment orders and the multiple challenges involved in accessing services. They provided their perspectives on the review content, emphasising the value of thinking about equity factors and outcome measures, and helped with writing the final synthesis (Additional file 2). The involvement of people with lived experience has helped shape this research by identifying those gaps in the research and influencing discussion around recommendations for future research (see research implications below).

The key findings from this quantitative review were presented to the group in July 2024 (Additional file 2). Responses to the findings were:

*“I just feel it's quite, quite sad that things don't seem to have moved on from these studies, and we're still in this mess at the moment”*

and.

*“So there's massive big gaps in what's been said is actually happening and what is actually happening….. You know, there's nothing. No full picture”*

## Strengths and limitations

We used the highest methodological standards while conducting this review, adhering to established guidance for methodological conduct and reporting (Cochrane Effective Practice and Organisation of Care, 2017a; Guyatt, Oxman, Schunemann, et al., 2011, 2011b, 2011c; Higgins et al., 2023; Hoffmann et al., 2014; Page et al., 2021). We published a protocol with predefined criteria and outcomes linked to a core outcome set. We employed a comprehensive search strategy developed by an information specialist and conducted rigorous searching in 14 databases covering biomedical, social policy and legal subject areas to ensure all relevant publications were retrieved. Our review benefitted from the participation of people with lived experience, topic experts, and methodological experts. Collaboration with our stakeholders started early in the review process and they provided essential support in identifying potential third-sector organisations and judicial points of contact that could enhance our understanding of the review topic.

However, there are several limitations to this review. These principally relate to the quality of the research evidence available and poor reporting, which we have already highlighted. We also noted multiple challenges when screening the studies to determine whether they met our predefined selection criteria because of the limited description of the study design and data collection methods. This means we may have inadvertently missed some relevant studies. Our review could also be criticised for only synthesising studies, which included trials that compared one treatment order with no intervention, usual care, or no active control. Limiting the study's comparators limits the generalisability of the findings.

We selected the outcomes for this review based on an earlier core outcome set publication (International Consortium for Health Outcomes Measurement (ICHOM), 2022) alongside input from our lived experience group and topic experts. Since then, the consensus set of recommended outcomes has been published in full (Black et al., 2024). While the primary outcomes (global functioning and quality of life) have not changed in the updated version, a number of secondary outcomes have been added, and any update of this review should consider these changes when developing their protocol.

## Research implications

Future research is necessary to examine the impact of various treatment orders on health outcomes. Such investigations could include studies evaluating other models of treatment order (e.g., community payback orders, drug treatment and testing orders or wearable alcohol biosensors). Conducting high-quality research is understandably difficult, and we appreciate the practical, legal, and ethical methodological challenges that accompany work in this field. However, our review identified seven RCTs conducted under these challenging circumstances, offering some evidence of feasibility. Future studies should adhere to pre-defined protocols, publish them on relevant clinical trial registries, and ensure the publication of all results. Studies should always document the intervention's duration, the amount and frequency of any interventions delivered as part of the treatment order, and follow-up time points.

Future studies should also carefully consider what usual care is and document whether any co-interventions are being delivered. The use of comprehensive reporting templates such as TIDieR (Hoffmann et al., 2014) and the case variables reported in the recent minimum core outcome set (Black et al., 2024) could be a useful addition to work in this field.

The collection and reporting of equity-related data, such as age, sex, ethnicity, and socioeconomic status, is crucial in future research studies. This data further clarifies the social and structural determinants of health and informs the development of targeted interventions (McLeod et al., 2020).

Finally, it was not clear from any of the studies whether any justice-involved adults and/or their family/significant others were involved in the planning, design or conduct of the study. We believe this is a significant oversight, as engaging families and/or significant others within research in this field could potentially support the development of more relevant, acceptable, effective and meaningful interventions. People with lived experience should be involved in reaching consensus on the most important health outcomes and measurement tools for future research. Although the recently published core outcome set included people with lived experience, we believe it would be beneficial to include family members/significant others in this process and to consider the local context and geographical location in which studies are being conducted.

## Reflexivity

Adopting and transparently reporting a reflexive approach is good practice in research (Olmos-Vega et al., 2022). Additional file 14 reports our team’s positionality and reflexivity statement.

## Conclusions

In summary, we conducted a comprehensive systematic review and meta-analysis of evidence relating to the effect of alcohol and drug treatment orders on health and well-being outcomes for adults legally mandated to participate in treatment as part of a non-custodial sentence. We identified 11 studies that compared the provision of a treatment order mandated by a judge within a specialised drug court with normal justice system processes. The main finding from our review is that the current evidence is insufficient to support any generalised conclusions about the effectiveness of drug court interventions for health and well-being outcomes. We found an absence of evidence relating to the following outcomes: global functioning, quality of life, anxiety and those specifically concerning family members or significant others. This represents a major gap in the evidence base. The included studies rarely measured outcomes deemed of highest priority based on a recent core-outcome set, failed to report the treatments that control participants received, and inadequately reported equity-related factors and outcome data. This constrains the conclusions drawn from the existing evidence.

We urgently need future research, ideally in the form of high-quality RCTs, to examine the impact of treatment orders on health and wellbeing outcomes. Studies should address topics and outcomes identified to be of the highest priority and be planned and conducted following the highest methodological and reporting standards, with the involvement of people with lived experience. Future studies should also consider and report social determinants to allow a deeper understanding as to why a treatment order is successful or not for different populations in the CJS.

## Supplementary Information

Additional file 1. PRISMA checklist. Reporting guideline checklist of reported items

Additional file 2. Details of involvement using the ACTIVE and GRIPP2 reporting checklists. Details of PPI following reporting guidelines

Additional file 3. Search strategies. The terms used to search the 14 electronic databases

Additional file 4. Deviations from protocol. Description of changes from the protocol

Additional file 5. Table of excluded studies. The studies which did not meet review inclusion criteria with exclusion reasons

Additional file 6. Summary of included studies methods and selection criteria. The methods and selection criteria used within the included studies

Additional file 7. Participant characteristics. Summary of participants substance use, experience with criminal justice system and clinical factors

Additional file 8. PROGRESS-plus framework. A summary of participant characteristics mapped to the PROGRESS-plus framework

Additional file 9. Interventions profiled using the TIDieR framework. Interventions descriptions delivered mapped to the TIDieR reporting framework

Additional file 10. Relevant outcome measures and data relevant to the review. Summary of outcome measures that were relevant to the review and results as reported by study authors

Additional file 11. Outcome measures reported in the included studies that were not relevant to this review. Outcome measures/tools that were used in included studies but that did not meet the pre-defined outcomes of interest for this review

Additional file 12. ROB judgements. Consensus decisions for risk of bias judgement across nine EPOC domains

Additional file 13. Forest plots. Graphical data related to the forest plots conducted meta-analyses conducted

Additional file 14. Reflexivity. Details of the authors’ backgrounds, expertise and assumptions relevant to the review

## Data Availability

No datasets were generated or analysed during the current study.
