# Supplementary material for: Effectiveness of legally mandated non-custodial drug and alcohol treatment orders for improved health, well-being, global functioning and quality of life: a systematic review and meta-analysis
Source: Health Justice. 2026 Jan 27;14:11. doi: 10.1186/s40352-025-00354-4 (PMC12958499; doi:10.1186/s40352-025-00354-4)
Supplement: Supplementary file 2 — Additional file 2. Details of involvement using the ACTIVE and GRIPP2 reporting checklists. Details of PPI following reporting guidelines [file 40352_2025_354_MOESM2_ESM.pdf]

Additional file 2. ACTIVE and GRIPP2 Reporting checklists

Stakeholder/Patient and Public Involvement in two complementary reviews

This document reports patient and public involvement (PPI) and the stakeholder involvement by reporting:

1. GRIPP2 short form items (Table A)
2. ACTIVE framework descriptions of methods of involvement (Table B)
3. ACTIVE framework summary (Table C)

**Table A: GRIPP2 short-form for stakeholder involvement**		
**GRIPP2 short form**		
**Section and topic**	**Item**	**Report from this project**
1. Aim	Report the aim of PPI in the study	The aim of stakeholder/PPI involvement was to advise relating to aspects of the review including writing the protocol (stakeholders only), search strategy, selection of studies for inclusion, to provide insight into review data extraction and analysis/interpretation of findings, review of final publication draft.
2. Methods	Provide a clear description of the methods used for PPI in the study	We used two key modes of stakeholder/PPI engagement:<br>1. Stakeholders (n= 5) who had professional knowledge and experience of the topic area.<br>2. People and family members with lived experience of the topic area (n=5).<br>NB: Our anonymised co-applicant member participated in both groups.<br><br>See ACTIVE framework for detailed description of methods.
3. Study results	Outcomes—Report the results of PPI in the study, including both positive<br><br>and negative outcomes	See ACTIVE framework for detailed description of the outcomes of meetings.
4. Discussion and conclusions	Outcomes—Comment on the extent to which PPI influenced	The response from both stakeholders and PPI members was enthusiastic and extremely helpful throughout the review.

	the study overall. Describe positive and negative effects.	We ensured that enough time was given for people to respond to commenting on documents (e.g. protocol and search strategy). Stakeholders' professional knowledge and expertise was essential in conceptualising the reviews, writing and finalising the protocol, study selection and sampling decisions, analysis/interpretation of findings and final publication (in progress). However, despite concerted efforts we were unable to secure involvement from related charitable organisations and were declined involvement from judicial personnel.<br><br>Our group of 5 people with lived and family member experience joined the review at the sampling stage and were helpful in determining the positive and negative aspects of treatment orders and the types of people they viewed as being important to include within the review.
5. Reflections/critical perspective	Comment critically on the study, reflecting on the things that went well<br><br>and those that did not, so others can learn from this experience	The topic of substance use problems was potentially sensitive. The combined input of professionals with expertise and knowledge of the topic area and people with lived/family member experiences proved extremely valuable.<br><br>However, it was disappointing that we were unable to secure expert judicial input which would have been very helpful, especially in explaining and understanding some judicial processes. PPI/stakeholder input had a positive influence on the review results.

**Table B: ACTIVE framework constructs for stakeholder involvement in two complementary reviews**	
ACTIVE framework construct	Description
Who is involved?	**Stakeholder group**: A closed group of 5 people, present from the outset of the review that included representatives as follows: 3 scientific /

	academic members involved in substance use research; 1 PhD student involved in substance use research; 1 PPI co-applicant.<br><br>The group met online with members of the review team on 17th October 2023 and 23rd February 2024. Although, due to work commitments, apart from one person, they were unable to join the PPI advisory group meetings, the stakeholder group were consistently available for consultation and provided prompt advice by email relating to all aspects of the review.<br><br>**PPI Advisory Group:** The group comprised 3 family members of people with substance use problems who had been through a treatment order, 2 people with lived experience of substance use and treatment orders, and 1 PPI Co-applicant. The group met online with members of the review team on 24th April 2024, 26th June 2024 and 16th July 2024.<br><br>We plan to hold one further meeting to discuss dissemination following completion of the reviews.
How were people recruited?	**Stakeholder Group**: through original conception of the topic area with the application for this review.<br><br>**PPI Advisory Group:** Recruited in March 2023 and were invited through networking via the review stakeholder group's known contacts.
What happened?<br><br>When did it happen (stage & level)?	**Stakeholder Group**<br><br>Contributed to early-stage discussions involving the scope of the review, writing of the review protocol, qualitative study sampling framework wording/description/coding of the main themes from the review results.<br><br>They reviewed and gave written comments on draft versions of the protocol and final publication.<br><br>**Meeting 1 (17th October 2023):**<br><br>- Agreed group input into the review protocol including search strategy (deadline for submission of protocol 31.10.24).<br><br>- Discussed the PPI role of people with lived experience of substance use, and best ways of communicating and working together.<br><br>- Discussed contacts to invite potential stakeholders to advise and contribute from judiciary and 3rd sector organisations.<br><br>**Meeting 2 (23rd February 2024):**<br><br>- Update on review progress<br><br>- Discussed potential publications for inclusion<br><br>- Potential PPI contacts who are people with lived experience and family members.<br><br>- No progress with potential judicial or third sector members.

	- Discussed data extraction.<br><br>**NB: There were no further meetings with the stakeholder group, but contact was maintained by email.**<br><br>**<u>PPI Advisory Group meeting 24th April 2024</u>**<br><br>The group was given an overview of the review and an update on the eligible studies, and topic areas. They provided their perspectives on the review content including the sampling framework and inclusion of studies.<br><br>**- Provided perspectives on positive aspects of Treatment Orders (TO)**<br><br>• Regular drug testing and group support within a TO can help<br>• Some conditions of TOs are helpful (e.g. night-time curfew, although drugs can still be accessed during the day)<br>• Being able to return to work is really important<br>• Gaining a sense of responsibility is key to success of TOs<br>• Community Psychiatric Nurse and social care can be helpful<br>**- and aspects of treatment orders that do not work well:**<br><br>• CPN & social care support is often unreliable or unavailable<br>• Poor communication between agencies<br>• Lack of help with mental health problems (sometimes leading to risk of suicide)<br>• General practitioners lack knowledge<br>• Lack of specialised staff<br>• Lack of available, specialised rehabilitation centres<br>• Burden of care is on families and voluntary services<br>• The root of addiction is not addressed<br>• The danger of relapse is always present<br>The group also discussed **types of people** that they consider important to the review including people with both mental AND addiction problems and people from ethnic minorities.<br><br>**<u>PPI Advisory Group meeting 26th June 2024</u>**<br><br>The group were given an update on the review, and we shared some initial findings.<br><br>• There are no studies that include family members<br>• We have some data about relationships (including relationships with family, judges, and court staff)<br>• We have some data on housing and employment<br>• Six studies focus on women, some with children<br>• There is some data on mental health and trauma<br>• Other data included ethnic minorities that included USA: African Americans, Hispanic (Latinx) people, Native Australians<br>• Medication Assisted Treatment e.g. methadone

	**Summary of PPI Group feedback to the above**<br><br>• The family are still largely ignored/avoided and rely on local community groups set up by family members. More research should be carried out about the needs of the family<br>• Mental health issues are nearly always at the root of substance use problems, this should be further researched<br>• There is a lot of controversy around the use of methadone. This should not be a long-term solution, but still is.<br>• It was agreed that the relationship with the judge/sheriff is important. Glasgow provides some good examples of this but often the treatment order does not take into account the personal background and needs of the individual – tailoring of treatment orders would improve compliance<br>• Possessing specific knowledge of treatment orders and the individual involved is important in gaining respect and trust.<br><br>Co-authorship of the journal article publications was discussed with the group, and how we could work together. They were considering being involved.<br><br>**PPI Meeting 7th July 2024 (mainly dedicated to the quantitative review)**<br><br>The group were given an update on the review, and we shared findings from the quantitative review including:<br><br>• Number of studies, geographical location and number of participants.<br>• Summary of participant characteristics including drug and alcohol use and criminal history and interactions with the criminal justice system.<br>• Summary of equity factors that might impact on the findings<br>• Summary of the types of outcome measures that had been used across the studies<br><br>The group were asked to provide their perspectives on the review findings and specifically asked about the outcomes that measured family relationships/conflict measures. Discussion followed about whether these outcomes are useful measures and what information is currently missing from the included studies. The meeting also had a brief discussion about gaps in evidence and how the studies in the quantitative review may not provide a complete picture of what actually happens in real-life.<br><br>Two members are keen to be involved in co-authorship of the PPI section. One member is not keen, two members were not present.<br><br>See Table C for a summary, using descriptors from ACTIVE framework.

Table C Summary of PPI / stakeholder involvement in the reviews using the ACTIVE framework

		What happened?		Stage and level of involvement**										
**Who was involved?**	**How were they recruited?**	**Approach**	**Method**	**1**	**2**	**3**	**4**	**5**	**6**	**7**	**8**	**9**	**10**	**11**
Stakeholder Group	Closed; existing group	Multiple-time closed event approach	Direct interaction	Con	Ctb	Ctb	Ctb		Ctb			(R)	I	
PPI Advisory Group*	Closed; invitation only	Multiple-time closed event approach	Direct interaction						Ctb			R	I	

Patients*: patients, carers and/or their families; Con: controlling; I: influencing; Ctb: contributing; R: receiving. Blank cells in 'Stage of involvement' indicate that there was no stakeholder/PPI involvement.

Stage/level using Cochrane Collaboration 1. development of question; 2. Plan methods; 3. Write & publish protocol; 4. Develop search; 5. Run search; 6. Select studies; 7. Collect data; 8. Assess risk of bias; 9. Analyse data; 10. Interpret findings; 11. Write/publish review
