# Supplementary material for: Effectiveness of legally mandated non-custodial drug and alcohol treatment orders for improved health, well-being, global functioning and quality of life: a systematic review and meta-analysis
Source: Health Justice. 2026 Jan 27;14:11. doi: 10.1186/s40352-025-00354-4 (PMC12958499; doi:10.1186/s40352-025-00354-4)
Supplement: Supplementary file 3 — Additional file 3. Search strategies. The terms used to search the 14 electronic databases [file 40352_2025_354_MOESM3_ESM.pdf]

Additional file 3. Search strategies

Source	Version/Platform/URL	Date of Search	Records retrieved	
MEDLINE® and In-Process, In-Data_Review & Other Non-Indexed Citations 1946-October 30 2023	(Ovid SP)	31.10.23	846	
Embase	(Ovid SP)	31.10.23	1197	
Psycinfo	(Ovid SP)	31.10.23	1535	
CINAHL	EBSCOhost (could not save search)	31.10.23	805	
Web of Science	Clarivate	31.10.23	399	
Lexis Library		6.11.23	181	
Westlaw UK	(word doc export only)	6.11.23	81	
ASSIA	Proquest	1.11.23	102	
IBSS	Proquest	1.11.23	41	
Policy Commons		7.11.23	274	
Social Care Online		7.11.23	88	
WHO ICTRP		6.11.23	15	
ClinicalTrials.gov.		6.11.23	167	

MEDLINE via OVID	1 exp Substance-Related Disorders/pc, rh<br>2 (substance adj ("use" or misuse or abuse or problem* or disorder* or addiction* or dependen*)).ti,ab.<br>3 (Alcohol* adj ("use" or misuse or abuse or problem* or disorder* or addiction* or dependen*)).ti,ab.<br>4 (Drug* adj ("use" or misuse or abuse or problem* or disorder* or addiction* or dependen*)).ti,ab.<br>5 (Opioid* adj ("use" or misuse or abuse or problem* or disorder* or addiction* or dependen*)).ti,ab.<br>6 SUD.ti,ab.<br>7 "Alcoholic ketoacidosis".ti,ab.<br>8 or/1-7<br>9 "12 step programme*".ti,ab.<br>10 "alcohol treatment*".ti,ab.<br>11 "cognitive behavior* therapy".ti,ab.<br>12 "community based intervention*".ti,ab.<br>13 "community reinforcement".ti,ab.<br>14 detoxification.ti,ab.<br>15 "drug counselling".ti,ab.<br>16 "mandatory test*".ti,ab.<br>17 "medication assisted treatment*".ti,ab.<br>18 "non custodial".ti,ab.<br>19 "problem solving court*".ti,ab.<br>20 "random test*".ti,ab.<br>21 "regular test*".ti,ab.<br>22 "substance use treatment*".ti,ab.<br>23 "treatment order*".ti,ab.<br>24 "twelve step program*".ti,ab.<br>25 "recovery capital".ti,ab.<br>26 "therapeutic jurisprudence".ti,ab.<br>27 "Community payback order*".ti,ab.<br>28 or/9-27	846

	29 exp Criminal Law/<br>30 exp Law Enforcement/mt<br>31 exp Driving Under the Influence/pc<br>32 "criminal justice".ti,ab.<br>33 Crime/pc<br>34 crime*.ti,ab.<br>35 criminal.ti,ab.<br>36 judicial*.ti,ab.<br>37 or/29-36<br>38 8 and 28 and 37<br>39 "Abstinence tag*".ti,ab.<br>40 "Addressing substance related offending".ti,ab.<br>41 "Adult treatment court*".ti,ab.<br>42 "Alcohol abstinence monitoring requirement*".ti,ab.<br>43 "Alcohol abstinence tag*".ti,ab.<br>44 "Alcohol court*".ti,ab.<br>45 "Alcohol monitoring tag*".ti,ab.<br>46 "Alcohol rehabilitation requirement*".ti,ab.<br>47 "Alcohol specified activity requirement".ti,ab.<br>48 "Alcohol tag*".ti,ab.<br>49 "Alcohol treatment requirement*".ti,ab.<br>50 "Driving under the influence court".ti,ab.<br>51 "Driving while intoxicated court*".ti,ab.<br>52 "Drug abstinence order*".ti,ab.<br>53 "Drug abstinence requirement*".ti,ab.<br>54 "Drug court*".ti,ab.<br>55 "Drug rehabilitation requirement*".ti,ab.<br>56 "Drug treatment and testing order*".ti,ab.<br>57 "Drug treatment court*".ti,ab.<br>58 "Electronic alcohol tag*".ti,ab.<br>59 "Family drug and alcohol court*".ti,ab.<br>60 "Low intensity alcohol program".ti,ab.	

	61 "mandatory alcohol treatment*".ti,ab.<br>62 "Sobriety court*".ti,ab.<br>63 "Sobriety project".ti,ab.<br>64 AAMR.ti,ab.<br>65 DTTO.ti,ab.<br>66 or/39-65<br>67 38 or 66<br>68 limit 67 to english language	

Embase via OVID	1 exp drug dependence/pc, rh [Prevention, Rehabilitation]<br>2 (substance adj ("use" or misuse or abuse or problem* or disorder* or addiction* or dependen*)).ti,ab.<br>3 (Alcohol* adj ("use" or misuse or abuse or problem* or disorder* or addiction* or dependen*)).ti,ab.<br>4 (Drug* adj ("use" or misuse or abuse or problem* or disorder* or addiction* or dependen*)).ti,ab.<br>5 (Opioid* adj ("use" or misuse or abuse or problem* or disorder* or addiction* or dependen*)).ti,ab.<br>6 SUD.ti,ab.<br>7 "Alcoholic ketoacidosis".ti,ab.<br>8 or/1-7<br>9 "12 step programme*".ti,ab.<br>10 "alcohol treatment*".ti,ab.<br>11 "cognitive behavior* therapy".ti,ab.<br>12 "community based intervention*".ti,ab.<br>13 "community reinforcement".ti,ab.<br>14 detoxification.ti,ab.<br>15 "drug counselling".ti,ab.<br>16 "mandatory test*".ti,ab.<br>17 "medication assisted treatment*".ti,ab.<br>18 "non custodial".ti,ab.<br>19 "problem solving court*".ti,ab.<br>20 "random test*".ti,ab.<br>21 "regular test*".ti,ab.<br>22 "substance use treatment*".ti,ab.<br>23 "treatment order*".ti,ab.<br>24 "twelve step program*".ti,ab.<br>25 "recovery capital".ti,ab.<br>26 "therapeutic jurisprudence".ti,ab.<br>27 "Community payback order*".ti,ab.<br>28 or/9-27	1197

	29 exp criminal law/<br>30 exp law enforcement/<br>31 exp drunken driving/<br>32 "criminal justice".ti,ab.<br>33 crime prevention/<br>34 crime*.ti,ab.<br>35 criminal.ti,ab.<br>36 judicial*.ti,ab.<br>37 or/29-36<br>38 8 and 28 and 37<br>39 "Abstinence tag*".ti,ab.<br>40 "Addressing substance related offending".ti,ab.<br>41 "Adult treatment court*".ti,ab.<br>42 "Alcohol abstinence monitoring requirement*".ti,ab.<br>43 "Alcohol abstinence tag*".ti,ab.<br>44 "Alcohol court*".ti,ab.<br>45 "Alcohol monitoring tag*".ti,ab.<br>46 "Alcohol rehabilitation requirement*".ti,ab.<br>47 "Alcohol specified activity requirement".ti,ab.<br>48 "Alcohol tag*".ti,ab.<br>49 "Alcohol treatment requirement*".ti,ab.<br>50 "Driving under the influence court".ti,ab.<br>51 "Driving while intoxicated court*".ti,ab.<br>52 "Drug abstinence order*".ti,ab.<br>53 "Drug abstinence requirement*".ti,ab.<br>54 "Drug court*".ti,ab.<br>55 "Drug rehabilitation requirement*".ti,ab.<br>56 "Drug treatment and testing order*".ti,ab.<br>57 "Drug treatment court*".ti,ab.<br>58 "Electronic alcohol tag*".ti,ab.<br>59 "Family drug and alcohol court*".ti,ab.<br>60 "Low intensity alcohol program".ti,ab.	

	61 "mandatory alcohol treatment*".ti,ab.<br>62 "Sobriety court*".ti,ab.<br>63 "Sobriety project".ti,ab.<br>64 AAMR.ti,ab.<br>65 DTTO.ti,ab.<br>66 or/39-65<br>67 38 or 66<br>68 limit 67 to english language	

Psycinfo via OVID	1 exp "Substance Use Disorder"/<br>2 (substance adj ("use" or misuse or abuse or problem* or disorder* or addiction* or dependen*)).ti,ab.<br>3 (Alcohol* adj ("use" or misuse or abuse or problem* or disorder* or addiction* or dependen*)).ti,ab.<br>4 (Drug* adj ("use" or misuse or abuse or problem* or disorder* or addiction* or dependen*)).ti,ab.<br>5 (Opioid* adj ("use" or misuse or abuse or problem* or disorder* or addiction* or dependen*)).ti,ab.<br>6 SUD.ti,ab.<br>7 "Alcoholic ketoacidosis".ti,ab.<br>8 or/1-7<br>9 "12 step programme*".ti,ab.<br>10 "alcohol treatment*".ti,ab.<br>11 "cognitive behavior* therapy".ti,ab.<br>12 "community based intervention*".ti,ab.<br>13 "community reinforcement".ti,ab.<br>14 detoxification.ti,ab.<br>15 "drug counselling".ti,ab.<br>16 "mandatory test*".ti,ab.<br>17 "medication assisted treatment*".ti,ab.<br>18 "non custodial".ti,ab.<br>19 "problem solving court*".ti,ab.<br>20 "random test*".ti,ab.<br>21 "regular test*".ti,ab.<br>22 "substance use treatment*".ti,ab.<br>23 "treatment order*".ti,ab.<br>24 "twelve step program*".ti,ab.<br>25 "recovery capital".ti,ab.<br>26 "therapeutic jurisprudence".ti,ab.<br>27 "Community payback order*".ti,ab.<br>28 or/9-27	1535

	29 exp Criminal Law/<br>30 exp Law Enforcement/<br>31 exp Driving Under the Influence/<br>32 "criminal justice".ti,ab.<br>33 exp Crime/<br>34 crime*.ti,ab.<br>35 criminal.ti,ab.<br>36 judicial*.ti,ab.<br>37 or/29-36<br>38 8 and 28 and 37<br>39 "Abstinence tag*".ti,ab.<br>40 "Addressing substance related offending".ti,ab.<br>41 "Adult treatment court*".ti,ab.<br>42 "Alcohol abstinence monitoring requirement*".ti,ab.<br>43 "Alcohol abstinence tag*".ti,ab.<br>44 "Alcohol court*".ti,ab.<br>45 "Alcohol monitoring tag*".ti,ab.<br>46 "Alcohol rehabilitation requirement*".ti,ab.<br>47 "Alcohol specified activity requirement".ti,ab.<br>48 "Alcohol tag*".ti,ab.<br>49 "Alcohol treatment requirement*".ti,ab.<br>50 "Driving under the influence court".ti,ab.<br>51 "Driving while intoxicated court*".ti,ab.<br>52 "Drug abstinence order*".ti,ab.<br>53 "Drug abstinence requirement*".ti,ab.<br>54 "Drug court*".ti,ab.<br>55 "Drug rehabilitation requirement*".ti,ab.<br>56 "Drug treatment and testing order*".ti,ab.<br>57 "Drug treatment court*".ti,ab.<br>58 "Electronic alcohol tag*".ti,ab.<br>59 "Family drug and alcohol court*".ti,ab.<br>60 "Low intensity alcohol program".ti,ab.	

	61 "mandatory alcohol treatment*".ti,ab.<br>62 "Sobriety court*".ti,ab.<br>63 "Sobriety project".ti,ab.<br>64 AAMR.ti,ab.<br>65 DTTO.ti,ab.<br>66 or/39-65<br>67 38 or 66<br>68 limit 67 to english language	

CINAHL via EBSCO	S66 S37 OR S65<br>S65 S38 OR S39 OR S40 OR S41 OR S42 OR S43 OR S44 OR S45 OR S46 OR S47 OR S48 OR S49 OR S50 OR S51 OR S52 OR S53 OR S54 OR S55 OR S56 OR S57 OR S58 OR S59 OR S60 OR S61 OR S62 OR S63 OR S64<br>S64 TX DTTO<br>S63 TX AAMR<br>S62 TX "Sobriety project"<br>S61 TX "Sobriety court*"<br>S60 TX "mandatory alcohol treatment*"<br>S59 TX "Low intensity alcohol program"<br>S58 TX "Family drug and alcohol court*"<br>S57 TX "Electronic alcohol tag*"<br>S56 TX "Drug treatment court*"<br>S55 TX "Drug treatment and testing order*"<br>S54 TX "Drug rehabilitation requirement*"<br>S53 TX "Drug court*"<br>S52 TX "Drug abstinence requirement*"<br>S51 TX "Drug abstinence order*"<br>S50 TX "Driving while intoxicated court*"<br>S49 TX "Driving under the influence court"<br>S48 TX "Alcohol treatment requirement*"<br>S47 TX "Alcohol tag*"<br>S46 TX "Alcohol specified activity requirement"<br>S45 TX "Alcohol rehabilitation requirement*"<br>S44 TX "Alcohol monitoring tag*"<br>S43 TX "Alcohol court*"<br>S42 TX "Alcohol abstinence tag*"<br>S41 TX "Alcohol abstinence monitoring requirement*"<br>S40 TX "Adult treatment court*"<br>S39 TX "Addressing substance related offending"	805

	S38 TX "Abstinence tag*"<br>S37 S8 AND S28 AND S36<br>S36 S29 OR S30 OR S31 OR S32 OR S33 OR S34 OR S35<br>S35 TX judicial*<br>S34 TX criminal<br>S33 TX crime*<br>S32 (MH "Crime")<br>S31 TX "criminal justice"<br>S30 (MH "Driving While Intoxicated")<br>S29 (MH "Criminal Justice")<br>S28 S9 OR S10 OR S11 OR S12 OR S13 OR S14 OR S15 OR S16 OR S17 OR S18 OR S19 OR S20 OR S21 OR S22 OR S23 OR S24 OR S25 OR S26 OR S27<br>S27 TX "Community payback order*"<br>S26 TX "therapeutic jurisprudence"<br>S25 TX "recovery capital"<br>S24 TX "twelve step program*"<br>S23 TX "treatment order*"<br>S22 TX "substance use treatment*"<br>S21 TX "regular test*"<br>S20 TX "random test*"<br>S19 TX "problem solving court*"<br>S18 TX "non custodial"<br>S17 TX "medication assisted treatment*"<br>S16 TX "mandatory test*"<br>S15 TX "drug counselling"<br>S14 TX detoxification<br>S13 TX "community reinforcement"<br>S12 TX "community based intervention*"<br>S11 TX "cognitive behavior* therapy"<br>S10 TX "alcohol treatment*"	

	S9 TX "12 step programme*"<br>S8 S1 OR S2 OR S3 OR S4 OR S5 OR S6 OR S7<br>S7 TX "Alcoholic ketoacidosis"<br>S6 TX SUD<br>S5 TX Opioid* N ("use" or misuse or abuse or problem* or disorder* or addiction* or dependen*)<br>S4 TX Drug* N ("use" or misuse or abuse or problem* or disorder* or addiction* or dependen*)<br>S3 TX Alcohol* N ("use" or misuse or abuse or problem* or disorder* or addiction* or dependen*)<br>S2 TX substance N ("use" or misuse or abuse or problem* or disorder* or addiction* or dependen*)<br>S1 (MH "Substance Use Disorders+")	

Web of Science via Clarivate	((((((((((((((((((((((((ALL=("Abstinence tag*")) OR ALL=("Addressing substance related offending")) OR ALL=("Adult treatment court*")) OR ALL=("Alcohol abstinence monitoring requirement*")) OR ALL=("Alcohol abstinence tag*")) OR ALL=("Alcohol court*")) OR ALL=("Alcohol monitoring tag*")) OR ALL=("Alcohol rehabilitation requirement*")) OR ALL=("Alcohol specified activity requirement")) OR ALL=("Alcohol tag*")) OR ALL=("Alcohol treatment requirement*")) OR ALL=("Driving under the influence court")) OR ALL=("Driving while intoxicated court*")) OR ALL=("Drug abstinence order*")) OR ALL=("Drug abstinence requirement*")) OR ALL=("Drug court*")) OR ALL=("Drug rehabilitation requirement*")) OR ALL=("Drug treatment and testing order*")) OR ALL=("Drug treatment court*")) OR ALL=("Electronic alcohol tag*")) OR ALL=("Family drug and alcohol court*")) OR ALL=("Low intensity alcohol program")) OR ALL=("mandatory alcohol treatment*")) OR ALL=("Sobriety court*")) AND ALL=(“family conflict” OR “Global functioning” OR “quality of life” OR “risky behavior*” OR “risky behaviors*” OR “risky behaviour*” OR “social participation” OR “well being” OR anxiety OR death OR depression OR fatalit* OR health* OR homelessness OR Morbidity OR Mortality OR overdose)	399

LexisLibrary	("Abstinence tag*" OR "Addressing substance related offending" OR "Adult treatment court*" OR "Alcohol abstinence monitoring requirement*" OR "Alcohol abstinence tag*" OR "Alcohol court*" OR "Alcohol monitoring tag*" OR "Alcohol rehabilitation requirement*" OR "Alcohol specified activity requirement" OR "Alcohol tag*" OR "Alcohol treatment requirement*" OR "Driving under the influence court" OR "Driving while intoxicated court*" OR "Drug abstinence order*" OR "Drug abstinence requirement*" OR "Drug court*" OR "Drug rehabilitation requirement*" OR "Drug treatment and testing order*" OR "Drug treatment court*" OR "Electronic alcohol tag*" OR "Family drug and alcohol court*" OR "Low intensity alcohol program" OR "mandatory alcohol treatment*" OR "Sobriety court*" OR "Sobriety project" OR AAMR OR DTTO) AND ("family conflict" OR "Global functioning" OR "quality of life" OR "risky behavior*" OR "risky behavior*" OR "risky behaviour*" OR "social participation" OR "well being" OR anxiety OR death OR depression OR fatalit* OR health* OR homelessness OR Morbidity OR Mortality OR overdose) Pub type Books and Journals	181
Westlaw UK	"Drug treatment court*" OR "Drug court*" OR "substance use treatment*" OR "treatment order*" AND Health OR death OR overdose JOURNALS SEARCH	81

ASSIA	S1 summary("Abstinence tag*") OR summary("Addressing substance related offending") OR summary("Adult treatment court*") OR summary("Alcohol abstinence monitoring requirement*") OR summary("Alcohol abstinence tag*") OR summary("Alcohol court*") OR summary("Alcohol monitoring tag*") OR summary("Alcohol rehabilitation requirement*") OR summary("Alcohol specified activity requirement") OR summary("Alcohol tag*")<br>S2 summary("Alcohol treatment requirement*") OR summary("Driving under the influence court") OR summary("Driving while intoxicated court*") OR summary("Drug abstinence order*") OR summary("Drug abstinence requirement*") OR summary("Drug court*") OR summary("Drug rehabilitation requirement*") OR summary("Drug treatment and testing order*") OR summary("Drug treatment court*") OR summary("Electronic alcohol tag*")<br>S3 summary("Family drug and alcohol court*") OR summary("Low intensity alcohol program") OR summary("mandatory alcohol treatment*") OR summary("Sobriety court*") OR summary("Sobriety project")<br>S4 [S1] OR [S2] OR [S3]<br>S5 summary(“family conflict” OR “Global functioning” OR “quality of life” OR “risky behavior*” OR “risky behaviors*” OR “risky behaviour*” OR “social participation” OR “well being” OR anxiety OR death OR depression OR fatalit* OR health* OR homelessness OR Morbidity OR Mortality OR overdose)<br>S6 [S4] AND [S5]	102

IBSS	S1 (summary("Abstinence tag*") OR summary("Addressing substance related offending") OR summary("Adult treatment court*") OR summary("Alcohol abstinence monitoring requirement*") OR summary("Alcohol abstinence tag*") OR summary("Alcohol court*") OR summary("Alcohol monitoring tag*") OR summary("Alcohol rehabilitation requirement*") OR summary("Alcohol specified activity requirement") OR summary("Alcohol tag*")) OR (summary("Alcohol treatment requirement*") OR summary("Driving under the influence court") OR summary("Driving while intoxicated court*") OR summary("Drug abstinence order*") OR summary("Drug abstinence requirement*") OR summary("Drug court*") OR summary("Drug rehabilitation requirement*") OR summary("Drug treatment and testing order*") OR summary("Drug treatment court*") OR summary("Electronic alcohol tag*")) OR (summary("Family drug and alcohol court*") OR summary("Low intensity alcohol program") OR summary("mandatory alcohol treatment*") OR summary("Sobriety court*") OR summary("Sobriety project"))<br>S2 summary("family conflict" OR "Global functioning" OR "quality of life" OR "risky behavior*" OR "risky behaviors*" OR "risky behaviour*" OR "social participation" OR "well being" OR anxiety OR death OR depression OR fatalit* OR health* OR homelessness OR Morbidity OR Mortality OR overdose)<br>S3 [S1] AND [S12]	41

Policy Commons	(Drug treatment court*' OR summary:'Drug court*' OR summary:'treatment order*') TOPIC health	274
Social Care Online	“Drug treatment court*” OR “Drug court*” OR “alcohol treatment court*” OR “alcohol court*” OR “Driving under the influence court*” OR “Alcohol tag*”	88
WHO ICTRP	(TITLE) Drug treatment court$ OR Drug court$ OR alcohol treatment court$ OR alcohol court$ OR Driving under the influence court OR Alcohol tag$ (TI) OR (INTERVENTION) Drug treatment court$ OR Drug court$ OR alcohol treatment court$ OR alcohol court$ OR Driving under the influence court OR Alcohol tag$ (	15
ClinicalTrials.gov.	Drug treatment court OR Drug court OR alcohol treatment court OR alcohol court OR Driving under the influence court OR Alcohol tag	167
TOTAL before de-duplication		
