# Supplementary material for: Effectiveness of legally mandated non-custodial drug and alcohol treatment orders for improved health, well-being, global functioning and quality of life: a systematic review and meta-analysis
Source: Health Justice. 2026 Jan 27;14:11. doi: 10.1186/s40352-025-00354-4 (PMC12958499; doi:10.1186/s40352-025-00354-4)
Supplement: Supplementary file 4 — Additional file 4. Deviations from protocol. Description of changes from the protocol [file 40352_2025_354_MOESM4_ESM.pdf]

Additional file 4. Deviations from protocol

1. We searched an additional database the National Criminal Justice Reference Service (NCJRS) when supplementary searches resulted in us identifying new eligible studies which were indexed in this database. Driving while intoxicated (DWI)/ Driving under the influence (DUI) were not among our original search terms, therefore the information specialist (CT) undertook additional searches of the clinical trials register and the ICTRP for any ongoing DUI/DWI trials – no new studies were identified.
2. We also intended to extract data under all ICD-11 categories (ICD-11, 2024) but we prioritised the most relevant items in collaboration with our stakeholders since not all categories were relevant.
3. We had also planned to present our meta-analyses within two comparisons based on when the outcomes had been recorded (i.e. recorded at the end of the intervention (‘immediate’ point) and outcomes measured at a ‘follow-up’ time point). However, it was not always possible to determine when the intervention had finished as sentence lengths varied across studies and individual participants. Therefore, we limited our meta-analyses to studies that reported outcomes measured during the delivery of the intervention and at the final follow-up time-point reported within the main study.
4. We had planned to conduct a series of subgroup analysis as part of the equity analysis but there was insufficient data to allow us to do this.

References

ICD-11. (2024). *International Classification of Diseases 11th Revision*. Retrieved 27 September from https://icd.who.int/en
