# Supplementary material for: Effectiveness of legally mandated non-custodial drug and alcohol treatment orders for improved health, well-being, global functioning and quality of life: a systematic review and meta-analysis
Source: Health Justice. 2026 Jan 27;14:11. doi: 10.1186/s40352-025-00354-4 (PMC12958499; doi:10.1186/s40352-025-00354-4)
Supplement: Supplementary file 6 — Additional file 6. Summary of included studies methods and selection criteria. The methods and selection criteria used within the included studies [file 40352_2025_354_MOESM6_ESM.pdf]

Additional file 6. Summary of included studies methods and selection criteria

Key: CBA: controlled before-and after; DUI: driving under the influence; FTDO: first time drug offenders; NR: not reported; RCT: randomised controlled trial; SUD: substance use disorder(s)

Study year	Design	Country	Aim (verbatim)	Inclusion criteria	Exclusion criteria	Details of dropouts/withdrawals (if reported)	Funding	Conflict of interest
(Deschenes et al., 1995)	RCT	USA	The main objectives of the experiment were:<br>1. To evaluate the effect of different types of treatment and sanctions on offender reintegration and system overcrowding<br>2. To determine whether the frequency of drug testing has an effect on offender reintegration and criminal behaviour	First-time felony drug offenders with similar needs for treatment. Thus, the FTDO Program was limited to felons who were sentenced to probation for a first conviction for possession of marijuana, dangerous drugs, narcotics, or drug paraphernalia	Defendants convicted of drug sales or transportation. In addition, offenders who were sentenced to special programs within probation, such as intensive supervision, residential treatment, or the Community Punishment Program, were excluded.	9	Supported by Grant Number 91-DD-CX-K050 awarded to RAND by the National Institute of Justice, U.S. Department of Justice	NR
(Desland & Batey, 1992)	CBA	Australia	The aims of this project were (i) a comparison of referral groups	NR	NR	NR	This research was funded by the NSW Directorate of the Drug	NR

Study year	Design	Country	Aim (verbatim)	Inclusion criteria	Exclusion criteria	Details of dropouts/withdrawals (if reported)	Funding	Conflict of interest
			at presentation to determine whether the earlier health intervention occurred in this context. In particular a comparison of demographic, heroin use and criminality variables. (2) To compare each group for ~2 months on measure of heroin use, methadone treatments, criminality and employment.				Offensive. The authors wish to extend their thanks to the numerous agencies, departments and individuals who co-operated with the undertaking of this project.	
(Festinger et al., 2016)	RCT	USA	This study evaluates the efficacy of a computer-facilitated HIV prevention intervention for drug court clients.	(1) be at least 18 years of age, (2) be charged with a non-violent felony offense, (3) have no more than two prior non-violent convictions, juvenile adjudications or diversionary	NR	NR	Financial support: NIDA Grant #R01-DA-030257.	NR

Study year	Design	Country	Aim (verbatim)	Inclusion criteria	Exclusion criteria	Details of dropouts/withdrawals (if reported)	Funding	Conflict of interest
				opportunities, (4) be in need of treatment for drug abuse or dependence as assessed by a clinical case manager employed by the court, and (5) volunteer to participate in the drug court program for at least 12 months.				
(Gottfredson & Exum, 2002)	RCT	USA	The study's authors recommended repeating the evaluation with a longer follow-up period, a larger number of study participants, and random assignment of BDTC-eligible study participants to treatment and control conditions.	Psychopathy Checklist is used to screen out offenders who are likely to be unsuitable for the program	NR	Different depending on which follow-up time-point and which paper - need to extract accordingly	Maryland Department of Public Safety and Correctional Services	NR

Study year	Design	Country	Aim (verbatim)	Inclusion criteria	Exclusion criteria	Details of dropouts/withdrawals (if reported)	Funding	Conflict of interest
			The results of this second study are reported here.					
(Green & Rempel, 2012)	NRT	USA	This study seeks to test the impact of drug court participation on outcomes in four domains: (a) socioeconomic well-being (employment, education, income, and perceived financial assistance needs), (b) family relationships, (c) mental health, and (d) homelessness. Second, this study seeks to explore which, if any, offender characteristics other than drug	NR	NR	Among those interviewed at baseline, follow-up interviews were successfully conducted with 1,533 offenders at 6 months (87% of drug court and 84% of comparison), 1,474 offenders at 18 months (82% of drug court and 84% of comparison), and 1,349 offenders at both follow-up periods (an identical 76% of both samples).	This research was supported by the National Institute of Justice (NIJ) of the U.S. Department of Justice (Grant No. 2003-DC-BX-1001)	The author(s) declared no potential conflicts of interest with respect to the research, authorship, and/or publication of this article

Study year	Design	Country	Aim (verbatim)	Inclusion criteria	Exclusion criteria	Details of dropouts/withdrawals (if reported)	Funding	Conflict of interest
			court participation, such as offender demographics, prior drug use or mental health status at baseline, and prior criminality, are associated with greater or lesser psychosocial problems at follow-up					
(Harrell et al., 1998)	RCT	USA	This evaluation extends these findings by examining the use of judicial monitoring, frequent drug testing, treatment and graduated sanctions, and early intervention with drug	Eligible for the specialized services on the basis of having at least two drug test failures following their pretrial release. The PSA staff flagged the files of eligible defendants before each hearing	Unlike many drug courts that exclude defendants who have been convicted for a violent offense or face pending charges for a violent offense (required for federal drug court funding), the programs were open to those with long criminal histories as well as first offenders. Unlike many drug courts, the program was not limited to addicts, because eligibility for the	50% dropped out of drug testing in each group (Exhibit 10.1, page 131)	This research was supported by the National Institute of Justice under Grant No. 94-IJ-CX-K011. Funds were provided by the Center on Substance Abuse Treatment of the Substance Abuse and Mental Health	NR

Study year	Design	Country	Aim (verbatim)	Inclusion criteria	Exclusion criteria	Details of dropouts/withdrawals (if reported)	Funding	Conflict of interest
					program was based on drug tests results, not individualized assessments of addiction. As a result, the program participants varied widely in the severity and duration of their drug use		Services Administration of DHHS under interagency transfer	
(Harrell et al., 2001)	NRT/CBA	USA	The impact evaluation tested the general hypothesis that BTC participants were less likely than a comparison group of similar women to use drugs, commit crimes, and experience social, health, and employment problems during the follow-up period	Referral to BTC was limited to defendants arrested on a drug felony charge in three participating prosecution zones (out of five total zones) in Brooklyn. The courts make a systematic effort to screen incoming cases for eligibility and offer drug court services to all who are found eligible.	Those who had a prior conviction or pending charges for a violent offense and those involved in certain kinds of cases deemed ineligible by the District Attorney (such as drug sales on or near school grounds or offenses that suggested involvement in higher-level drug trafficking) were excluded.	35 (BTC), 7 (comparator)	NR	NR

**Study year**	**Design**	**Country**	**Aim (verbatim)**	**Inclusion criteria**	**Exclusion criteria**	**Details of dropouts/withdrawals (if reported)**	**Funding**	**Conflict of interest**
(Jones, 2013)	RCT	Australia	To assess whether there is any evidence that increasing the level of supervision for participants in this drug court has any effect on (a) early-phase substance use, (b) early-phase sanctioning rates, (c) likelihood of progressing to Phase 2 of the program, and (d) likelihood of being terminated from the program and returning to custody.	Eligible and suitable to take part in the program are required to pass through three phases of treatment before they are eligible to graduate (initiation, consolidation, and reintegration).	NR	24 withdrawn (n = 14 IJS and n = 10 SAU participants). 3 participants (n = 2 IJS; n = 1 SAU) were found to be ineligible after they had been held over for sentence and were excluded from participation in the program. No outcome data were available for these 3 participants, and they were excluded from all analyses. A further 21 participants (n = 12 IJS; n = 9 SAU) were treated in residential rehabilitation facilities and did not have any level of face-to-face judicial supervision. These 21 participants were excluded from the analyses in this study.	NR	NR
(MacDonald et al., 2007)	RCT	USA	Our evaluation focuses on whether the DUI court reduced the incidence of subsequent	Individuals convicted of a second or third misdemeanour DUI offense in any of the six divisions of the	NR	1	Grant from the National Institute on Alcoholism and Alcohol Abuse (R01 AA12457).	NR

Study year	Design	Country	Aim (verbatim)	Inclusion criteria	Exclusion criteria	Details of dropouts/withdrawals (if reported)	Funding	Conflict of interest
			DUI arrests, self-reported drinking and driving, and problem drinking. In addition, we examine whether the DUI court program was associated with increased completion of court-ordered	Rio Hondo Municipal Court between May 2000 and December 2002 were eligible to participate in the study if the judge before whom their case was heard determined that no special circumstances prohibited study participation.				
(NCT02978417, 2016)	RCT	USA	"Two primary aims of the feasibility study will be to (1) pilot-test the delivery of Vivitrol treatment for 10-20 interested and eligible clients of the Wake County drug court; and (2) in parallel with the pilot administration of Vivitrol, study a range	• Client of Wake County Drug Treatment Court<br>• Interested in medication-assisted treatment for opioid dependence<br>• 18-65 years old<br>• understands and speaks English<br>• understands that study	• pregnant (i.e., has a positive pregnancy test), planning to become pregnant, or breastfeeding during the study<br>• positive urine drug test for opioids, buprenorphine or methadone at the beginning of treatment and before each Vivitrol injection<br>• used any opioid drug within 10 days prior to treatment	2 in each arm	69665 Laura and John Arnold Foundation	NR

Study year	Design	Country	Aim (verbatim)	Inclusion criteria	Exclusion criteria	Details of dropouts/withdrawals (if reported)	Funding	Conflict of interest
			of feasibility issues for scaling this research to a RCT that would examine court processes, treatment delivery, and relevant clinical and justice outcomes, in a study design that provides more definitive results and also addresses IRB-related concerns in working with this particularly vulnerable, court-involved population	participation is fully voluntary, with no effect on court standing<br>• willing and able to give written informed consent<br>• has an opioid use disorder (DSM-5 diagnosis of moderate or severe opioid use disorder)<br>• has at least 6 months remaining before anticipated Drug Court graduation<br>• (if female) does not intend to become pregnant or breastfeeding during the	• a condition, disease state, previous medical history, or observed abnormalities (including physical examination, laboratory evaluation [e.g., kidney or liver function test result], or urinalysis finding) at screening that, in the opinion of the investigator, would preclude safe participation in the study or affect the ability of the subject to adhere to the protocol visit schedule, fulfill visit requirements, or would interfere with the study assessments, including, but not limited to, the following: uncontrolled hypertension or diabetes, renal disease/impairment,			

Study year	Design	Country	Aim (verbatim)	Inclusion criteria	Exclusion criteria	Details of dropouts/withdrawals (if reported)	Funding	Conflict of interest
				study period and is willing to adhere to contraception requirements during the study period is willing to adhere to the study requirements<br>at least 7-10 days without opioid use before beginning extended-release injectable naltrexone given that detoxification from opioids before initiating or resuming extended-release injectable naltrexone is necessary to prevent withdrawal.	stroke, seizures or neurological disorder, cardiovascular (eg, endocarditis), neoplastic disease<br>• chronic pain condition requiring ongoing opioid analgesia Aspartate aminotransferase or alanine aminotransferase value more than times the upper limit of normal<br>• contraindicated medical condition per the approved labelling for naltrexone<br>• DSM-5 diagnosis within the past 12 months of other psychiatric conditions or disorders that, in the investigator's opinion, could interfere with participation in the study			

Study year	Design	Country	Aim (verbatim)	Inclusion criteria	Exclusion criteria	Details of dropouts/withdrawals (if reported)	Funding	Conflict of interest
					• currently physiologically dependent on any psychoactive substance (except caffeine, or tobacco) requiring medical intervention for detoxification<br>• history of hypersensitivity or adverse reaction to naltrexone, or naloxone<br>• significant suicidal ideation or behaviour within the past year, as assessed with the Patient Health Questionnaire (PHQ-9)			
(Rodriguez-Monguio et al., 2021)	CBA	USA	To evaluate BSAS treatment services utilization and expenditures and relapse and recidivism rates and to identify	NR	NR	NA - matched sample	Massachusetts Executive Office of the Trial Court	None

Study year	Design	Country	Aim (verbatim)	Inclusion criteria	Exclusion criteria	Details of dropouts/withdrawals (if reported)	Funding	Conflict of interest
			predictors of cost of provision of treatment services in a cohort of MA probationers in the drug court system compared to a matched cohort of probationers with documented SUD in the traditional court system					

References

Deschenes, E. P., Turner, S., & Greenwood, P. W. (1995). Drug court or probation? An experimental evaluation of Maricopa County's drug court. *Justice System Journal 18*(1), 55-73.

Desland, M. L., & Batey, R. G. (1992). A 12-month prospective comparison of court-diverted with self-referred heroin users. *Drug Alcohol Rev*, *11*(2), 121-129. https://doi.org/10.1080/09595239200185591

Festinger, D. S., Dugosh, K. L., Kurth, A. E., & Metzger, D. S. (2016). Examining the efficacy of a computer facilitated HIV prevention tool in drug court. *Drug Alcohol Depend*, *162*, 44-50. https://doi.org/10.1016/j.drugalcdep.2016.02.026

Gottfredson, D. C., & Exum, M. L. (2002). The Baltimore City Drug Treatment Court: One year results from a randomized study. *Journal of Research in Crime and Delinquency 39*(3), 337-356.

Green, M., & Rempel, M. (2012). Beyond crime and drug use: Do adult drug courts produce other psychosocial benefits. *Journal of Drug Issues 42*(2), 156-177.

Harrell, A., Cavanagh, S., & Roman, J. (1998). *Findings from the evaluation of the D.C. Superior Court drug intervention program*.

Harrell, A., Roman, J., & Sack, E. (2001). *Drug court services for female offenders, 1996-1999: Evaluation of the Brooklyn Treatment Court*.

Jones, C. G. A. (2013). Early-phase outcomes from a randomized trial of intensive judicial supervision in an Australian drug court. *Criminal Justice and Behavior*, *40*(4), 453-468.

MacDonald, J. M., Morral, A. R., Raymond, B., & Eibner, C. (2007). The efficacy of the Rio Hondo DUI court: a 2-year field experiment. *Eval Rev*, *31*(1), 4-23. https://doi.org/10.1177/0193841X06287189

NCT02978417. (2016). *Feasibility study of extended-release Naltrexone (Vivitrol) in drug court settings*. https://clinicaltrials.gov/study/NCT02978417

Rodriguez-Monguio, R., Montgomery, B., Drawbridge, D., Packer, I., & Vincent, G. M. (2021). Substance use treatment services utilization and outcomes among probationers in drug courts compared to a matched cohort of probationers in traditional courts. *Am J Addict 30*, 505-513.
