# Supplementary material for: Effectiveness of legally mandated non-custodial drug and alcohol treatment orders for improved health, well-being, global functioning and quality of life: a systematic review and meta-analysis
Source: Health Justice. 2026 Jan 27;14:11. doi: 10.1186/s40352-025-00354-4 (PMC12958499; doi:10.1186/s40352-025-00354-4)
Supplement: Supplementary file 7 — Additional file 7. Participant characteristics. Summary of participants substance use, experience with criminal justice system and clinical factors [file 40352_2025_354_MOESM7_ESM.pdf]

Additional file 7. Summary of participants substance use, experience with criminal justice system and clinical factors

Study (year)	Group	n	Drug or alcohol use	Primary drug of choice	Drug use reported	Alcohol use reported	Duration of drug use	Criminal history	Type of current offense	Type of current sentence	Length of term imposed (months)	Adverse life events	Comorbidities at baseline (ICD-11)	Other concomitant treatments	Interaction with health services reported	Treatment
(Deschenes et al., 1995)	Drug court	176	Use both drugs and alcohol	Mixed	Age at first drug use: 15.7 years; Age at first drug abuse: 24.1 years; % marijuana use/abuse: 51.2; % prior methamphetamine use/abuse: 14.2; % prior cocaine use/abuse: 37.6; % prior crack use/abuse: 2.9; % prior heroin use/abuse: 4.0; % prior other drug use/abuse (e.g. LSD, PCP, Valium, Quaaludes, barbiturates, designer drug (MDMA) and glue sniffing): 6.9	% prior alcohol use/abuse: 84.4	NR	% drug dealer: 17.6; Age at first conviction: 23.2 years; Mean no. of prior arrests: 4.5; Mean no. of prior probation terms: 0.6; Mean no. of prior jail terms: 0.9; % low NIC risk score: 27.8; % medium NIC risk score: 44.3	% possession of narcotics: 26.1; % possession of dangerous drugs: 10.2; % possession of marijuana: 29.5; % possession of drug paraphernalia: 34.1	% probation only: 75.6; % probation and jail or prison: 24.4	35 months	NR	NR	NR	NR	% prior drug treatment: 39.4

Study (year)	Group	n	Drug or alcohol use	Primary drug of choice	Drug use reported	Alcohol use reported	Duration of drug use	Criminal history	Type of current offense	Type of current sentence	Length of term imposed (months)	Adverse life events	Comorbidities at baseline (ICD-11)	Other concomitant treatments	Interaction with health services reported	Treatment
(Deschenes et al., 1995)	Routine probation	454	Use both drugs and alcohol	Mixed	Age at first drug use: 15.5 years; Age at first drug abuse: 23.7 years; % marijuana use/abuse: 59.2; % prior methamphetamine use/abuse: 20.2; % prior cocaine use/abuse: 41.0; % prior crack use/abuse: 5.1; % prior heroin use/abuse: 7.8; % prior other drug use/abuse (e.g. LSD, PCP, Valium, Quaaludes, barbiturates, designer drug (MDMA) and glue sniffing): 7.5	% prior alcohol use/abuse: 81.8	NR	% drug dealer: 19.2; Age at first conviction: 24.2 years; Mean no. of prior arrests: 4.2; Mean no. of prior probation terms: 0.6; Mean no. of prior jail terms: 0.8; % low NIC risk score: 27.8; % medium NIC risk score: 48.5	% possession of narcotics: 20.5; % possession of dangerous drugs: 11.0; % possession of marijuana: 31.9; % possession of drug paraphernalia: 36.6	% probation only: 81.3; % probation and jail or prison: 18.7	34.2 months	NR	NR	NR	NR	% prior drug treatment: 38.2
(Desland & Batey, 1992)	DACAP	47	Use drugs	Heroin	Mean age at first drug use (SD): males: 14.1 (3.6); females: 13.9 (4.1) years; Intensity of heroin use -	NR	Median months since first contact (range): males: 24 (4-	Mean age at first criminal offence (SD): males: 16.8	NR	NR	NR	NR	NR	NR	NR	NR

Study (year)	Group	n	Drug or alcohol use	Primary drug of choice	Drug use reported	Alcohol use reported	Duration of drug use	Criminal history	Type of current offense	Type of current sentence	Length of term imposed (months)	Adverse life events	Comorbidities at baseline (ICD-11)	Other concomitant treatments	Interaction with health services reported	Treatment
					range spent on heroin in last 14 days: males: 0-7800, females: 0-4930		144); females: 38 (8-108)	(3.2); females: 16.8 (3.2) years; Range of number of drug related convictions: males: 1-11; females: 1-4; Range of number of times in jail sentences: males: 0-5; females: 0-2; Range of number of months incarcerated: males: 0-27; females: 0-15								

Study (year)	Group	n	Drug or alcohol use	Primary drug of choice	Drug use reported	Alcohol use reported	Duration of drug use	Criminal history	Type of current offense	Type of current sentence	Length of term imposed (months)	Adverse life events	Comorbidities at baseline (ICD-11)	Other concomitant treatments	Interaction with health services reported	Treatment
(Desland & Batey, 1992)	Self-referred	45	Use drugs	Heroin	Mean age at first drug use (SD): males: 16.4 (4.2); females: 16.2 (4.8) years; Intensity of heroin use - range spent on heroin in last 14 days: males: 0-8420, females: 0-13000	NR	Median months since first contact (range): males: 66 (5-168); females: 68 (6-136)	Mean age at first criminal offence (SD): males: 17.9 (4.3); females: 20.9 (5.2) years; Range of number of drug related convictions: males: 0-5; females: 0-7; Range of number of times in jail sentences: males: 0-3; females: 0-5; Range of number of months incarcer	NR	NR	NR	NR	NR	NR	NR	NR

Study (year)	Group	n	Drug or alcohol use	Primary drug of choice	Drug use reported	Alcohol use reported	Duration of drug use	Criminal history	Type of current offense	Type of current sentence	Length of term imposed (months)	Adverse life events	Comorbidities at baseline (ICD-11)	Other concomitant treatments	Interaction with health services reported	Treatment
								ated: males: 0-21; females: 0-20								
(Festinger et al., 2016)	Computerised HIV prevention intervention	101	NR	NR (“in need of treatment for drug abuse or dependence as assessed by a clinical case manager employed by the court”)	NR	NR	NR	NR	NR (“be charged with a non-violent felony offense, (3) have no more than two prior non-violent convictions, juvenile adjudications or diversionary opportunities”)	NR	NR	NR	NR	NR	NR	NR
(Festinger et al., 2016)	Attention control	99	NR	As above	NR	NR	NR	NR	NR	NR	NR	NR	NR	NR	NR	NR
(Gottfredson & Exum, 2002)	BDTC	139	Use both drugs and alcohol	Mixed	"Data from the ASI …available for only 49 participants: 69% severe	"Data from the ASI …available for only 49	NR	Mean no. prior arrests (SD): 12 (8.8); mean	% participants with at least one: violence	Disposition received: 123 participants;	Sentence length: Assigned days:	NR	NR	NR	NR	"Data from the ASI …available for only 49 participants: 55% prior

Study (year)	Group	n	Drug or alcohol use	Primary drug of choice	Drug use reported	Alcohol use reported	Duration of drug use	Criminal history	Type of current offense	Type of current sentence	Length of term imposed (months)	Adverse life events	Comorbidities at baseline (ICD-11)	Other concomitant treatments	Interaction with health services reported	Treatment
					drug problems..." Of these 87% : heroin as primary or secondary drug of choice; 58% used cocaine; 29% alcohol. 72% reported daily use of crack, cocaine or heroin"	participants: 18% severe alcohol problems"		no. prior convictions (SD): 5.3 (4.3)	or sex charge: 1.4; property charge: 21; drug charge: 71; prostitution or solicitation charge: 5.8; violation of probation charge: 2.2; weapons charge: 0.7; public order charge: 10.9	Sentence received (guilty participants only): % no incarceration and no probation: 2.7; % no incarceration but some probation: 8.9; % incarceration without probation: 10.7; % incarceration with probation: 77.7	1324; Suspended days: 1252; Assigned minus suspended days: 72; Credited days: 38; Remaining days to serve: 35; Probation days: 745					drug treatment.
(Gottfredson & Exum, 2002)	Treatment as usual	96	Use both drugs and alcohol	Mixed	NR	NR	NR	Mean no. prior arrests (SD): 11.3 (7.1); mean no. prior	% participants with at least one: violence or sex charge:	Disposition received: 95 participants; Sentence	Sentence length: Assigned days: 1327; Suspe	NR	NR	NR	NR	NR

Study (year)	Group	n	Drug or alcohol use	Primary drug of choice	Drug use reported	Alcohol use reported	Duration of drug use	Criminal history	Type of current offense	Type of current sentence	Length of term imposed (months)	Adverse life events	Comorbidities at baseline (ICD-11)	Other concomitant treatments	Interaction with health services reported	Treatment
								convictions (SD): 4.6 (3.4)	4.2; property charge: 18.8; drug charge: 71.9; prostitution or solicitation charge: 4.2; violation of probation charge: 4.2; weapons charge: 3.1; public order charge: 4.2	received (guilty participants only): % no incarceration and no probation: 1.3; % no incarceration but some probation: 1.3; % incarceration without probation: 27.6; % incarceration with probation: 69.7	nded days: 1068; Assigned minus supended days: 259; Credited days: 84; Remaining days to serve: 175; Probation days: 613					
(Green & Rempel, 2012)	Drug court	951	Use both drugs and alcohol	Mixed. Primary drug of choice: alcohol (13%); Marijuana/hashish (22%), cocaine (powder)	Drug use in the 6 months prior to baseline (days of use/month): any use of marijuana: 45% (5.87 days); any use of cocaine:	Drug use in the 6 months prior to baseline: any use of heavy alcohol:	Years of drug use (8 drugs): 20.09; Years of drug use (6 drugs, excl.alc	Any prior arrests: 88%; any prior convictions: 72%; any	Criminal activity in 6 months prior to baseline: any criminal activity (74%);	NR	NR	Victimized by any abuse in past year: 42%; Victimized by any	% depressed (10-item inventory): 39%; Anti-social personality disorder (27-item	Any chronic medical problems (currently): 27%	NR	Previous treatment in six months prior to baseline: any drug/alcohol treatment:

Study (year)	Group	n	Drug or alcohol use	Primary drug of choice	Drug use reported	Alcohol use reported	Duration of drug use	Criminal history	Type of current offense	Type of current sentence	Length of term imposed (months)	Adverse life events	Comorbidities at baseline (ICD-11)	Other concomitant treatments	Interaction with health services reported	Treatment
				(11%), crack cocaine (27%), Heroin (5%), Amphetamines (incl. methamphetamines) (9%), Other or claimed not using drugs (13%)	42% (3.84 days); any use of heroin: 11% (1.73); any use of hallucinogens/ designer drugs: 7% (0.19 days); any use of amphetamines: 13% (1.72 days); any use of prescription drugs: illegal use: 16%	41%; days of use/month of heavy alcohol: 3.45	ohol and marijuana): 13.80; Drug use in the 6 months prior to baseline: summary: Any use (8 drugs): 83%; Any use (7 drugs): 72%; Any use (6 drugs): 58%; Days of use per month (8 drugs): 13.16; Days of use per month (7 drugs): 9.5; Days of use per month	prior violent convictions: 15%;	drug activity (69%); mean number of drug-related criminal acts (13.52); % drove while intoxicated (35%)			physical abuse in past year: 30%	inventory): 43%; Narcissistic personality disorder (10-item inventory):48%; Psychotic: anti-social plus narcissistic personality: 27%			29%; days of treatment/ month: 2.02

Study (year)	Group	n	Drug or alcohol use	Primary drug of choice	Drug use reported	Alcohol use reported	Duration of drug use	Criminal history	Type of current offense	Type of current sentence	Length of term imposed (months)	Adverse life events	Comorbidities at baseline (ICD-11)	Other concomitant treatments	Interaction with health services reported	Treatment
							(6 drugs): 7.45									
(Green & Rempel, 2012)	Comparison	523	Use both drugs and alcohol	Mixed. Primary drug of choice: alcohol (14%); Marijuana/hashish (21%), cocaine (powder) (10%), crack cocaine (25%), Heroin (5%), Amphetamines (incl. methamphetamines) (10%), Other or claimed not using drugs (15%)	Drug use in the 6 months prior to baseline (days of use/month): any use of marijuana: 42% (4.68 days); any use of cocaine: 39% (3.91 days); any use of heroin: 12% (1.71); any use of hallucinogens/designer drugs: 7% (0.06 days); any use of amphetamines: 14% (1.63 days); any use of prescription drugs: illegal use: 14%	Drug use in the 6 months prior to baseline: any use of heavy alcohol: 38%; days of use/month of heavy alcohol: 3.15	Years of drug use (8 drugs): 20.49; Years of drug use (6 drugs, excl.alcohol and marijuana): 13.66; Drug use in the 6 months prior to baseline: summary: Any use (8 drugs): 81%; Any use (7 drugs): 67%; Any use (6 drugs): 56%;	Any prior arrests: 91%; any prior convictions: 72%; any prior violent convictions: 14%; Criminal activity in 6 months prior to baseline: any criminal activity (74%); drug activity (67%); mean number of drug-related criminal acts (12.89);	Criminal activity in 6 months prior to baseline: any criminal activity (74%); drug activity (67%); mean number of drug-related criminal acts (12.89); % drove while intoxicated (33%)	NR	NR	Victimized by any abuse in past year: 43%; Victimized by any physical abuse in past year: 32%	% depressed (10-item inventory): 37%; Anti-social personality disorder (27-item inventory): 43%; Narcissistic personality disorder (10-item inventory):50%; Psychotic: anti-social plus narcissistic personality: 26%	Any chronic medical problems (currently): 25%	NR	Previous treatment in six months prior to baseline: any drug/alcohol treatment: 29%; days of treatment/month: 2.15

Study (year)	Group	n	Drug or alcohol use	Primary drug of choice	Drug use reported	Alcohol use reported	Duration of drug use	Criminal history	Type of current offense	Type of current sentence	Length of term imposed (months)	Adverse life events	Comorbidities at baseline (ICD-11)	Other concomitant treatments	Interaction with health services reported	Treatment
							Days of use per month (8 drugs): 12.77; Days of use per month (7 drugs): 8.83; Days of use per month (6 drugs): 7.09	% drove while intoxicated (33%)								
(Harrell et al., 1998)	Sanctions docket	240	Use drugs	NR	Use of strong drugs in first 60 days of case processing (70%); drug use severity in first 60 days of case processing (0.68).	NR	NR	Number of prior arrest in past 5 years: 0.42	NR	NR	NR	NR	NR	NR	NR	NR
(Harrell et al., 1998)	Treatment docket	140	Use drugs	NR	Use of strong drugs in first 60 days of case processing (65%); drug use severity in first 60 days of case processing (0.72).	NR	NR	Number of prior arrest in past 5 years: 0.54	NR	NR	NR	NR	NR	NR	NR	NR

Study (year)	Group	n	Drug or alcohol use	Primary drug of choice	Drug use reported	Alcohol use reported	Duration of drug use	Criminal history	Type of current offense	Type of current sentence	Length of term imposed (months)	Adverse life events	Comorbidities at baseline (ICD-11)	Other concomitant treatments	Interaction with health services reported	Treatment
(Harrell et al., 1998)	Standard docket	311	Use drugs	NR	Use of strong drugs in first 60 days of case processing (73%); drug use severity in first 60 days of case processing (0.75).	NR	NR	Number of prior arrest in past 5 years: 0.43	NR	NR	NR	NR	NR	NR	NR	NR
(Harrell et al., 2001)	Brooklyn Treatment Court	283	Use drugs	NR	Ever used heroin: 87.3%, Ever used cocaine/crack: 72.4%, Any drug use – last 30 days: 55.1%, Used heroin in last 30 days: 27.7%,	NR	NR	Any arrest prior to program entry (official): 24%, Number of prior arrests (official): 3.97 Mean time served in days (self-report): 103.7 days	NR	NR	NR	NR	NR	NR	NR	Number of prior treatment episodes: 0.99
(Harrell et al., 2001)	Comparison	114	Use drugs	NR	Ever used heroin: 62.2%, Ever used cocaine/crack: 78%, Any drug use – last 30 days: 57.9%, Used heroin in last	NR	NR	Any arrest prior to program entry (official): 14%, Number of prior	NR	NR	NR	NR	NR	NR	NR	Number of prior treatment episodes: 0.90

Study (year)	Group	n	Drug or alcohol use	Primary drug of choice	Drug use reported	Alcohol use reported	Duration of drug use	Criminal history	Type of current offense	Type of current sentence	Length of term imposed (months)	Adverse life events	Comorbidities at baseline (ICD-11)	Other concomitant treatments	Interaction with health services reported	Treatment
					30 days: 33.3%,			arrests (official): 3.42 Mean time served in days (self-report): 165.3 days								
(Jones, 2013)	Intensive Judicial Supervision	66	Use both drugs and alcohol	Mixed	Drug being treated (%): Amphetamine (35.4%), Benzodiazepines (29.2%), Cannabis (52.3%), Cocaine (15.4%), Heroin (70.8%)	Drug being treated (%): Alcohol: 15.4%	NR	Most serious offence: break, enter and steal: 37.9%, theft/fraud: 33.3%, driving: 12.1%, Other: 16.7%. Number of court appearances in 5 years prior to index (mean): 5.2. Number of court appearances resulting	Concurrent offenses (median): 5; Initial sentence (mean months): 14.4	NR	NR	NR	NR	NR	NR	Community-based treatment: 84.9%; Pharmacotherapy: 69.7%

Study (year)	Group	n	Drug or alcohol use	Primary drug of choice	Drug use reported	Alcohol use reported	Duration of drug use	Criminal history	Type of current offense	Type of current sentence	Length of term imposed (months)	Adverse life events	Comorbidities at baseline (ICD-11)	Other concomitant treatments	Interaction with health services reported	Treatment
								in prison in 5 years prior to index: none: 21.6%, 1: 30.8%, 2: 23.1%, 3+: 24.6%								
(Jones, 2013)	Supervision as usual	70	Use both drugs and alcohol	Mixed.	Drug being treated (%): Amphetamine (34.8%), Benzodiazepines (23.2%), Cannabis (46.4%), Cocaine (8.7%), Heroin (75.4%)	Drug being treated (%): Alcohol: 7.3%	NR	Most serious offence: break, enter and steal: 25.7%, theft/fraud: 40%, driving: 15.7%, Other: 18.6%. Number of court appearances in 5 years prior to index (mean): 5.1. Number of court appeara	Concurrent offenses (median): 6; Initial sentence (mean months): 15	NR	NR	NR	NR	NR	NR	Community-based treatment: 80.0%; Pharmacotherapy: 67.1%

Study (year)	Group	n	Drug or alcohol use	Primary drug of choice	Drug use reported	Alcohol use reported	Duration of drug use	Criminal history	Type of current offense	Type of current sentence	Length of term imposed (months)	Adverse life events	Comorbidities at baseline (ICD-11)	Other concomitant treatments	Interaction with health services reported	Treatment
								nces resulting in prison in 5 years prior to index: none: 40%, 1: 25.7%, 2: 11.4%, 3+: 22.9%								
(MacDonald et al., 2007)	DUI Court	117	Alcohol	NR	NR	NR	NR	Mean number of prior arrests for DUI: 2.70	NR	NR	NR	NR	NR	Senate Bill 38 program (18- or 30-month treatment and education interventions that include a minimum of 12 hours of educational, 52 hours of group counseling, and biweekly face-	NR	NR

Study (year)	Group	n	Drug or alcohol use	Primary drug of choice	Drug use reported	Alcohol use reported	Duration of drug use	Criminal history	Type of current offense	Type of current sentence	Length of term imposed (months)	Adverse life events	Comorbidities at baseline (ICD-11)	Other concomitant treatments	Interaction with health services reported	Treatment
														to-face check-ins. In addition, they typically offer periodic breath alcohol testing, mandated Alcoholics Anonymous attendance, and anger management or other treatment activities. Individuals who successfully complete the program may have their driver license reinstate		

Study (year)	Group	n	Drug or alcohol use	Primary drug of choice	Drug use reported	Alcohol use reported	Duration of drug use	Criminal history	Type of current offense	Type of current sentence	Length of term imposed (months)	Adverse life events	Comorbidities at baseline (ICD-11)	Other concomitant treatments	Interaction with health services reported	Treatment
														d or reissued		
(MacDonald et al., 2007)	Mandatory minimums	119	Alcohol	NR	NR	NR	NR	Mean number of prior arrests for DUI: 2.55	NR	NR	NR	NR	NR		NR	NR
(NCT02978417, 2016)	Drug Court (Vivitrol)	5	Use drugs	NR	NR	NR	NR	NR	NR	NR	NR	NR	NR	NR	NR	NR
(NCT02978417, 2016)	Treatment as usual	5	Use drugs	NR	NR	NR	NR	NR	NR	NR	NR	NR	NR	NR	NR	NR
(Rodriguez-Monguio et al., 2021)	Drug Courts	271	Use drugs	Opioids primary drug of choice: 84.5%	NR	NR	NR	Median age at first arraignment (SD): 17.29 (4.51), Risk of reoffending at court intake: low (13.7%); low/moderate (5.9%); moderate	NR	NR	NR	NR	NR	NR	NR	Substance use treatment history: 81.5%; missing: 11.8%

Study (year)	Group	n	Drug or alcohol use	Primary drug of choice	Drug use reported	Alcohol use reported	Duration of drug use	Criminal history	Type of current offense	Type of current sentence	Length of term imposed (months)	Adverse life events	Comorbidities at baseline (ICD-11)	Other concomitant treatments	Interaction with health services reported	Treatment
								(28.4%), high (36.9%), very high (5.9%), missing (9.2%)								
(Rodriguez-Monguio et al., 2021)	Traditional Courts	271	Use drugs	Opioids primary drug of choice: 53.5%	NR	NR	NR	Median age at first arraignment (SD): 18.18 (6.21); Risk of reoffending at court intake: low (19.6%); low/moderate (3.3%); moderate (50.2%), high (26.2%), very high (0.4%), missing (0.4%)	NR	NR	NR	NR	NR	NR	NR	Substance use treatment history: 63.1%; missing: 9.6%

References

Deschenes, E. P., Turner, S., & Greenwood, P. W. (1995). Drug court or probation? An experimental evaluation of Maricopa County's drug court. *Justice System Journal 18*(1), 55-73.

Desland, M. L., & Batey, R. G. (1992). A 12-month prospective comparison of court-diverted with self-referred heroin users. *Drug Alcohol Rev*, *11*(2), 121-129. https://doi.org/10.1080/09595239200185591

Festinger, D. S., Dugosh, K. L., Kurth, A. E., & Metzger, D. S. (2016). Examining the efficacy of a computer facilitated HIV prevention tool in drug court. *Drug Alcohol Depend*, *162*, 44-50. https://doi.org/10.1016/j.drugalcdep.2016.02.026

Gottfredson, D. C., & Exum, M. L. (2002). The Baltimore City Drug Treatment Court: One year results from a randomized study. *Journal of Research in Crime and Delinquency 39*(3), 337-356.

Green, M., & Rempel, M. (2012). Beyond crime and drug use: Do adult drug courts produce other psychosocial benefits. *Journal of Drug Issues 42*(2), 156-177.

Harrell, A., Cavanagh, S., & Roman, J. (1998). *Findings from the evaluation of the D.C. Superior Court drug intervention program*.

Harrell, A., Roman, J., & Sack, E. (2001). *Drug court services for female offenders, 1996-1999: Evaluation of the Brooklyn Treatment Court*.

Jones, C. G. A. (2013). Early-phase outcomes from a randomized trial of intensive judicial supervision in an Australian drug court. *Criminal Justice and Behavior*, *40*(4), 453-468.

MacDonald, J. M., Morral, A. R., Raymond, B., & Eibner, C. (2007). The efficacy of the Rio Hondo DUI court: a 2-year field experiment. *Eval Rev*, *31*(1), 4-23. https://doi.org/10.1177/0193841X06287189

NCT02978417. (2016). *Feasibility study of extended-release Naltrexone (Vivitrol) in drug court settings*. https://clinicaltrials.gov/study/NCT02978417

Rodriguez-Monguio, R., Montgomery, B., Drawbridge, D., Packer, I., & Vincent, G. M. (2021). Substance use treatment services utilization and outcomes among probationers in drug courts compared to a matched cohort of probationers in traditional courts. *Am J Addict 30*, 505-513.
