# Supplementary material for: Effectiveness of legally mandated non-custodial drug and alcohol treatment orders for improved health, well-being, global functioning and quality of life: a systematic review and meta-analysis
Source: Health Justice. 2026 Jan 27;14:11. doi: 10.1186/s40352-025-00354-4 (PMC12958499; doi:10.1186/s40352-025-00354-4)
Supplement: Supplementary file 8 — Additional file 8. PROGRESS-plus framework. A summary of participant characteristics mapped to the PROGRESS-plus framework [file 40352_2025_354_MOESM8_ESM.pdf]

Additional file 8. PROGRESS-plus framework (Cochrane Methods Equity, 2021)

Abbreviations: HS: high school; NR: not reported; SD: standard deviation
Notes: * super weighted adjusted for baseline sample differences data presented here

Study (year)	Group name	Group (n)	Age (years)	Sex	Place of residence	Race / ethnicity / culture	Occupation	Religion	Education	Socioeconomic status	Social capital and networks
(Deschenes et al., 1995)	Drug court	176	Age at current conviction: 29.2	% male: 76.1	NR	% African American: 21.6; % Hispanic: 27.8; % White: 48.3	% unemployed at arrest: 46.6	NR	% less than high school education (i.e. no high school diploma): 56.3	See occupation. No other additional measures reported	% Married: 18.2
	Routine probation	454	Age at current conviction: 30.1	% male: 78.6	NR	% African American: 18.7; % Hispanic: 23.3; % White: 55.7	% unemployed at arrest: 41	NR	% less than high school education: 46.7	as above	% Married: 17.2
(Desland & Batey, 1992)	DACAP	47	Male age (SD): 22.9 (6.5); Female age (SD): 21.2 (4.7)	26M/21F	NR	NR	Employment history: % males never employed: 12; % males previously employed: 88; % females never employed: 57; % females previously employed: 43;	NR	Education (range): males 8-10 years; females 7-12 years	NR	Marital status: % single (male): 77, married/de facto: 23, separated: 0; % single (female): 76, married/de facto: 19, separated: 5
	Self-referred	45	Male age (SD): 26.6 (4.2); Female age (SD): 24.8 (4.4)	25M/20F	NR	NR	Employment history: % males never employed: 8; % males previously employed: 92; % females never employed: 20; %	NR	Education (range): males 9-16 years; females 10-16 years	NR	Marital status: % single (male): 44, married/de facto: 48, separated: 8; % single (female): 35, married/de facto: 55, separated: 10

Study (year)	Group name	Group (n)	Age (years)	Sex	Place of residence	Race / ethnicity / culture	Occupation	Religion	Education	Socioeconomic status	Social capital and networks
							females previously employed: 80;				
(Festinger et al., 2016)	Computerised HIV prevention intervention	101	Mean age (SD): 25.37 (7.69)	% male: 81	NR	% African-American: 66%; % Caucasian: 12%; %Not reported/missing:22	NR	NR	NR	NR	Never married: 96%
	Attention control	99	Mean age (SD): 23.77 (6.61)	% male: 82	NR	% African-American: 62; Caucasian: 11%	NR	NR	NR	NR	Never married: 91%
(Gottfredson & Exum, 2002)	BDTC	139	Mean age (SD): 34.8 (7.5)	% male: 74.1	NR	% African-American: 89.2	23 percent were employed	NR	47 percent had at least a high school education	NR	16 percent were married
	Treatment as usual	96	Mean age (SD): 34.7 (7.9)	% male: 74.0	NR	% African-American: 89.6	NR	NR	NR	NR	NR
(Green & Rempel, 2012)*	Drug court	951	33.69	% male: 70.0	Homeless: prior 6 months: 12%	% White: 55, % Black/African American: 32, % Hispanic/Latino: 6, % Other (including multiracial): 7	% currently employed: 37, % currently in school: 8%, number of weeks worked in last 6 months: 8.73	NR	% HS degree/GD or higher: 59	Base 10 log of annual income: $4.46	% primary care responsibility for children < 18 years: 18
	Comparison group	523	34.28	% male: 69.0	Homeless: prior 6 months: 12%	% White: 54, % Black/African American: 25, % Hispanic/Latino: 5, % Other (including multiracial): 6	% currently employed: 37, % currently in school: 9%, number of weeks worked in last 6 months: 8.62	NR	% HS degree/GD or higher: 60	Base 10 log of annual income: $4.46	% primary care responsibility for children < 18 years: 17
(Harrell et al., 1998)	Sanctions docket	240	Median: 33	% male: 86	NR	% African-American: 96	NR	NR	NR	NR	NR
	Treatment docket	140	Median: 29.6	% male: 85	NR	% African-American: 99	NR	NR	NR	NR	NR
	Standard docket	311	Median: 31.2	% male: 85	NR	% African-American: 96	NR	NR	NR	NR	NR

Study (year)	Group name	Group (n)	Age (years)	Sex	Place of residence	Race / ethnicity / culture	Occupation	Religion	Education	Socioeconomic status	Social capital and networks
(Harrell et al., 2001)	Brooklyn Treatment Court	283	35.3	283 F	NR	% African-American: 70%	% employed: 9.9%	NR	NR	NR	Number of children: 2.6; Number of children at home: 0.58
	Comparison group	114	36	114 F	NR	% African-American: 66.7%	% employed: 19.7%	NR	NR	NR	Number of children: 1.9; Number of children at home: 0.54
(Jones, 2013)	Intensive Judicial Supervision	66	Mean: 32.2	% male: 84.9	NR	% Indigenous: 13.6	NR	NR	NR	NR	NR
	Supervision as usual	70	Mean: 32.5	% male: 82.9	NR	% Indigenous: 8.6	NR	NR	NR	NR	NR
(MacDonald et al., 2007)	DUI Court	117	36.6	"92% of all respondents were male"	NR	% Hispanic: 83.7<br><br>% Spanish as primary language:43.2, % Citizen: 40.3	% employed full-time: 76	NR	Education (years of school completed): 10.9	NR	% married: 42.7, Number of children: 2
	Mandatory minimums	119	34.2	as above	NR	% Hispanic: 87.3<br><br>% Spanish as primary language: 46.9, % Citizen: 48.7	% employed full-time: 73.1	NR	Education (years of school completed): 10.8	NR	% married: 38.6, Number of children: 1.8
(NCT02978417, 2016)	Drug Court (Vivitrol)	5	36.2 (9.4)	% male: 80	NR	Ethnicity: % Hispanic or Latino: 0; % NOT Hispanic or Latino: 100; Unknown or not reported: 0. Race: % American Indian or Alaska Native: 0; % Asian: 0; % Native Hawaiian or other Pacific Islander: 0; % Black or African	NR	NR	NR	NR	NR

Study (year)	Group name	Group (n)	Age (years)	Sex	Place of residence	Race / ethnicity / culture	Occupation	Religion	Education	Socioeconomic status	Social capital and networks
						American: 20; % White: 80; % More than one race: 0; % Unknown or not reported: 0					
	Treatment as usual	5	37.4 (13.4)	% male: 60	NR	Ethnicity: % Hispanic or Latino: 20; % NOT Hispanic or Latino: 80; Unknown or not reported: 0; Race: % American Indian or Alaska Native: 0; % Asian: 0; % Native Hawaiian or other Pacific Islander: 0; % Black or African American: 20; % White: 80; % More than one race: 0; % Unknown or not reported: 0	NR	NR	NR	NR	NR
(Rodriguez-Monguio et al., 2021)	Drug Courts	271	Mean age at court intake (SD): 31.5 (8.54)	% male: 66.4	NR	Race: % White: 82.3, % African American: 6.6, % Hispanic: 9.2, % Other: 1.5, % missing: 0.4	% employed full-time: 9.6, % not employed full-time: 84.5, % missing: 5.9	NR	NR	NR	% had children: 59.9%, % missing: 4.4%
	Traditional Courts	271	Mean age at court intake (SD): 35.99 (11.43)	% male: 87.5	NR	Race: % White: 83.4, % African American: 5.5, % Hispanic: 10.3, % Other: 0.7, % missing: 0	% employed full-time: 14.8, % not employed full-time: 84.1, % missing: 1.1	NR	NR	NR	% had children: 50.2%, % missing: 4.1%

References

Cochrane Methods Equity. (2021). *PROGRESS-Plus*. www.methods.cochrane.org/equity/projects/evidence-equity/progress-plus2021.
Deschenes, E. P., Turner, S., & Greenwood, P. W. (1995). Drug court or probation? An experimental evaluation of Maricopa County's drug court. *Justice System Journal 18*(1), 55-73.
Desland, M. L., & Batey, R. G. (1992). A 12-month prospective comparison of court-diverted with self-referred heroin users. *Drug Alcohol Rev*, *11*(2), 121-129. https://doi.org/10.1080/09595239200185591
Festinger, D. S., Dugosh, K. L., Kurth, A. E., & Metzger, D. S. (2016). Examining the efficacy of a computer facilitated HIV prevention tool in drug court. *Drug Alcohol Depend*, *162*, 44-50. https://doi.org/10.1016/j.drugalcdep.2016.02.026
Gottfredson, D. C., & Exum, M. L. (2002). The Baltimore City Drug Treatment Court: One year results from a randomized study. *Journal of Research in Crime and Delinquency 39*(3), 337-356.
Green, M., & Rempel, M. (2012). Beyond crime and drug use: Do adult drug courts produce other psychosocial benefits. *Journal of Drug Issues 42*(2), 156-177.
Harrell, A., Cavanagh, S., & Roman, J. (1998). *Findings from the evaluation of the D.C. Superior Court drug intervention program*.
Harrell, A., Roman, J., & Sack, E. (2001). *Drug court services for female offenders, 1996-1999: Evaluation of the Brooklyn Treatment Court*.
Jones, C. G. A. (2013). Early-phase outcomes from a randomized trial of intensive judicial supervision in an Australian drug court. *Criminal Justice and Behavior*, *40*(4), 453-468.
MacDonald, J. M., Morral, A. R., Raymond, B., & Eibner, C. (2007). The efficacy of the Rio Hondo DUI court: a 2-year field experiment. *Eval Rev*, *31*(1), 4-23. https://doi.org/10.1177/0193841X06287189
NCT02978417. (2016). *Feasibility study of extended-release Naltrexone (Vivitrol) in drug court settings*. https://clinicaltrials.gov/study/NCT02978417
Rodriguez-Monguio, R., Montgomery, B., Drawbridge, D., Packer, I., & Vincent, G. M. (2021). Substance use treatment services utilization and outcomes among probationers in drug courts compared to a matched cohort of probationers in traditional courts. *Am J Addict 30*, 505-513.
