# Supplementary material for: Effectiveness of legally mandated non-custodial drug and alcohol treatment orders for improved health, well-being, global functioning and quality of life: a systematic review and meta-analysis
Source: Health Justice. 2026 Jan 27;14:11. doi: 10.1186/s40352-025-00354-4 (PMC12958499; doi:10.1186/s40352-025-00354-4)
Supplement: Supplementary file 9 — Additional file 9. Interventions profiled using the TIDieR framework. Interventions descriptions delivered mapped to the TIDieR reporting framework [file 40352_2025_354_MOESM9_ESM.pdf]

# Additional file 9. Interventions profiled using the TIDieR framework

Abbreviations: DUI: driving under the influence; FTDO: first time drug offender; NR: not reported; SB 38: Senate Bill 38: are 18- or 30-month treatment and education interventions that include a minimum of 12 hours of educational sessions, 52 hours of group counselling, and biweekly face-to-face check-ins. In addition, they typically offer periodic breath alcohol testing, mandated Alcoholics Anonymous attendance, and anger management or other treatment activities. Individuals who successfully complete the program may have their driver's license reinstated or reissued. TASC: Treatment Alternatives for Safe Communities; TAU: treatment as usual

| Study (year) | Name | Materials | Programme Theory? | Procedures | Provided by | Provider training | Mode | Where | Session | Duration | Tailoring | Modification | Adherence |
|---|---|---|---|---|---|---|---|---|---|---|---|---|---|
| (Deschenes et al., 1995) | Intervention group: FTDO (Track 4) | NR | No | Post adjudication program. Delivered in 3 phases: (a) orientation, (b) stabilisation (focus on preventing relapse) and (c) transition/final. Each phase includes a specialised drug treatment program (drug education class, process group, or twelve-step meeting) plus probation officer contacts (intensive case management and aftercare), and random urine tests detailed in an incentive-based behavioural contract. Points awarded for participation or negative urine tests which can lead to 'rewards' | Private agencies; Probation officers. | NR | Face-to-face; individual and group sessions | Maricopa Court | Phase 1: orientation *Weekly:* 1x education class, 1x process group, at least one 12-step meeting and 1x contact probation officer; and to submit to random urine tests. Phase 2: Stabilisation *Weekly:* 1x process group and 12-step meeting per week and to continue to comply with other terms of probation, including random urine testing. Phase 3: Final/transition | Each phase lasted approx. 2 months (i.e. minimum of 6 months and a maximum of 12 months). | Yes. Behavioural contract. Any phase could be repeated at any time during the FTDO Program | Completion of all three phases of the program within six to twelve months could have their probation terminated or were transferred to standard probation if they had probation conditions, such as community service hours or financial obligations, to complete. | "No problems with implementation of the FTDO assignment-no overrides of drug court assignment were observed" |

| Study (year) | Name | Materials | Programme Theory? | Procedures | Provided by | Provider training | Mode | Where | Session | Duration | Tailoring | Modification | Adherence |
|---|---|---|---|---|---|---|---|---|---|---|---|---|---|
| | | | | e.g. graduate to the next phase. | | | | | As for phase 2. | | | | |
| | Comparator: Routine probation (Tracks 1-3) | NR | | Other three tracks (control group). Tracks 1-3. | NR | NR | NR | NR | NR | NR | NR | NR | |
| (Desland & Batey, 1992) | Intervention: DACAP | NR | Core-shell model: conceptual model to promote diversity in treatment options to meet the needs of the individual | Comprehensive assessment based on three interviews. Information assists sentencing process by providing the court with a profile of the individual and providing the individual with information and access to the most appropriate treatment resource(s). This could include self-control strategies to modify drug use; abstinence orientated services (detoxification, outpatient counselling and therapeutic communities); methadone maintenance treatment. | Treatment agencies, court | NR | NR | Westmead Hospital Drug and Alcohol Unit, Sydney | NR | NR | NR | NR | NR |

| Study (year) | Name | Materials | Programme Theory? | Procedures | Provided by | Provider training | Mode | Where | Session | Duration | Tailoring | Modification | Adherence |
|---|---|---|---|---|---|---|---|---|---|---|---|---|---|
| | Comparator: Self-referred | NR | NA | Self-referred clients to drug court who met the referral criteria | NR | NR | NR | NR | NR | NR | NR | NR | NR |
| (Festinger et al., 2016) | Intervention: Computerised HIV prevention intervention | Condoms, contact card, tablet computer, headphones, gift cards | Intervention based on Computer Assessment and Risk Reduction Education (CARE) Which is based on an overall framework of Information, Motivation, and Behavioural Skills (IMB). Stage-based tailoring of feedback messages and videos follows the Transtheoretical Model | Completed the self-directed CJ-CARE HIV computer-based intervention following each of their first 3 judicial status hearings (approx. 6-, 12- and 18-weeks post program entry). Interventions included “a brief risk assessment, review of identified risks, structured skill building videos, and the development of a risk prevention action plan”. Provided with contact card containing the address of a local free HIV-testing facility and a toll-free number. | Computer-based intervention facilitated by research assistant; clinical case manager | NR | Computer-based | Private rooms outside of the court room to complete their computer sessions | 3 sessions each delivered 6 weeks apart: 20 mins duration for each session | Total regime: 60-90 mins delivered over 3 sessions for 18 weeks. | Yes. “Adaptive nature of the CJ-CARE program, the content of each session was tailored to address the current risks of the participant” | NR | 189 participants (94%) completed all three intervention sessions |
| | Comparator: Attention control | NR | NA | Attention control. Three educational videos focussed on life skills i.e. stress reduction, anger management, and positive listening. Videos did not directly | As above | NR | As above | As above | As above | As above | No | NR | As above |

| Study (year) | Name | Materials | Programme Theory? | Procedures | Provided by | Provider training | Mode | Where | Session | Duration | Tailoring | Modification | Adherence |
|---|---|---|---|---|---|---|---|---|---|---|---|---|---|
| | | | | address HIV or substance abuse. Provided with contact card containing the address of a local free HIV-testing facility and a toll-free number. | | | | | | | | | |
| (Gottfredson & Exum, 2002) | Intervention | NR | Therapeutic jurisprudence in 2002 study but a later linked paper Gottfredson 2007 (Gottfredson et al., 2007) paper refer to Life-course theory; procedural-justice theory | % of participants who received treatment (12 months after study entry): Any treatment: 66.2; certified drug treatment: 48.2, methadone maintenance: 5, Outpatient: 23.7, Residential: 7.2, Correctional: 1.4, Detoxification: 2.2, Intensive outpatient: 19.4, Other treatment: 1.4, Jail-based acupuncture: 39.6. | Community organisations, drug court judges, Division of Parole and Probation | NR | NR | Three distinct sources: circuit court cases supervised by the Division of Parole and Probation, district court cases supervised by the Division of Parole and Probation,and less serious district court cases to be processed by the Alternative Sentencing Unit. | NR | Mean duration of treatment (SD): 125.5 (101.7); Number of treatment episodes: 1.7 | NR | NR | Data on the level of implementation of the various drug court components suggest that the BDTC is implemented unevenly across participants |
| | Comparator: TAU in criminal justice system | NR | NA | TAU in criminal justice system<br>% of participants who received treatment (12 months after study entry): Any treatment: 15.6; certified drug treatment: 13.5 | As above | NR | NR | as above | NR | Mean duration of treatment (SD): 98.9 (97.7); Number of treatment | NR | NR | NR |

| Study (year) | Name | Materials | Programme Theory? | Procedures | Provided by | Provider training | Mode | Where | Session | Duration | Tailoring | Modification | Adherence |
|---|---|---|---|---|---|---|---|---|---|---|---|---|---|
| | | | | methadone maintenance: 7.3, Outpatient: 3.1, Residential: 4.2, Correctional: 0, Detoxification: 0, Intensive outpatient: 2.1, Other treatment: 0, Jail-based acupuncture: 4.2. | | | | | | episodes: 1.3 | | | |
| (Green & Rempel, 2012) | Intervention | NR | The MADCE conceptual framework | Treatments could include hospital-based treatments (e.g. emergency room visits and detoxification), residential treatment, pharmacological treatment (e.g. methadone maintenance), outpatient group and/or individual counselling and self-help groups such as alcoholics anonymous (AA) and narcotics anonymous (NA). Sanctions Included reprimands, warnings, written assignments, community service, increased drug tests, increased attendance at AA/NA meetings, | NR | NR | F2f; 1:1 and group | National Institute of Justice's Multisite Adult Drug Court Evaluation (MADCE). 23 drug court sites located in the following states: Washington (6 sites), Illinois (2 sites), New York (8 sites), Pennsylvania (2 sites), Georgia (2 sites), Florida (2 sites), and South Carolina (1 site). | NR | NR | NR | NR | Due largely to the 8-week maximum window during which baseline interview could be conducted, 28% of technically eligible offenders were not interviewed at baseline. The most common problem was a delay in notification to the research team by staff from the applicable site that an eligible offender had been newly enrolled. |

| Study (year) | Name | Materials | Programme Theory? | Procedures | Provided by | Provider training | Mode | Where | Session | Duration | Tailoring | Modification | Adherence |
|---|---|---|---|---|---|---|---|---|---|---|---|---|---|
| | | | | increased amount or intensity of drug treatment, sitting in the jury box to observe court hearings, being kicked out of court, electronic monitoring, day reporting, house arrest, community control, spending nights in jail, and formal supervision violations | | | | | | | | | |
| | Comparator: treatment as usual | NR | NA | "Rather than a strict no-treatment comparison group, our six comparison sites included a realistic mix of treatment and other counterfactual conditions, similar to what might in fact be found across the country" The North Carolina comparison sites involved standard probation, four sites participation in some variant of a court-mandated treatment initiative: 2 sites | NR | NR | NR | Six sites that lacked adult drug courts or had a narrowly targeted program: one in Washington, one in Illinois, two in Florida, and two in North Carolina. (The one South Carolina drug court, located almost at the North Carolina border, was defined as part of the | NR | NR | NR | More than one third (36%) of the comparison group reported receiving at least some substance-abuse treatment in the first 6 months after baseline, and 71% reported at least some contact with a supervision officer (either a case manager or | NR |

| Study (year) | Name | Materials | Programme Theory? | Procedures | Provided by | Provider training | Mode | Where | Session | Duration | Tailoring | Modification | Adherence |
|---|---|---|---|---|---|---|---|---|---|---|---|---|---|
| | | | | offered TASC programs and two short-term drug-diversion programs that lacked the intensive judicial supervision components of a drug court. | | | | same geographic cluster as the two North Carolina comparison sites.) | | | | probation officer) | |
| (Harrell et al., 1998) | Intervention: Sanctions docket ('graduated sanctions program') | Those joining the sanctions program received written copies of the penalties for failing drug tests. | Conceptual framework | Program of graduated sanctions with weekly drug testing, referrals to community-based treatment, and judicial monitoring of drug use. Penalties increased in severity with each drug test failure Case managers monitored the twice weekly drug tests, notified participants of results, called attorneys to notify them of compliance hearings, and presented reports on client progress at hearings. The judge used this information in selecting the sentence. Enhanced | Judge, one of the four sanctions program case managers, Typical service referrals included community-based drug treatment programs, D.C. Department of Employment, and the Social Security Administration. Each case manager had an average caseload of 25-35 participants, and they shared responsibility for covering | NR | F2f; telephone ("Case managers had weekly telephone contact with defendants who progressed in the program. Participants who continued to test dirty or skip tests had additional face to face meetings with case managers. These defendants were referred by the case manager to an | Court | The program consisted of twice weekly drug testing, judicial monitoring, and consistently applied penalties that increased in severity with additional drug test failures. | NR | Yes. Intervention was tailored based on urinalysis data which was available to the judge | NR | During the experiment, the treatment program shifted from five to three days per week: Mondays, Wednesdays, and Fridays. During that time, the same psycho-education modules were offered but the morning Recovery Group was dropped. Acupuncture also was dropped because the program was unable to pay the acupuncture provider on a regular schedule due to the financial insolvency of the District. Other services |

| Study (year) | Name | Materials | Programme Theory? | Procedures | Provided by | Provider training | Mode | Where | Session | Duration | Tailoring | Modification | Adherence |
|---|---|---|---|---|---|---|---|---|---|---|---|---|---|
| | | | | judicial monitoring. The judge was directly involved in monitoring participants' drug use and oversaw both the legal and the treatment aspects of the case. The judge used the authority of the court to improve treatment outcomes through both positive encouragement when a participant did well and sanctions when the participant did not perform to the program's expectations. Case manager provided information about participation at a city-run treatment program or attendance at NA/AA meetings, if applicable<br><br>Referrals to community-based treatment were provided to a third of the sanctions | court hearings. | | appropriate treatment provider. ) | | | | | | impacted/changed during delivery outlined (pages 78/79) |

| Study (year) | Name | Materials | Programme Theory? | Procedures | Provided by | Provider training | Mode | Where | Session | Duration | Tailoring | Modification | Adherence |
|---|---|---|---|---|---|---|---|---|---|---|---|---|---|
| | | | | program participants. Those referred were more likely to be older (36 years old compared with 31 years old) and, as expected, users of stronger drugs (86% of those referred tested positive for cocaine or heroin in the first two months of case processing, compared with 74% not referred to treatment). Defendants typically spent seven days in the detox unit and were referred to an outpatient treatment program following the intervention | | | | | | | | | |
| | Intervention: Treatment docket | NR | As above | Intensive, court-based day treatment program to drug-involved defendants. Participants were expected to move through sequential treatment stages, which consisted of an Orientation Phase and a five-level Intensive Treatment Phase | NR | NR | F2f | Court | Offered frequent drug testing (daily or 3x week) and an intensive court-based day treatment program. | NR | Yes. Intervention was tailored based on urinalysis data which was available to the judge | NR | NR |

| Study (year) | Name | Materials | Programme Theory? | Procedures | Provided by | Provider training | Mode | Where | Session | Duration | Tailoring | Modification | Adherence |
|---|---|---|---|---|---|---|---|---|---|---|---|---|---|
| | | | | Progression through the program was contingent upon the participant's progress toward the treatment objectives outlined in the treatment plan, academic functioning, participation, and social adjustment. Level movement was designed to reward defendants for positive behaviours and to acknowledge the completion of 21 days of treatment. The treatment team reviewed defendant progress and recommended to the judge whether defendants should move to the next level. Progression to the next level was celebrated in a court ceremony during which the judge congratulated defendants for their success and presented small gifts to recognize | | | | | | | | | |

| Study (year) | Name | Materials | Programme Theory? | Procedures | Provided by | Provider training | Mode | Where | Session | Duration | Tailoring | Modification | Adherence |
|---|---|---|---|---|---|---|---|---|---|---|---|---|---|
| | | | | their achievement of treatment goals. Treatment graduates were honoured with certificates presented by the judge at ceremonies in the courtroom attended by friends, family members, fellow program participants, and staff from the court and treatment program. The program included frequent drug testing (daily or three times per week). The program used the leverage of the court to retain drug-abusing defendants in the treatment program and to provide an accountability structure, thus increasing the chances for improved treatment outcomes. Penalties were imposed by the treatment staff for nonattendance, | | | | | | | | | |

| Study (year) | Name | Materials | Programme Theory? | Procedures | Provided by | Provider training | Mode | Where | Session | Duration | Tailoring | Modification | Adherence |
|---|---|---|---|---|---|---|---|---|---|---|---|---|---|
| | | | | tardiness, and behaviour problems in treatment, but not for positive drug tests. Persistent, serious problems resulted in judicial admonishment and program termination.<br>In addition to the core treatment program, <u>Supplementary services</u> included: individual counselling, acupuncture, and referrals to vocational skills training. Counselling and acupuncture services were offered throughout a participant's program tenure, but participants were encouraged to attend both daily in the orientation phase of treatment.<br><u>Treatment program was designed to:</u> provide the skills, self-esteem, and community resources | | | | | | | | | |

| Study (year) | Name | Materials | Programme Theory? | Procedures | Provided by | Provider training | Mode | Where | Session | Duration | Tailoring | Modification | Adherence |
|---|---|---|---|---|---|---|---|---|---|---|---|---|---|
| | | | | necessary for drug-dependent individuals to leave the drug-using criminal life. Defendants received psycho-educational interventions designed to introduce defendants to central treatment issues. They covered such areas as Substance Abuse Education, Relapse Prevention, Anxiety/Anger Management, Effective Social Communication, and Ethnic Contributions to Civilization. | | | | | | | | | |
| | Comparator: Standard docket | | | Offered drug-involved defendants weekly drug testing, judicial monitoring, and encouragement to seek community-based treatment programs. | Judge | NR | F2f | Court | The standard docket judge saw defendants frequently and interacted directly with them. Defendants were scheduled to come before the judge monthly for status hearings. The judge | NR | Yes. Intervention was tailored based on urinalysis data which was available to the judge | NR | NR |

| Study (year) | Name | Materials | Programme Theory? | Procedures | Provided by | Provider training | Mode | Where | Session | Duration | Tailoring | Modification | Adherence |
|---|---|---|---|---|---|---|---|---|---|---|---|---|---|
| | | | | | | | | | had access to drug test results at the bench to monitor defendant drug use. This information, combined with traditional case processing information, allowed judges to oversee both the legal and the treatment aspects of the defendants' cases. On the standard docket, the number of hearings per defendant ranged from 2 to 24, with an average of 6.8. The cases were open on the docket an average of 273 days, with a median time to disposition of 223 days. | | | | |

| Study (year) | Name | Materials | Programme Theory? | Procedures | Provided by | Provider training | Mode | Where | Session | Duration | Tailoring | Modification | Adherence |
|---|---|---|---|---|---|---|---|---|---|---|---|---|---|
| (Harrell et al., 2001) | Intervention | NR | Drug court model with a conceptual framework for the evaluation of the Brooklyn Criminal Justice Network for female offenders | Primary health services, screening and voluntary testing for tuberculosis, sexually transmitted diseases, pregnancy and HIV, and health education and counselling services, addressing risky sexual behaviour.<br><br>A mental health nurse practitioner was on-site to conduct screening and psychiatric evaluations, deliver services, manage client care (including writing prescriptions) and conduct educational programs and ongoing training for BTC staff.<br>BTC staff also provided a treatment readiness program for clients facing less serious charges, cultural sensitivity workshops, and counselling groups for | Judge, prosecutor, defence attorneys, clinical staff including a mental health nurse practitioner | NR | F2f, 1:1, group | BTC court based services included New York City Department of Health (DOH)<br><br>Primary Health Care clinic located on the first floor of the court building | NR | NR | Yes. "services tailored to the special needs of female offenders in need of drug treatment" | NR | TRI tried a number of strategies for increasing the survey response rate with limited success. These included increasing incentive payments, contacting respondents at their BTC or other court hearings, and field tracing |

| Study (year) | Name | Materials | Programme Theory? | Procedures | Provided by | Provider training | Mode | Where | Session | Duration | Tailoring | Modification | Adherence |
|---|---|---|---|---|---|---|---|---|---|---|---|---|---|
| | | | | domestic violence and parenting skills. BTC also referred clients to off-site health and counselling as needed.<br><br>Incentives and sanctions were used (Level A-C infractions)<br><br>Judicial review | | | | | | | | | |
| | Comparison sample | NR | NR | The comparison sample was composed of 114 women arrested in Brooklyn on eligible drug felony charges during the same period: 66 were arrested in a non-participating prosecution zone; 12 were arrested in the BTC prosecution zone, but not referred to BTC; 46 were referred to BTC but did not enter the court because they never appeared at BTC or were not eligible for other reasons unrelated | NR | NR | NR | NR | NR | NR | NR | NR | NR |

| Study (year) | Name | Materials | Programme Theory? | Procedures | Provided by | Provider training | Mode | Where | Session | Duration | Tailoring | Modification | Adherence |
|---|---|---|---|---|---|---|---|---|---|---|---|---|---|
| | | | | to their drug use (unable to provide documentation of legal residence in the U.S. or case too weak to prosecute).<br><br>Not clear what interventions this group received. | | | | | | | | | |
| (Jones, 2013) | Intervention: Intensive Judicial Supervision | NR | Mentions drug court model | Three phases of treatment before graduation: 1. Initiation; 2. Consolidation, and 3. Reintegration.<br><br>Frequent supervised urine tests and report back to the court regularly during each phase.<br><br>Pharmacotherapy as a treatment for heroin dependence, primarily by way of methadone or buprenorphine maintenance therapy.<br><br>External treatment providers develop individual treatment plans for participants. | Judge, External treatment providers located within local health districts | NR | F2f; 1:1 and group sessions | Parramatta Drug Court | Supervised urine testing is scheduled three times per week during Phase 1 and twice weekly during Phases 2 and 3. Participants are ordinarily required to report back to the drug court once each week during Phase 1, once every 2 weeks during Phase 2, and monthly during Phase 3. | The program is scheduled to run for at least 12 months, but the actual length of time participants spend on the program depends on the length of their initial sentence and their performance on the program. | Yes. Intervention was tailored based on urinalysis data which was available to the judge | NR | See Table 2 - Adherence to Group Assignment Fidelity |

| Study (year) | Name | Materials | Programme Theory? | Procedures | Provided by | Provider training | Mode | Where | Session | Duration | Tailoring | Modification | Adherence |
|---|---|---|---|---|---|---|---|---|---|---|---|---|---|
| | | | | Group sessions, all of which are based on cognitive behavioural principles.<br><br>Flexible system of sanctioning whereby participants can accumulate up to 14 sanction days before having to serve them in custody. Nondisclosed instances of drug use incur more severe sanctions than admitted episodes of use. More serious indiscretions, such as failing to attend counselling sessions or failing to attend court, can result in the imposition of immediate sanctions. Participants can also be rewarded with waived sanctions for sustained periods of abstinence. | | | | | | | | | |
| | Comparator: Supervision as usual | NR | | The SAU condition in the drug court investigated in this study is once per week during Phase 1 (which is | Judge | NR | F2f, 1:1 | Parramatta Drug Court | NR | NR | Yes. Intervention was tailored based on urinanalysis data | NR | See Table 2 - Adherence to Group Assignment Fidelity |

| Study (year) | Name | Materials | Programme Theory? | Procedures | Provided by | Provider training | Mode | Where | Session | Duration | Tailoring | Modification | Adherence |
|---|---|---|---|---|---|---|---|---|---|---|---|---|---|
| | | | | scheduled for 3 months), once every 2 weeks during Phase 2 (which is also scheduled for 3 months), and once every 4 weeks thereafter. Usual judicial supervision levels are therefore much higher than in the courts on which this study | | | | | | | which was available to the judge | | |
| (MacDonald et al., 2007) | Intervention: DUI Court | NR | Mentions drug court model | Overall, the DUI court program was designed to place a greater emphasis on treatment and monitoring than the traditional court procedures. Treatment is closely supervised by the drug court judge, who meets regularly with participants to assess their progress and ongoing needs.<br><br>Incentives/Sanctions:<br>*2nd DUI offence:*<br>Fines/penalties: yes<br>Community service: none<br>Jail: none | Team of judges and court professionals | Experienced in Los Angeles's drug court system | NR | Rio Hondo Municipal Court (El Monte Superior Court), is located in the City of El Monte, in Los Angeles County | NR | NR | NR | NR | NR |

| Study (year) | Name | Materials | Programme Theory? | Procedures | Provided by | Provider training | Mode | Where | Session | Duration | Tailoring | Modification | Adherence |
|---|---|---|---|---|---|---|---|---|---|---|---|---|---|
| | | | | Morgue duty: none<br>Electronic monitoring and breath alcohol testing: 2 weeks<br>Alcohol assessment and recommendations: required<br>Follow-up court appearances: 3 within first 6 months (5-7 total)<br>SB 38: required<br>Do not drink order: required<br>*3rd DUI offence:*<br>Fines/penalties: yes (higher penalty)<br>Community service: none<br>Jail: 30 days<br>Morgue duty: none<br>Electronic monitoring and breath alcohol testing: 110 days<br>3-month treatment: required. Supplemented the SB 38 program with weekly individual counselling and additional group counselling sessions.<br>Alcohol assessment and | | | | | | | | | |

| Study (year) | Name | Materials | Programme Theory? | Procedures | Provided by | Provider training | Mode | Where | Session | Duration | Tailoring | Modification | Adherence |
|---|---|---|---|---|---|---|---|---|---|---|---|---|---|
| | | | | recommendations: not ordered<br>Follow-up court appearances: 2 within first 6 months (8-10 total)<br>SB 38: required<br>Do not drink order: required | | | | | | | | | |
| | Comparator: Mandatory minimums | NR | As above | Incentives/Sanctions:<br>*2nd DUI offence:*<br>Fines/penalties: yes<br>Community service: none<br>Jail: 4 days<br>Morgue duty: none<br>Electronic monitoring and breath alcohol testing: not ordered<br>Alcohol assessment and recommendations: not ordered<br>Follow-up court appearances: 2 within first 6 months (3-4 total)<br>SB 38: required<br>Do not drink order: required<br>*3rd DUI offence:*<br>Fines/penalties: yes (higher penalty)<br>Community service: none<br>Jail: 120 days<br>Morgue duty: none | As above | As above | As above | As above | NR | NR | NR | NR | NR |

| Study (year) | Name | Materials | Programme Theory? | Procedures | Provided by | Provider training | Mode | Where | Session | Duration | Tailoring | Modification | Adherence |
|---|---|---|---|---|---|---|---|---|---|---|---|---|---|
| | | | | Electronic monitoring and breath alcohol testing: not ordered<br>3-month treatment: not ordered<br>Alcohol assessment and recommendations: not ordered<br>Follow-up court appearances: 2 within first 6 months (5-7 total)<br>SB 38: required<br>Do not drink order: required | | | | | | | | | |
| (NCT02978417, 2016) | Intervention | NR | None | Vivitrol® plus treatment as usual (TAU). Fellowship Health Resources (FHR) provide psychosocial treatment such as individual or group therapy, | Fellowship Health Resources, Inc. (FHR), the community behavioural health treatment agency | NR | NR | Wake County Drug Court | NR | NR | NR | NR | NR |
| | Treatment as usual (TAU) | NR | As above | TAU for drug court clients receiving services at FHR includes psychosocial treatment such as individual or group therapy, and sometimes also oral naltrexone for clients who are medically eligible and interested in | As above | NR | NR | As above | NR | NR | NR | NR | NR |

| Study (year) | Name | Materials | Programme Theory? | Procedures | Provided by | Provider training | Mode | Where | Session | Duration | Tailoring | Modification | Adherence |
|---|---|---|---|---|---|---|---|---|---|---|---|---|---|
| | | | | taking the medication, the cost of which is covered by the agency for uninsured clients. Those randomized to TAU would continue with treatment as before, which could include (1) staying on oral naltrexone if already on it, (2) considering starting oral naltrexone, if interested, or (3) continuing with psychosocial treatment only. | | | | | | | | | |
| (Rodriguez-Monguio et al., 2021) | Intervention | NR | None | Not clearly reported. However, treatment analysis suggests that a variety of interventions were included: inpatient, outpatient, short-term and long-term residential, medication-assisted treatment and other treatment services). Inpatient services included hospital stays. Outpatient services included | NR | NR | NR | Drug court in six MA municipalities | NR | NR | NR | NR | Out of 542 probationers, 281 (51.8%) used BSAS treatment services. Of them, 196 (69.8%) were probationers in the drug court system |

| Study (year) | Name | Materials | Programme Theory? | Procedures | Provided by | Provider training | Mode | Where | Session | Duration | Tailoring | Modification | Adherence |
|---|---|---|---|---|---|---|---|---|---|---|---|---|---|
| | | | | counselling, detox, intensive outpatient treatment, and outpatient case management services. The BSAS short-term residential treatment services (residential treatment under 30 days) included medically monitored detoxification services for adults at risk for acute withdrawal from alcohol and other drugs, transitional support and recovery services after detoxification, and case management and referral services for homeless and uninsured adult males in need of substance use treatment services. BSAS long-term residential treatment services (residential treatment over | | | | | | | | | |

| Study (year) | Name | Materials | Programme Theory? | Procedures | Provided by | Provider training | Mode | Where | Session | Duration | Tailoring | Modification | Adherence |
|---|---|---|---|---|---|---|---|---|---|---|---|---|---|
| | Comparator: Traditional court probationers | NR | | As above | NR | NR | NR | Matched cohort of probationers with documented SUD in the traditional court system during the same period in another four municipalities | NR | NR | NR | NR | Out of 542 probationers, 281 (51.8%) used BSAS treatment services. Of them, .. 85 (30.2%) were probationers in the traditional court system |


References

Deschenes, E. P., Turner, S., & Greenwood, P. W. (1995). Drug court or probation? An experimental evaluation of Maricopa County's drug court. *Justice System Journal 18*(1), 55-73.

Desland, M. L., & Batey, R. G. (1992). A 12-month prospective comparison of court-diverted with self-referred heroin users. *Drug Alcohol Rev*, *11*(2), 121-129. https://doi.org/10.1080/09595239200185591

Festinger, D. S., Dugosh, K. L., Kurth, A. E., & Metzger, D. S. (2016). Examining the efficacy of a computer facilitated HIV prevention tool in drug court. *Drug Alcohol Depend*, *162*, 44-50. https://doi.org/10.1016/j.drugalcdep.2016.02.026

Gottfredson, D. C., & Exum, M. L. (2002). The Baltimore City Drug Treatment Court: One year results from a randomized study. *Journal of Research in Crime and Delinquency 39*(3), 337-356.

Gottfredson, D. C., Kearley, B. W., Najaka, S. S., & Rocha, C. M. (2007). How Drug Treatment Courts Work: An Analysis of Mediators. *Journal of Research in Crime and Delinquency*, *44*(1), 3-35. https://doi.org/https://dx.doi.org/10.1177/0022427806291271

Green, M., & Rempel, M. (2012). Beyond crime and drug use: Do adult drug courts produce other psychosocial benefits. *Journal of Drug Issues 42*(2), 156-177.

Harrell, A., Cavanagh, S., & Roman, J. (1998). *Findings from the evaluation of the D.C. Superior Court drug intervention program*.

Harrell, A., Roman, J., & Sack, E. (2001). *Drug court services for female offenders, 1996-1999: Evaluation of the Brooklyn Treatment Court*.

Jones, C. G. A. (2013). Early-phase outcomes from a randomized trial of intensive judicial supervision in an Australian drug court. *Criminal Justice and Behavior*, *40*(4), 453-468.

MacDonald, J. M., Morral, A. R., Raymond, B., & Eibner, C. (2007). The efficacy of the Rio Hondo DUI court: a 2-year field experiment. *Eval Rev*, *31*(1), 4-23. https://doi.org/10.1177/0193841X06287189

NCT02978417. (2016). *Feasibility study of extended-release Naltrexone (Vivitrol) in drug court settings*. https://clinicaltrials.gov/study/NCT02978417

Rodriguez-Monguio, R., Montgomery, B., Drawbridge, D., Packer, I., & Vincent, G. M. (2021). Substance use treatment services utilization and outcomes among probationers in drug courts compared to a matched cohort of probationers in traditional courts. *Am J Addict 30*, 505-513.
