# Supplementary material for: Effectiveness of legally mandated non-custodial drug and alcohol treatment orders for improved health, well-being, global functioning and quality of life: a systematic review and meta-analysis
Source: Health Justice. 2026 Jan 27;14:11. doi: 10.1186/s40352-025-00354-4 (PMC12958499; doi:10.1186/s40352-025-00354-4)
Supplement: Supplementary file 10 — Additional file 10. Relevant outcome measures and data relevant to the review. Summary of outcome measures that were relevant to the review and results as reported by study authors [file 40352_2025_354_MOESM10_ESM.pdf]

Additional file 10. Relevant outcome measures and data relevant to the review

Study (year)	Study design	Outcome / instrument used	Primary outcome or secondary outcome? (only list if trialists have specifically reported it as such)	Mapped to our outcome of interests	Outcome	Collected when?	Intervention Group: Mean (post-intervention) (or other reported result if no mean available) (SD)	Sample size (IG)	Intervention Group 2: Mean (post-intervention) (or other reported result if no mean available) (SD)	Sample size (IG2)	Control Group: Mean (post-intervention) (or other reported result if no mean available) (SD)	Sample size (IG)	Result (as reported by authors)
(Deschenes et al., 1995)	RCT	Objective measures of substance use- urinalysis tests that were conducted at varying frequencies with probationers	Not stated	Secondary_Drug or alcohol use measures reported as: biological alcohol and /or drug use (e.g. measured by testing urine, saliva or analysing hair for drugs, breathalyser for alcohol);		Baseline, 12 months	Data not reported in a way that was suitable for MA (data presented graphically).	NR			Not reported. Data presented graphically.	NR	Almost half of all probationers, both those in drug court and those on routine probation, tested positive for at least one substance during the twelve-month follow-up. A higher proportion of those on routine probation tested positive for cocaine or heroin, while those in drug court were more likely to test positive for marijuana

Study (year)	Study design	Outcome / instrument used	Primary outcome or secondary outcome? (only list if trialists have specifically reported it as such)	Mapped to our outcome of interests	Outcome	Collected when?	Intervention Group: Mean (post-intervention) (or other reported result if no mean available) (SD)	Sample size (IG)	Intervention Group 2: Mean (post-intervention) (or other reported result if no mean available) (SD)	Sample size (IG2)	Control Group: Mean (post-intervention) (or other reported result if no mean available) (SD)	Sample size (IG)	Result (as reported by authors)
(Desland & Batey, 1992)	Not RCT	Heroin use (number of subjects using heroin at each interview)	Not stated	Secondary_Drug or alcohol use measures reported as: biological alcohol and /or drug use (e.g. measured by testing urine, saliva or analysing hair for drugs, breathalyser for alcohol);	Prospective heroin use (number of subjects using heroin at each interview)	Baseline (Interview 1), 2-4 weeks (interview 2), 3 months (interview 3), 6 months (interview 4), 9 months (interview 5), 12 months (interview 6)	Baseline (Interview 1): 41, 2-4 weeks (interview 2):27, 3 months (interview 3): 25, 6 months (interview 4):23 , 9 months (interview 5): 26; 12 months (interview 6): 16	47	NA	NA	Baseline (Interview 1): 41, 2-4 weeks (interview 2):24, 3 months (interview 3): 24, 6 months (interview 4):22 , 9 months (interview 5): 21; 12 months (interview 6): 11	45	

Study (year)	Study design	Outcome / instrument used	Primary outcome or secondary outcome? (only list if trialists have specifically reported it as such)	Mapped to our outcome of interests	Outcome	Collected when?	Intervention Group: Mean (post-intervention) (or other reported result if no mean available) (SD)	Sample size (IG)	Intervention Group 2: Mean (post-intervention) (or other reported result if no mean available) (SD)	Sample size (IG2)	Control Group: Mean (post-intervention) (or other reported result if no mean available) (SD)	Sample size (IG)	Result (as reported by authors)
(Desland & Batey, 1992)	Not RCT	Methadone use (number of participants receiving methadone)	Not stated	Secondary_Drug or alcohol use measures reported as: biological alcohol and /or drug use (e.g. measured by testing urine, saliva or analysing hair for drugs, breathalyser for alcohol);		Baseline (Interview 1), 2-4 weeks (interview 2), 3 months (interview 3), 6 months (interview 4), 9 months (interview 5), 12 months (interview 6)	See Table 4 in Desland 1992. Data reported as number and % for male/female	Varied dependent on time of interview	NA	NA	See Table 4 in Desland 1992. Data reported as number and % for male/female	Varied dependent on time of interview	The self-referred group recorded a higher rate of entry onto methadone maintenance. No sex effect was recorded at any interview. Significant differences were recorded between samples at four interviews (interview 3, P = 0.005; interview 4, P = 0.005; interview 5, P = 0.007; interview 6, P = 0.005). Over time the proportion receiving methadone in the self-referred sample increased (P= 0.000005) while the proportion in the DACAP sample remained constant.

Study (year)	Study design	Outcome / instrument used	Primary outcome or secondary outcome? (only list if trialists have specifically reported it as such)	Mapped to our outcome of interests	Outcome	Collected when?	Intervention Group: Mean (post-intervention) (or other reported result if no mean available) (SD)	Sample size (IG)	Intervention Group 2: Mean (post-intervention) (or other reported result if no mean available) (SD)	Sample size (IG2)	Control Group: Mean (post-intervention) (or other reported result if no mean available) (SD)	Sample size (IG)	Result (as reported by authors)
(Desland & Batey, 1992)	Not RCT	Random urinalysis	Not stated	Secondary_Drug or alcohol use measures reported as: biological alcohol and /or drug use (e.g. measured by testing urine, saliva or analysing hair for drugs, breathalyser for alcohol);	Random urinalysis	NR	NR	NR	NR	NR	NR	NR	Random urinalysis was performed in 52% of all interview. The consistency with self-report data was high at 94%. Regarding biochemical tests, only 55 participants complied

(Green & Rempel, 2012)	Not RCT	Mental health: second item was the result of a 10-question depression inventory, designed to tap feelings and behaviors over the previous week and, after scoring, to yield a dichotomous outcome for whether the offender was depressed	Not stated	Secondary_Depression and anxiety measure	Depression. Next, I will ask you about the ways you may have recently felt or behaved. Please indicate how often you have felt this way during the past week. (Responses: rarely or none of the time, some or a little of the time, occasionally or a moderate amount of the time, all of the time). • I was bothered by things that don't usually bother me. • I had trouble keeping my mind on what I was doing. • I felt depressed. • I felt like everything I did was an effort. • I felt hopeful about the future. • I felt fearful. • My sleep was restless. • I was happy. • I felt lonely. • I could not get going. Respondent is coded as depressed if score is 10 or more. (Each of the 10 questions is coded on a 4-point scale ranging from 0-3.).	Baseline, 6 months, 18 months	26% at 6 months; 27% at 18 months	1009 at 6 months; 951 at 18 months	NA	NA	28% at 6 months; 29% at 18 months	524 at 6 months; 523 at 18 months	Cases were weighted and analyses were performed in HLM 6.04. See forest plot

Study (year)	Study design	Outcome / instrument used	Primary outcome or secondary outcome? (only list if trialists have specifically reported it as such)	Mapped to our outcome of interests	Outcome	Collected when?	Intervention Group: Mean (post-intervention) (or other reported result if no mean available) (SD)	Sample size (IG)	Intervention Group 2: Mean (post-intervention) (or other reported result if no mean available) (SD)	Sample size (IG2)	Control Group: Mean (post-intervention) (or other reported result if no mean available) (SD)	Sample size (IG)	Result (as reported by authors)
Green 2012	Not RCT	Average days of drug use per month	Not stated	Secondary_Drug or alcohol use measures reported as: Self-reported frequency of drug use (e.g. Addiction Severity Index composite scores, timeline follow back method, Alcohol Use Disorders Identification Test (AUDIT))	Predictors of Select Psychosocial Outcomes at 18 Months: Average days of drug use/ month (prior year)	18 months	Employed: Coefficient (SE): −0.029** (0.12); Family conflict (over prior year (SE): 0.030 (0.006)****; Classified as depressed coefficient (SE): 0.154****(0.011)	NR	NA	NA		NR	..parameter estimates displayed toward the bottom of Table 4 appeared to confirm the hypothesized linkage between an offender's drug problems and additional problems in the offender's life. Specifically,across offenders in both the drug court and comparison samples, averaging more days of drug use over the year prior to the 18-month interview significantly predicted a lower likelihood of employment at 18 months, a higher prevalence of family conflict, and a higher probability that the offender was experiencing depression.

Study (year)	Study design	Outcome / instrument used	Primary outcome or secondary outcome? (only list if trialists have specifically reported it as such)	Mapped to our outcome of interests	Outcome	Collected when?	Intervention Group: Mean (post-intervention) (or other reported result if no mean available) (SD)	Sample size (IG)	Intervention Group 2: Mean (post-intervention) (or other reported result if no mean available) (SD)	Sample size (IG2)	Control Group: Mean (post-intervention) (or other reported result if no mean available) (SD)	Sample size (IG)	Result (as reported by authors)
(Harrell et al., 1998)	RCT	Drug use: any drugs	Not stated	Secondary_Drug or alcohol use measures reported as: Self-reported frequency of drug use (e.g. Addiction Severity Index composite scores, timeline follow back method, Alcohol Use Disorders Identification Test (AUDIT))	Drug use after the end of the intervention period is measured by respondent reports of the number and kinds of drugs used during the the twelve months after sentencing	During the the twelve months after sentencing	Data not reported in a way that was analysable.	NA	Data not reported in a way that was analysable.		Data not reported in a way that was analysable.		No significant differences between the sanctions docket eligibles and the standard docket eligibles and no significant differences between the sanctions program participants and the standard docket eligibles after controlling for other variables in the model....no significant reductions in stronger drug use in the year after sentencing among treatment program participants, compared to eligible defendants on the standard docket

(Harrell et al., 2001)	Not RCT	Drug use at follow up (past 30 days)	Not stated	Secondary_Drug or alcohol use measures reported as: Self-reported frequency of drug use (e.g. Addiction Severity Index composite scores, timeline follow back method, Alcohol Use Disorders Identification Test (AUDIT))	Drug use at follow up (past 30 days)	Follow-up questionnaire	Any drug use: 13.8%; Any stronger drug use: 9%; Any marijuana use: 3.7%; Any other drug use: 3.6%; Drinking to intoxication: 5.5%	110	NA	NA	Any drug use: 42.3%; Any stronger drug use: 26.9%; Any marijuana use: 7.7%; Any other drug use: 19.2%; Drinking to intoxication: 23.1%	26	"BTC's impact on drug use was measured by self-reported drug use in the 30 days prior to the follow-up interview. (Note 6) The dependent variables include: 1) use of any drug (yes/no), 2) any stronger drugs (i.e., heroin and/or cocaine use) (yes/no), 3) any marijuana use, and 4) any use of other drugs (including inhalants, hallucinogens and non-narcotic prescription drugs) during the 30 days prior to follow-up. Table 4.1 displays the percentages of clients by group who reported drug use prior to the follow-up interview. (see note 7). These bivariate results, which do not control for sample differences, indicate that the comparison sample members were significantly more likely to report drug use in the 30 days before the follow-up interview in each drug category except marijuana use" Notes: (6) This drug use measure includes use of heroin, other opiates, cocaine, marijuana, amphetamines, barbiturates, other sedatives, hallucinogens, and inhalants. (7) The sample is limited to known drug users and excludes 32 BTC

													sample members put in BTC for urine monitoring because they had been charged with a felony drug offense and subsequently discharged because they did not test positive for drugs while in BTC.

Study (year)	Study design	Outcome / instrument used	Primary outcome or secondary outcome? (only list if trialists have specifically reported it as such)	Mapped to our outcome of interests	Outcome	Collected when?	Intervention Group: Mean (post-intervention) (or other reported result if no mean available) (SD)	Sample size (IG)	Intervention Group 2: Mean (post-intervention) (or other reported result if no mean available) (SD)	Sample size (IG2)	Control Group: Mean (post-intervention) (or other reported result if no mean available) (SD)	Sample size (IG)	Result (as reported by authors)
(Harrell et al., 2001)	Not RCT	Drug use at follow up (past 6 months)	Not stated	Secondary_Drug or alcohol use measures reported as: Self-reported frequency of drug use (e.g. Addiction Severity Index composite scores, timeline follow back method, Alcohol Use Disorders Identification Test (AUDIT))	Drug use at follow up (past 6 months)	Follow-up questionnaire	Any drug use: 33.9%; Any stronger drug use: 25%; Any marijuana use: 7.4%; Any other drug use: 7.3%; Drinking to intoxication: 12.3%	110	NA	NA	Any drug use: 46.2%; Any stronger drug use: 36%; Any marijuana use: 12%; Any other drug use: 16.7%; Drinking to intoxication: 33.3%		the two groups did not differ on the likelihood of reporting drug use in the prior six months (a period during which most BTC sample members were in treatment). Noted a significant difference between groups regarding drinking to intoxication.
(Jones, 2013)	RCT	Drug tests (e.g. urinalysis)	Not stated	Secondary_Drug or alcohol use measures reported as: biological alcohol and /or drug use (e.g. measured by testing urine, saliva or analysing hair for drugs, breathalyser for alcohol);	Odds Ratio (OR) Estimates of Returning a Positive Urinalysis Test at Each Testing Occasion		Data not reported in a way that was analysable.	NA	NA	NA	Data not reported in a way that was analysable.	NA	OR: 0.54, 95% CI [0.34, 0.84], p=0.006; The results show that the odds that participants in the IJS group would return a positive urinalysis test at each testing occasion were 46% lower than those of participants in the SAU group (odds ratio [OR] = 0.54, 95% confidence interval [CI] = [0.34, 0.84], p = .006).

Study (year)	Study design	Outcome / instrument used	Primary outcome or secondary outcome? (only list if trialists have specifically reported it as such)	Mapped to our outcome of interests	Outcome	Collected when?	Intervention Group: Mean (post-intervention) (or other reported result if no mean available) (SD)	Sample size (IG)	Intervention Group 2: Mean (post-intervention) (or other reported result if no mean available) (SD)	Sample size (IG2)	Control Group: Mean (post-intervention) (or other reported result if no mean available) (SD)	Sample size (IG)	Result (as reported by authors)
(MacDonald et al., 2007)	RCT	Number of days drinking 5 or more drinks	Not stated	Secondary_Drug or alcohol use measures reported as: Self-reported frequency of drug use (e.g. Addiction Severity Index composite scores, timeline follow back method, Alcohol Use Disorders Identification Test (AUDIT))	Number of days drinking 5 or more drinks	Baseline, 24 months	Data not reported in a way that was analysable.	NA	NA	NA	Data not reported in a way that was analysable.	NA	Ordinary Least Squares Regression Coefficients and (t-values): All offenders DUI court: 0.74 (0.95), 237 observations, R2=0.01; For example, being assigned to the DUI court did not reduce the number of days (in the prior 30 days) that a participant drank more than five alcoholic beverages (t = 0.95). No differences between the treatment and control groups were observed for rates of binge drinking or alcohol problems at follow-up, nor were there differences in rates of completing the SB 38 program, self-reports of stressful life events, or time spent in jail.

Study (year)	Study design	Outcome / instrument used	Primary outcome or secondary outcome? (only list if trialists have specifically reported it as such)	Mapped to our outcome of interests	Outcome	Collected when?	Intervention Group: Mean (post-intervention) (or other reported result if no mean available) (SD)	Sample size (IG)	Intervention Group 2: Mean (post-intervention) (or other reported result if no mean available) (SD)	Sample size (IG2)	Control Group: Mean (post-intervention) (or other reported result if no mean available) (SD)	Sample size (IG)	Result (as reported by authors)
(MacDonald et al., 2007)	RCT	Self-reported drinking and driving (% past 24 months)	Not stated	Secondary_Drug or alcohol use measures reported as: Self-reported frequency of drug use (e.g. Addiction Severity Index composite scores, timeline follow back method, Alcohol Use Disorders Identification Test (AUDIT))									"assignment to the DUI court was not associated with reductions in either self-reported or official records of drinking and driving. Similarly, the groups did not significantly differ on self-reported drinking behaviors, treatment completion, stressful life events, or jail time. For example, being assigned to the DUI court did not reduce the number of days (in the prior 30 days) that a participant drank more than five alcoholic beverages (t = 0.95)"

Study (year)	Study design	Outcome / instrument used	Primary outcome or secondary outcome? (only list if trialists have specifically reported it as such)	Mapped to our outcome of interests	Outcome	Collected when?	Intervention Group: Mean (post-intervention) (or other reported result if no mean available) (SD)	Sample size (IG)	Intervention Group 2: Mean (post-intervention) (or other reported result if no mean available) (SD)	Sample size (IG2)	Control Group: Mean (post-intervention) (or other reported result if no mean available) (SD)	Sample size (IG)	Result (as reported by authors)
(MacDonald et al., 2007)	RCT	Alcohol Problem Index (past year)	Not stated	Secondary_Severity of dependence: Severity score (e.g. Leeds Dependence Q'nnaire (LDQ), Severity of alcohol dependence, severity of dependence scale (SADQ), Addiction Severity Index composite scores)	Alcohol Problem Index	Baseline, 24 months	Data not reported in a way that was analysable.	NA	NA	NA	Data not reported in a way that was analysable.	NA	Ordinary Least Squares Regression Coefficients and (t-values): All offenders DUI court: 0.07 (0.21), 237 observations, R2=0.05. No differences between the treatment and control groups were observed for rates of binge drinking or alcohol problems at follow-up, nor were there differences in rates of completing the SB 38 program, self-reports of stressful life events, or time spent in jail.
(NCT02978417, 2016)	RCT	Adverse events: All cause mortality	Secondary outcome	Secondary_Adverse events / unintended consequences reported (eg accidental drug overdose, suicide)	All cause mortaility	Approx 6 months	0	5	NA	NA	0	5	No difference in all cause mortality

Study (year)	Study design	Outcome / instrument used	Primary outcome or secondary outcome? (only list if trialists have specifically reported it as such)	Mapped to our outcome of interests	Outcome	Collected when?	Intervention Group: Mean (post-intervention) (or other reported result if no mean available) (SD)	Sample size (IG)	Intervention Group 2: Mean (post-intervention) (or other reported result if no mean available) (SD)	Sample size (IG2)	Control Group: Mean (post-intervention) (or other reported result if no mean available) (SD)	Sample size (IG)	Result (as reported by authors)
(NCT02978417, 2016)	RCT	Other (not including SAE	Secondary outcome	Secondary_Adverse events / unintended consequences reported (eg accidental drug overdose, suicide)	Other (not including SAE - social circumstances i.e. incarceration > 5 days; events were collected by non-systematic assessment)	Approx 6 months	2	5	NA	NA	2	5	NR - results published on clinical trial register website

Study (year)	Study design	Outcome / instrument used	Primary outcome or secondary outcome? (only list if trialists have specifically reported it as such)	Mapped to our outcome of interests	Outcome	Collected when?	Intervention Group: Mean (post-intervention) (or other reported result if no mean available) (SD)	Sample size (IG)	Intervention Group 2: Mean (post-intervention) (or other reported result if no mean available) (SD)	Sample size (IG2)	Control Group: Mean (post-intervention) (or other reported result if no mean available) (SD)	Sample size (IG)	Result (as reported by authors)
(NCT02978417, 2016)	RCT	SAE	Secondary outcome	Secondary_Adverse events / unintended consequences reported (eg accidental drug overdose, suicide)	Serious Adverse events: Injury, poisoning and procedural complications (Drug overdose - unrelated; events were collected by non-systematic assessment)	Approx 6 months	0	5	NA	NA	1	5	NR - results published on clinical trial register website

Study (year)	Study design	Outcome / instrument used	Primary outcome or secondary outcome? (only list if trialists have specifically reported it as such)	Mapped to our outcome of interests	Outcome	Collected when?	Intervention Group: Mean (post-intervention) (or other reported result if no mean available) (SD)	Sample size (IG)	Intervention Group 2: Mean (post-intervention) (or other reported result if no mean available) (SD)	Sample size (IG2)	Control Group: Mean (post-intervention) (or other reported result if no mean available) (SD)	Sample size (IG)	Result (as reported by authors)
NCT02978417	RCT	Number of positive drug screens (Number of times a client had a positive drug screen. This information will be collected from administrative records.)	Secondary outcome	Secondary_Drug or alcohol use measures reported as: biological alcohol and /or drug use (e.g. measured by testing urine, saliva or analysing hair for drugs, breathalyser for alcohol);	Number of positive drug screens	Baseline, 12 months	2 (1.87)	5	NA	NA	2.8 (2.59)	5	NR - results published on clinical trial register website

Study (year)	Study design	Outcome / instrument used	Primary outcome or secondary outcome? (only list if trialists have specifically reported it as such)	Mapped to our outcome of interests	Outcome	Collected when?	Intervention Group: Mean (post-intervention) (or other reported result if no mean available) (SD)	Sample size (IG)	Intervention Group 2: Mean (post-intervention) (or other reported result if no mean available) (SD)	Sample size (IG2)	Control Group: Mean (post-intervention) (or other reported result if no mean available) (SD)	Sample size (IG)	Result (as reported by authors)
NCT02978417	RCT	Number of participants who reported improvement in substance use (Subjective assessment of alcohol/drug use in past 30 days collected via interviews at baseline and approximately six months.)	Secondary outcome	Secondary_Drug or alcohol use measures reported as: Self-reported frequency of drug use (e.g. Addiction Severity Index composite scores, timeline follow back method, Alcohol Use Disorders Identification Test (AUDIT))	Number of participants who reported improvement in substance use	Baseline, 6 months	3 participant	3 participants analysed	NA	NA	1 participant	3 participants analysed	NR - results published on clinical trial register website
(Rodriguez-Monguio et al., 2021)	Not RCT	Relapse (defined as the rate of positive drug tests as a percentage of total drug tests after court intake)	Not stated	Secondary_Drug or alcohol use measures reported as: biological alcohol and /or drug use (e.g. measured by testing urine, saliva or analysing hair for drugs, breathalyser for alcohol);			Adjusted mean estimate: 4.31% ; SD: not reported	143	NA	NA	Adjusted mean estimate: 3.65% ; SD: not reported	101	Difference in the proportion of positive drug tests between probationers in drug courts (4.3% of all drug tests) and traditional courts (3.7%) was not statistically significant (p = .352).

References

Deschenes, E. P., Turner, S., & Greenwood, P. W. (1995). Drug court or probation? An experimental evaluation of Maricopa County's drug court. *Justice System Journal 18*(1), 55-73.

Desland, M. L., & Batey, R. G. (1992). A 12-month prospective comparison of court-diverted with self-referred heroin users. *Drug Alcohol Rev*, *11*(2), 121-129. https://doi.org/10.1080/09595239200185591

Green, M., & Rempel, M. (2012). Beyond crime and drug use: Do adult drug courts produce other psychosocial benefits. *Journal of Drug Issues 42*(2), 156-177.

Harrell, A., Cavanagh, S., & Roman, J. (1998). *Findings from the evaluation of the D.C. Superior Court drug intervention program*.

Harrell, A., Roman, J., & Sack, E. (2001). *Drug court services for female offenders, 1996-1999: Evaluation of the Brooklyn Treatment Court*.

Jones, C. G. A. (2013). Early-phase outcomes from a randomized trial of intensive judicial supervision in an Australian drug court. *Criminal Justice and Behavior*, *40*(4), 453-468.

MacDonald, J. M., Morral, A. R., Raymond, B., & Eibner, C. (2007). The efficacy of the Rio Hondo DUI court: a 2-year field experiment. *Eval Rev*, *31*(1), 4-23. https://doi.org/10.1177/0193841X06287189

NCT02978417. (2016). *Feasibility study of extended-release Naltrexone (Vivitrol) in drug court settings*. https://clinicaltrials.gov/study/NCT02978417

Rodriguez-Monguio, R., Montgomery, B., Drawbridge, D., Packer, I., & Vincent, G. M. (2021). Substance use treatment services utilization and outcomes among probationers in drug courts compared to a matched cohort of probationers in traditional courts. *Am J Addict 30*, 505-513.
