# Supplementary material for: Effectiveness of legally mandated non-custodial drug and alcohol treatment orders for improved health, well-being, global functioning and quality of life: a systematic review and meta-analysis
Source: Health Justice. 2026 Jan 27;14:11. doi: 10.1186/s40352-025-00354-4 (PMC12958499; doi:10.1186/s40352-025-00354-4)
Supplement: Supplementary file 11 — Additional file 11. Outcome measures reported in the included studies that were not relevant to this review. Outcome measures/tools that were used in included studies but that did not meet the pre-defined outcomes of interest for this review [file 40352_2025_354_MOESM11_ESM.pdf]

Additional file 11. Outcome measures reported in the included studies that were not relevant to this review

Study (year)	Outcome / instrument used	Primary outcome or secondary outcome? (as specified by study authors)
(Deschenes et al., 1995)	Involvement in constructive activities (i.e. community service)	Not stated
	Involvement in constructive activities (i.e. court payments)	Not stated
	Drug court status at 12 months (i.e. number of participants who completed drug treatment program or were still in treatment at 12 months)	Not stated
	Involvement in constructive activities (i.e. educational training)	Not stated
	Involvement in constructive activities (i.e. employment)	Not stated
	Number of contacts with probation officers (f2f)	Not stated
	Number of contacts with probation officers (phone)	Not stated
	Recidivism (incarceration for arrests and technical violations)	Not stated
	Recidivism (new arrests and technical violations)	Not stated
	Number of sanctions imposed for positive tests	Not stated
	Counselling and treatment: counselling	Not stated
	Counselling and treatment: in-patient treatment	Not stated
	Counselling and treatment: outpatient	Not stated
(Desland & Batey, 1992)	Numbers employed	Not stated
	Engagement of treatment services (no treatment/self-control strategies; abstinence orientated treatments, methadone maintenance treatments)	Not stated
	Incarceration rates	Not stated
	Enlistment of methadone maintenance services (number of subjects undertaking methadone maintenance at each interview)	Not stated
	Prevalence of HIV	Not stated
	Hepatitis B exposure	Not stated
	Measures of narcotics in social context	Not stated
	Retention rate	Not stated
(Festinger et al., 2016)	Condom procurement	Not stated

Study (year)	Outcome / instrument used	Primary outcome or secondary outcome? (as specified by study authors)
	Engagement in high-risk behaviours using the Risk Assessment Battery tool	Not stated
	HIV testing rates using an HIV testing form	Not stated
(Gottfredson & Exum, 2002)	Interactions with criminal justice system (e.g. Meetings with parole officers, hearings, warrants, technical violations)	Not stated
	Prior offense history (arrests, convictions)	Not stated
	Offence that resulted in inclusion in study	Not stated
	Recidivism (12 months following entry into the program) Included: % rearrested, % reconvicted, Average number of new arrests, Average number of new charges, Average number of new convictions, Percentage of participants with at least one new charge)	Not stated
	Recidivism (arrests, dispositions, sentences, and time incarcerated) through 36 months following entry into the program	Not stated
	Duration of drug treatment	Not stated
	% participants receiving any treatment 12 months after entry into study	Not stated
	% participants receiving certified drug treatment (i.e. methadone maintenance, outpatient, residential, correctional, detoxification, intensive outpatient, other treatment) 12 months after entry into study	Not stated
	% participants receiving jail-based acupuncture 12 months after entry into study	Not stated
	Number of treatment episodes	Not stated
	Duration of treatment	Not stated
(Green & Rempel, 2012)	Criminal behaviour (questions on multiple types of criminal behaviour: drug sales, drug possession, property crime, violent crime, weapons offenses, and public order (e.g., prostitution) offenses. The 6-month data covered the preceding six months, and the 18-month data covered the preceding year)	Not stated
	Socioeconomic well-being: Weeks worked since last interview	Not stated
	Current employment and school enrollment status	Not stated
	Current employment and school enrollment status	Not stated
	Current employment and school enrollment status	Not stated
	Incarceration	Not stated
	Current annual income	Not stated

Study (year)	Outcome / instrument used	Primary outcome or secondary outcome? (as specified by study authors)
	Homelessness and living situation: One question tapped whether the offender had been homeless since the last interview	Not stated
	Homelessness and living situation: second concerned whether, over the same period, the offender had wanted or needed “help with finding or keeping a place to live.”	Not stated
	Mental health: single question that listed five possible response options, “In general, would you say your current emotional or mental health is excellent, very good, good, fair, or poor?”	Not stated
	Mental health: single question that listed five possible response options, “In general, would you say your current emotional or mental health is excellent, very good, good, fair, or poor?”	Not stated
	Official re-arrests	Not stated
	Physical victimisation (Physical victimization covered being pushed, slapped, grabbed, having one’s arm twisted or hair pulled, being restrained or shoved, having something thrown at one that could hurt, being punched or hit with something that could hurt, being kicked, being slammed against a hard surface, beaten up, choked, strangled, burned or scalded on purpose and having a knife or gun used on one)	Not stated
	Family relationships: family conflict (mean response to three statements, each scored on a 5-point Likert-type scale, regarding the degree of family conflict the offender experienced since the previous interview— that is, over the previous 6 months on the 6-month interview and over the previous year on the 18-month interview). Family Conflict. The following statements describe how you may feel about your relationship with your family since your last interview. Please tell me whether you strongly disagree, disagree, neither, agree, or strongly agree with the following statements:<br>• You fight a lot with your family members.<br>• You often feel like you disappoint your family.<br>• You are criticized a lot by your family.	Not stated
	Family relationships: family emotional support (mean response to 10 statements, each on a 5-point scale, regarding the strength of the offender’s relationships with and support from family members since the previous interview). Family Emotional Support. The following statements describe how you may feel about your relationships with your family since your last interview. Please tell me whether you strongly agree, agree, disagree, or strongly disagree with the following statements:<br>• I feel close to my family.<br>• I want my family to be involved in my life.<br>• I consider myself a source of support for my family.	Not stated

Study (year)	Outcome / instrument used	Primary outcome or secondary outcome? (as specified by study authors)
	• I fight a lot with my family members.<br>• I often feel like I disappoint my family.<br>• I am criticized a lot by my family.<br>• I have someone in my family to talk about myself or my problems.<br>• I have someone in my family to turn to for suggestions about how to deal with a personal problem.<br>• I have someone in my family who understood my problems.<br>• I have someone in my family to love me and make me feel wanted.<br>Family Instrumental	
	Family relationships: family instrumental support (Family instrumental support included the mean response to five statements, each on a 5-point scale, regarding expectations since the previous interview that family members would provide tangible assistance if needed, such as a job, finan- cial support, or a place to live). Family Instrumental Support. The following statements describe how you may feel about<br>your relationships with your family since your last interview. Please tell me whether you<br>strongly agree, agree, disagree, or strongly disagree with the following statements:<br>• I have someone in my family who would provide help or advice on finding a place to live.<br>• I have someone in my family who would provide help or advice on finding a job.<br>• I have someone in my family who would provide support for dealing with a substance abuse problem.<br>• I have someone in my family who would provide transportation to work or other<br>appointments if needed.<br>• I have someone in my family who would provide me with financial support	Not stated
	Perceived services wanted/needed: employment services, educational services (e.g., related to GED classes or adult education), financial assistance, or public financial assistance (e.g., related to public disability or welfare).	Not stated
	Perceived services wanted/needed: employment services, educational services (e.g., related to GED classes or adult education), financial assistance, or public financial assistance (e.g., related to public disability or welfare).	Not stated
	Perceived services wanted/needed: employment services, educational services (e.g., related to GED classes or adult education), financial assistance, or public financial assistance (e.g., related to public disability or welfare).	Not stated
	Perceived services wanted/needed: employment services, educational services (e.g., related to GED classes or adult education), financial assistance, or public financial assistance (e.g., related to public disability or welfare).	Not stated

Study (year)	Outcome / instrument used	Primary outcome or secondary outcome? (as specified by study authors)
	Sexual victimization (included being physically forced—by hitting, holding down, or using a weapon—to have oral sex, anal sex, or vaginal sex; having someone verbally insist on sex (oral, anal, or vaginal) when one did not want to; and insisting on sex with- out a condom)	Not stated
(Harrell et al., 1998)	Criminal activity: new arrests (any/number)	Not stated
	Criminal activity: criminal acts	Not stated
	Criminal activity: days to first arrest	Not stated
	Mean Number of Crimes in Year after Sentencing by Treatment Program Participants and Standard Docket Sample: Self-Report Survey Data	Not stated
	Committing Offenses in Year after Sentencing: Self-Report Survey Data	Not stated
	Drug use: types of drugs used	Not stated
	Education/training	Not stated
	Employment status	Not stated
	Income	Not stated
	Drug-related problems	Not stated
	Drug treatment: type of treatment after sentencing	Not stated
	Drug treatment: type of treatment during pretrial release	Not stated
	Drug treatment: type of treatment one year after sentencing	Not stated
(Harrell et al., 2001)	Number of days experiencing employment problems	Not stated
	Number of days experiencing employment problems in the month before follow-up. This variable indicates the number of days the defendant reported experiencing any employment problems in the 30 days before the follow-up interview	Not stated
	Number of days experiencing medical problems	Not stated
	Number of days paid for working in the month before follow-up.	Not stated
	Number of days paid for working in the six months before follow-up. This variable indicates the number of days the defendant reported being paid for working in the 30 days before the follow-up interview	Not stated
	Number of days experiencing medical problems in the month before follow-up. This variable indicates the number of days the defendant reported experiencing any medical problems in the 30 days before the follow-up interview	Not stated

Study (year)	Outcome / instrument used	Primary outcome or secondary outcome? (as specified by study authors)
	Number of days experiencing psychiatric problems in the month before follow-up. This variable indicates the number of days the defendant reported experiencing any psychiatric problems in the 30 days before the follow-up interview.	Not stated
	Any serious psychiatric problems in the month before follow-up. This variable indicates whether the defendant reported experiencing any serious psychiatric problems in the 30 days before the follow-up interview.	Not stated
	Any family problems in the month before follow-up. This variable indicates whether the defendant reported experiencing any serious conflicts with their family in the 30 days before the follow-up interview	Not stated
	Any serious problems in the month before follow-up. This variable indicates whether the defendant reported experiencing any serious conflicts with their family or with other people in the 30 days before the follow-up interview	Not stated
	Any peer problems in the month before follow-up. This variable indicates whether the defendant reported experiencing any serious conflicts with people (excluding family) in the 30 days before the follow up interview.	Not stated
	Amount spent on alcohol in the month before follow-up. This variable indicates the number of days the defendant reported spending on alcohol in the 30 days before the follow-up interview.	Not stated
	Amount spent on drugs in the month before follow-up. This variable indicates the number of days the defendant reported spending on drugs in the 30 days before the follow-up interview	Not stated
	Any arrest after sentencing. This variable indicates whether any arrests occurred in the two years following sample entry	Not stated
	Number of arrests after sentencing. This variable indicates the number of arrests occurring in the two years following sample entry.	Not stated
	Any arrest in the first year after sentencing. This variable indicates whether any arrests occurred in the first year following sample entry	Not stated
	Number of arrests in the first year after sentencing. This variable indicates the number of arrests occurring in the first year following sample entry	Not stated
	Any arrest in the second year after sentencing. This variable indicates whether any arrests occurred in the second year following sample entry.	Not stated
	Number of arrest in the second year after sentencing. This variable indicates the number of arrests occurring in the second year following sample entry.	Not stated

Study (year)	Outcome / instrument used	Primary outcome or secondary outcome? (as specified by study authors)
	Any arrest in the first quarter after sentencing. This variable indicates whether any arrests occurred in the first three months following sample entry	Not stated
	Any arrest in the second quarter after sentencing. This variable indicates whether any arrests occurred in the months four through six following sample entry	Not stated
	Any arrest in the third quarter after sentencing. This variable indicates whether any arrests occurred in months seven through nine following sample entry	Not stated
	Any arrest in the fourth quarter after sentencing. This variable indicates whether any arrests occurred in<br>months ten and twelve following sample entry	Not stated
	Any arrest in the fifth quarter after sentencing. This variable indicates whether any arrests occurred in months thirteen through fifteen following sample entry	Not stated
	Any arrest in the sixth quarter after sentencing. This variable indicates whether any arrests occurred in months sixteen through eighteen following sample entry	Not stated
	Any arrest in the seventh quarter after sentencing. This variable indicates whether any arrests occurred in months nineteen through months twenty-one following sample entry	Not stated
	Any arrest in the eighth quarter after sentencing. This variable indicates whether any arrests occurred in<br>months twenty-two through twenty-four following sample entry.	Not stated
	Any criminal offense in six months prior to follow-up. This variable indicates whether the defendant committed any crimes in the six months prior to follow-up. Crimes included: shoplifting or vandalism; parole or probation violations; drug offenses; forgery; weapons offenses; burglary, larceny, or breaking and entering; robbery; assault; arson; rape; homicide or manslaughter; prostitution; contempt of court; and any other offenses.	Not stated
	Any criminal offense in the 30 days prior to follow-up. This variable indicates whether the defendant committed any crimes in the 30 days prior to follow-up. Crimes included: shoplifting or vandalism; parole<br>or probation violations; drug offenses; forgery; weapons offenses; burglary, larceny, or breaking and<br>entering; robbery; assault; arson; rape; homicide or manslaughter; prostitution; contempt of court; and any other offenses.	Not stated
	Any drug offense in six months prior to follow-up. This variable indicates whether the defendant committed any drug crimes in the six months prior to follow-up.	Not stated
	Any drug offense in the 30 days prior to follow-up. This variable indicates whether the defendant	Not stated

Study (year)	Outcome / instrument used	Primary outcome or secondary outcome? (as specified by study authors)
	committed any crimes in the 30 days prior to follow-up.	
(Jones, 2013)	Progression to phase 2 (occurred for any participant who progressed beyond Phase 1 prior to termination	Not stated
	Termination (i.e. terminated from the program if they were issued with a custodial sentence at the end of their program.)	Not stated
	Number of sanctions (i.e., days in custody) accrued per free week on the program.	Not stated
	Number of sanction days issued to serve during each free week on the program	Not stated
(MacDonald et al., 2007)	Drinking and driving arrests (% past 24 months)	Not stated
	Drinking-related arrest (% past 24 months)	Not stated
	% finished treatment	Not stated
	Jail time (days)	Not stated
	Number of stressful life events (assessed with a 20-item inventory of stressful life events experienced in the past 12 months drawn from the Substance Use Disorder Diagnostic Schedule)	Not stated
(NCT02978417, 2016)	Number of participants with new arrests (Any new arrests during the 12-month study period. This information will be collected from administrative records.)	Primary outcome
	Number of participants who reported illegal behaviour (Subjective assessment of illegal behaviour using the question, "Have you done anything that was against the law in the last 30 days?" collected via interviews approximately six months after baseline.)	Secondary outcome
	Number of participants who reported improvement in subjective functioning: employment/support status (Subjective assessment of employment opportunities collected via interviews at baseline and approximately six months.)	Secondary outcome
	Number of participants with new incarcerations (Any new incarceration during the 12-month study period. This information will be collected from administrative records.)	Primary outcome
	Medical status (Subjective assessment of medical status using the Addiction Severity Index (ASI) Lite (a single measure with multiple domains) collected via interviews at baseline and approximately six months.)	Secondary outcome
	Psychiatric status (Subjective assessment of psychiatric status using the Addiction Severity Index (ASI) Lite (a single measure with multiple domains) collected via interviews at baseline and approximately six months.)	Secondary outcome
	Number of missed court appointments (Number of missed court appointments during the 12-month study period. This information will be collected from administrative records.)	Secondary outcome

Study (year)	Outcome / instrument used	Primary outcome or secondary outcome? (as specified by study authors)
	Number of participants who reported improvement in subjective functioning (Family/social relationships) Subjective assessment of family and social relationships using a single question about satisfaction with relationships over the past 30 days collected via interviews at baseline and approximately six months.	Secondary outcome
	Number of sanctions imposed by the court (Number of sanctions imposed by the court (e.g., brief stays in jail). This information will be collected from administrative records.)	Secondary outcome
	Treatment participation (non-vivitrol)	Secondary outcome
	Treatment satisfaction score (Reported as average of 12-item treatment satisfaction score at approximately six months. A score of 5 indicates strong agreement with positive statements about treatment satisfaction; a score of 0 indicates strong disagreement.)	
	Change in medication assisted treatment attitudes (Change in MAT attitudes from baseline to follow-up. The average score of 4 questions about MAT for substance use disorder were calculated at baseline and follow-up, and the average score at baseline was subtracted from the average score at follow-up. The averages are based on questions for which a score of 5 indicates strong agreement with statements about MAT; a score of 1 indicates strong disagreement with such statements)	
	Vivitrol participation (Number of Vivitrol injections from either arm of study. This information will be collected from administrative records.)	Secondary outcome
(Rodriguez-Monguio et al., 2021)	Substance use-related recidivism (defined as drug possession, distribution, trafficking, and driving while intoxicated)	Not stated
	Recidivism rates (any new arraignment or new conviction after court intake)	Not stated
	Severity of recidivism (classified as a nonviolent/nonserious offense, including possession, operating after a revoked or suspended license, prostitution, attempted crimes, and petty crimes (e.g., vandalism); a nonviolent/serious offense, including trafficking, distribution, breaking and entering, operating under the influence, abuse prevention act violations, arson, and larceny and fraud; and a violent offense, including any offenses that could result in harm to an individual, such as assault, rape, carjacking, or robbery)	Not stated
	Risk of recidivism (ascertained by the Ohio Risk Assessment Community Supervision Tool (ORAS-CST) or by the Ohio Risk Assessment Community Supervision Screening Tool (ORAS- CSST) for probationers who received only the screening tool and scored low risk, in which case they did not receive the full assessment (i.e., ORAS-CST).10 Where the ORAS data were not available (n = 25 probationers), the risk level was ascertained by the	Not stated

Study (year)	Outcome / instrument used	Primary outcome or secondary outcome? (as specified by study authors)
	Level of Service: Risk, Need, and Responsivity, Risk and Needs Triage (RANT), or age at first arraignment as a proxy for risk—age at first arraignment was a significant predictor of recidivism)	
	Substance use-related new arraignments	Not stated
	Substance use-related new convictions	Not stated
	Bureau of Substance Addiction Services (BSAS) treatment services utilization (number of service units provided to probationers for each service type)	Not stated
	BSAS expenditures (BSAS payments for each corresponding service)	Not stated
	BSAS treatment service category (i.e., inpatient, outpatient, short-term and long-term residential, medication-assisted treatment [MAT], and other treatment services)	Not stated

References

Deschenes, E. P., Turner, S., & Greenwood, P. W. (1995). Drug court or probation? An experimental evaluation of Maricopa County's drug court. *Justice System Journal 18*(1), 55-73.

Desland, M. L., & Batey, R. G. (1992). A 12-month prospective comparison of court-diverted with self-referred heroin users. *Drug Alcohol Rev*, *11*(2), 121-129. https://doi.org/10.1080/09595239200185591

Festinger, D. S., Dugosh, K. L., Kurth, A. E., & Metzger, D. S. (2016). Examining the efficacy of a computer facilitated HIV prevention tool in drug court. *Drug Alcohol Depend*, *162*, 44-50. https://doi.org/10.1016/j.drugalcdep.2016.02.026

Gottfredson, D. C., & Exum, M. L. (2002). The Baltimore City Drug Treatment Court: One year results from a randomized study. *Journal of Research in Crime and Delinquency 39*(3), 337-356.

Green, M., & Rempel, M. (2012). Beyond crime and drug use: Do adult drug courts produce other psychosocial benefits. *Journal of Drug Issues 42*(2), 156-177.

Harrell, A., Cavanagh, S., & Roman, J. (1998). *Findings from the evaluation of the D.C. Superior Court drug intervention program*.

Harrell, A., Roman, J., & Sack, E. (2001). *Drug court services for female offenders, 1996-1999: Evaluation of the Brooklyn Treatment Court*.

Jones, C. G. A. (2013). Early-phase outcomes from a randomized trial of intensive judicial supervision in an Australian drug court. *Criminal Justice and Behavior*, *40*(4), 453-468.

MacDonald, J. M., Morral, A. R., Raymond, B., & Eibner, C. (2007). The efficacy of the Rio Hondo DUI court: a 2-year field experiment. *Eval Rev*, *31*(1), 4-23. https://doi.org/10.1177/0193841X06287189

NCT02978417. (2016). *Feasibility study of extended-release Naltrexone (Vivitrol) in drug court settings*. https://clinicaltrials.gov/study/NCT02978417

Rodriguez-Monguio, R., Montgomery, B., Drawbridge, D., Packer, I., & Vincent, G. M. (2021). Substance use treatment services utilization and outcomes among probationers

in drug courts compared to a matched cohort of probationers in traditional courts. *Am J Addict 30*, 505-513.
