# Supplementary material for: Effectiveness of legally mandated non-custodial drug and alcohol treatment orders for improved health, well-being, global functioning and quality of life: a systematic review and meta-analysis
Source: Health Justice. 2026 Jan 27;14:11. doi: 10.1186/s40352-025-00354-4 (PMC12958499; doi:10.1186/s40352-025-00354-4)
Supplement: Supplementary file 12 — Additional file 12. ROB judgements. Consensus decisions for risk of bias judgement across nine EPOC domains [file 40352_2025_354_MOESM12_ESM.pdf]

Additional file 12. ROB judgements

Study ID	Random sequence generation (selection bias)	Random sequence generation (selection bias) supporting text	Allocation concealment (selection bias)	Allocation concealment (selection bias) supporting text	Baseline outcome measurements (similar)	Baseline outcome measurements (similar) supporting text	Baseline characteristics (comparable)	Baseline characteristics (comparable) supporting text	Knowledge of the allocated interventions adequately prevented during the study	Knowledge of the allocated interventions adequately prevented during the study supporting text	Protection against contamination	Protection against contamination supporting text	Incomplete outcome data (attrition bias)	Incomplete outcome data (attrition bias) supporting text	Selective outcome reporting (reporting bias)	Selective outcome reporting (reporting bias) supporting text	Other risks of bias	Other risks of bias supporting text
(Deschenes et al., 1995)	Low	"all individuals identified as eligible for the FTDO Program were randomly assigned to the study groups as they appeared for their probation assignment. The clerk at the assignment desk used a computer-generated program designed by RAND to assign the individuals to one of the four tracks and would inform the probationer to report to the assigned probation officer.16 The random assignment	Low	"clerk at the assignment desk used a computer-generated program designed by RAND to assign the individuals to one of the four tracks and would inform the probationer to report to the assigned probation officer" (Discuss because there is potential for bias here)	Unclear	Baseline outcome measures are not clearly reported and data appears to be post-intervention data only. Also query whether there are any actual health outcomes reported here. "Outcome measures include drug use, involvement in drug treatment, drug test results, rearrest record, drug-related criminal activity, employment and other socially productive behavior, and justice system costs"	Low	Baseline characteristics in Table 1 appear comparable. ("no significant differences between those probationers randomly assigned to standard probation and those assigned to drug court ")	Unclear	Insufficient information ("Outcome data were gathered from probation files, which include the probation officer's reports of the individual's behavior while on probation, the progress reports from the treatment programs in which the offenders participate, and the results of urinalysis tests and the action taken in response to positive tests. Data on technical	Low	Unlikely that the control group received the intervention but limited information reported	Low	Only 9 out of 639 had missing data at 12-month follow-up	Unclear	Insufficient information. Study was not registered and there does not appear to be any protocol but the outcomes that were reported are presented but the data presented is limited ("The primary measures we used for program implementation were the number and type of services received and the nature of the case management for both experimental and control	High	It is unclear what intervention the control group received ("this article restricts the comparison to the drug court participants and the combined average of the three probation tracks. The latter result may reflect differential testing among conditions. As part of the evaluation of the

Study ID	Random sequence generation (selection bias)	Random sequence generation (selection bias) supporting text	Allocation concealment (selection bias)	Allocation concealment (selection bias) supporting text	Baseline outcome measurements (similar)	Baseline outcome measurements (similar) supporting text	Baseline characteristics (comparable)	Baseline characteristics (comparable) supporting text	Knowledge of the allocated interventions adequately prevented during the study	Knowledge of the allocated interventions adequately prevented during the study supporting text	Protection against contamination	Protection against contamination supporting text	Incomplete outcome data (attrition bias)	Incomplete outcome data (attrition bias) supporting text	Selective outcome reporting (reporting bias)	Selective outcome reporting (reporting bias) supporting text	Other risks of bias	Other risks of bias supporting text
		of probationers to the FTDO Program began in March of 1992 and continued until April of 1993, resulting in 639 persons being assigned to the evaluation"								violations, new arrests, and sanctions were recorded from both the probation files and the LEJIS system")						offenders during their follow-up. Specifically, the number and type of face-to-face and phone contacts with clients, the extent and nature of monitoring checks performed (e.g., criminal record checks, employment verification), and the number of drug and alcohol tests requested by the probation officer were recorded from probation files. Information about the dates of the court review hearings		FTDO Program, the two tracks with drug testing (tracks 2 and 3) and drug court participants were to have marijuana testing at least once a month. Probation's usual practice is not to test for marijuana. Although the drug court participants were routinely tested for marijuana, we have insufficient information

Study ID	Random sequence generation (selection bias)	Random sequence generation (selection bias) supporting text	Allocation concealment (selection bias)	Allocation concealment (selection bias) supporting text	Baseline outcome measurements (similar)	Baseline outcome measurements (similar) supporting text	Baseline characteristics (comparable)	Baseline characteristics (comparable) supporting text	Knowledge of the allocated interventions adequately prevented during the study	Knowledge of the allocated interventions adequately prevented during the study supporting text	Protection against contamination	Protection against contamination supporting text	Incomplete outcome data (attrition bias)	Incomplete outcome data (attrition bias) supporting text	Selective outcome reporting (reporting bias)	Selective outcome reporting (reporting bias) supporting text	Other risks of bias	Other risks of bias supporting text
																and the judicial decisions were coded from the reports in the probation files using RAND's six- and twelve-month review forms. 20 Reports from the private treatment provider contained in the probation files were used to record referrals to treatment, the duration of treatment, and the types of counseling and education provided. In addition, data on drug court appearances and progress during		about whether tracks 2 and 3 actually included marijuana in their drug screen panels")

Study ID	Random sequence generation (selection bias)	Random sequence generation (selection bias) supporting text	Allocation concealment (selection bias)	Allocation concealment (selection bias) supporting text	Baseline outcome measurements (similar)	Baseline outcome measurements (similar) supporting text	Baseline characteristics (comparable)	Baseline characteristics (comparable) supporting text	Knowledge of the allocated interventions adequately prevented during the study	Knowledge of the allocated interventions adequately prevented during the study supporting text	Protection against contamination	Protection against contamination supporting text	Incomplete outcome data (attrition bias)	Incomplete outcome data (attrition bias) supporting text	Selective outcome reporting (reporting bias)	Selective outcome reporting (reporting bias) supporting text	Other risks of bias	Other risks of bias supporting text
																treatment were recorded on a drug court review form.21 Outcome measures include drug use, involvement in drug treatment, drug test results, rearrest record, drug-related criminal activity, employment and other socially productive behavior, and justice system costs. ")		
(Desland & Batey, 1992)	High	Consecutively entered into the study if meeting referral criteria	High	Groups indicated whether you were self-referred or DACAP, so no concealment	High	Significant differences on some factors detected at baseline, and although discussed, no adjustment to analysis was made	High	Baseline groups not comparable "DACAP referrals were significantly younger (P = o.oo2), tended to be single (P = o.oo2),	High		Unclear	Insufficient information	Low	Follow-up interviews averaged 76% of the original sample with 83% of the original sample being intervie	Low		High	Rarely did subjects report completing agreed treatment contracts

Study ID	Random sequence generation (selection bias)	Random sequence generation (selection bias) supporting text	Allocation concealment (selection bias)	Allocation concealment (selection bias) supporting text	Baseline outcome measurements (similar)	Baseline outcome measurements (similar) supporting text	Baseline characteristics (comparable)	Baseline characteristics (comparable) supporting text	Knowledge of the allocated interventions adequately prevented during the study	Knowledge of the allocated interventions adequately prevented during the study supporting text	Protection against contamination	Protection against contamination supporting text	Incomplete outcome data (attrition bias)	Incomplete outcome data (attrition bias) supporting text	Selective outcome reporting (reporting bias)	Selective outcome reporting (reporting bias) supporting text	Other risks of bias	Other risks of bias supporting text
								less educated (P = o.ooi) (this was most apparent in females in both referral groups, P= o.oo6), displayed a poorer employment history (P = o.o3) (again most apparent in the DACAP females compared to all other groups, P = o.oo2), and commenced illicit drug use and criminal activity at an earlier age (both P= o.oi). Comparison of heroin use indicated the DACAP sample to be using less						wed at i2 months.. .A detailed report of the methodology and retention rates are available elsewhere				

Study ID	Random sequence generation (selection bias)	Random sequence generation (selection bias) supporting text	Allocation concealment (selection bias)	Allocation concealment (selection bias) supporting text	Baseline outcome measurements (similar)	Baseline outcome measurements (similar) supporting text	Baseline characteristics (comparable)	Baseline characteristics (comparable) supporting text	Knowledge of the allocated interventions adequately prevented during the study	Knowledge of the allocated interventions adequately prevented during the study supporting text	Protection against contamination	Protection against contamination supporting text	Incomplete outcome data (attrition bias)	Incomplete outcome data (attrition bias) supporting text	Selective outcome reporting (reporting bias)	Selective outcome reporting (reporting bias) supporting text	Other risks of bias	Other risks of bias supporting text
								intensively at presentation (P = o.oo6) with a shorter history of use (P = o.o3). Concerning other drug and alcohol use, no significant differences were detected.										
(Festinger et al., 2016)	Unclear	Insufficient information ("randomly assigned")	Unclear	Insufficient information ("randomly assigned")	Low	Baseline outcomes appear comparable	Low	"Participants in the two groups did not differ on any baseline item"	Unclear	Insufficient information.	Low	Conducted at same drug court however, intervention is a computerised program, so unlikely control would have "accidentally" received the intervention	Low	Dropouts clearly reported but there appears to be some confusion in the CONSORT figure btw numbers in exptl group compared with numbers in control. Contact authors.	Low	Unable to locate a clinical trial record, however all outcomes appear to be reported fully	Low	No other concerns
(Gottfredson & Exum, 2002)	High	Process of randomisation not discussed. However, after	High	Not blinded as allocation results shared	Low		Low	No significant differences and no meaningful	Low	objective outcome measures	High	The randomization results were given to the	High	Dropouts over time (see Table)	Low		Low	

Study ID	Random sequence generation (selection bias)	Random sequence generation (selection bias) supporting text	Allocation concealment (selection bias)	Allocation concealment (selection bias) supporting text	Baseline outcome measurements (similar)	Baseline outcome measurements (similar) supporting text	Baseline characteristics (comparable)	Baseline characteristics (comparable) supporting text	Knowledge of the allocated interventions adequately prevented during the study	Knowledge of the allocated interventions adequately prevented during the study supporting text	Protection against contamination	Protection against contamination supporting text	Incomplete outcome data (attrition bias)	Incomplete outcome data (attrition bias) supporting text	Selective outcome reporting (reporting bias)	Selective outcome reporting (reporting bias) supporting text	Other risks of bias	Other risks of bias supporting text
		randomisation, judge had the power to overrule the allocation to treatment/control although the allocation was "followed in most cases"		with judge				differences between the experimental and control groups were found on the following variables: percentage African American; percentage male; age at initial arrest; number of prior arrests; number of prior convictions; or percentage whose initial arrests included violent, property, drug, sex, violation of probation, or other charges. Therefore, the groups appear similar.				judge as a recommendation and were followed in most cases (because the judges had agreed to participate in the study). Also possible that treatment and control delivered at same drug courts but the same personnel						

Study ID	Random sequence generation (selection bias)	Random sequence generation (selection bias) supporting text	Allocation concealment (selection bias)	Allocation concealment (selection bias) supporting text	Baseline outcome measurements (similar)	Baseline outcome measurements (similar) supporting text	Baseline characteristics (comparable)	Baseline characteristics (comparable) supporting text	Knowledge of the allocated interventions adequately prevented during the study	Knowledge of the allocated interventions adequately prevented during the study supporting text	Protection against contamination	Protection against contamination supporting text	Incomplete outcome data (attrition bias)	Incomplete outcome data (attrition bias) supporting text	Selective outcome reporting (reporting bias)	Selective outcome reporting (reporting bias) supporting text	Other risks of bias	Other risks of bias supporting text
(Green & Rempel, 2012)	High	CBA design with propensity scoring	High	CBA design with propensity scoring	Low	"Implemented a standard propensity score adjustment"	Low	Weighted analysis applied to the baseline characteristics	High		High	Almost a third of the comparison group received some additional intervention	Low	No differential ("Among those interviewed at baseline, follow-up interviews were successfully conducted with 1,533 offenders at 6 months (87% of drug court and 84% of comparison), 1,474 offenders at 18 months (82% of drug court and 84% of comparison), and 1,349 offenders at both follow-up periods (an identical 76% of	Unclear	Insufficient information.	Low	

Study ID	Random sequence generation (selection bias)	Random sequence generation (selection bias) supporting text	Allocation concealment (selection bias)	Allocation concealment (selection bias) supporting text	Baseline outcome measurements (similar)	Baseline outcome measurements (similar) supporting text	Baseline characteristics (comparable)	Baseline characteristics (comparable) supporting text	Knowledge of the allocated interventions adequately prevented during the study	Knowledge of the allocated interventions adequately prevented during the study supporting text	Protection against contamination	Protection against contamination supporting text	Incomplete outcome data (attrition bias)	Incomplete outcome data (attrition bias) supporting text	Selective outcome reporting (reporting bias)	Selective outcome reporting (reporting bias) supporting text	Other risks of bias	Other risks of bias supporting text
														both samples) ") Reasons for withdrawal were not documented				
(Harrell et al., 1998)	Unclear	Randomly assigned, but no description of the process.	Unclear	Not reported	Unclear	although statistical procedures, described in earlier chapters, are used to adjust for potential bias, these adjustments may not fully control for self-selection into treatment.	Low	However, program participants differed significantly in several ways from those eligibles who did not join. Measures implemented to account for this (chapter 3)	Low	Gallup project staff and interviewers were blind to the respondents,Äô docket assignment.	Unclear	Not reported	Low	Procedures used to analyze and adjust for nonresponse bias	Low	Procedures used to analyze and adjust for nonresponse bias (Chapter 3)	Low	Chapter 3 discusses measures taken to ensure experiment validity.
(Harrell et al., 2001)	High	No random sampling	High	No blinding	High	Some significant differences were found between the groups at baseline.	Low	Reporting similar	High	Different courts so knowledge of which group received intervention and which the control.	Low	Different courts implemented control and intervention.	High	In all, only about one-third of those included in the baseline sample were interviewed at follow-up	Unclear	Problems in those wanting to be interviewed and attrition rates led to "major changes in the evaluation design and work plan"	Unclear	

Study ID	Random sequence generation (selection bias)	Random sequence generation (selection bias) supporting text	Allocation concealment (selection bias)	Allocation concealment (selection bias) supporting text	Baseline outcome measurements (similar)	Baseline outcome measurements (similar) supporting text	Baseline characteristics (comparable)	Baseline characteristics (comparable) supporting text	Knowledge of the allocated interventions adequately prevented during the study	Knowledge of the allocated interventions adequately prevented during the study supporting text	Protection against contamination	Protection against contamination supporting text	Incomplete outcome data (attrition bias)	Incomplete outcome data (attrition bias) supporting text	Selective outcome reporting (reporting bias)	Selective outcome reporting (reporting bias) supporting text	Other risks of bias	Other risks of bias supporting text
(Jones, 2013)	Low	Each participant was allocated into the IJS or SAU group according to a randomized schedule. Participants were randomized within blocks of 8 to ensure a balanced accrual into the respective conditions over time. All court officers were blinded to the randomization mechanism	Unclear	Insufficient information	Unclear	Insufficient data reported to make this judgement	Low	There were no statistically significant differences between IJS and SAU participants on any of these characteristics	High	nonblinded because neither the judicial officer nor the participants could be blinded to the condition to which they had been allocated.	Low	Only 1 participant was deliberately assigned to the incorrect condition (an SAU participant who was supervised twice per week), and the remaining apparent contamination across the two groups was attributed to the fact that some participants spent only a short time on the program before being terminated.	High	Although 160 participants were initially randomized into the study, 24 were subsequently withdrawn (n = 14 IJS and n = 10 SAU participants). Three participants (n = 2 IJS; n = 1 SAU) were found to be ineligible after they had been held over for sentence and were excluded from participation in the program. No outcome data were	Low		Low	

Study ID	Random sequence generation (selection bias)	Random sequence generation (selection bias) supporting text	Allocation concealment (selection bias)	Allocation concealment (selection bias) supporting text	Baseline outcome measurements (similar)	Baseline outcome measurements (similar) supporting text	Baseline characteristics (comparable)	Baseline characteristics (comparable) supporting text	Knowledge of the allocated interventions adequately prevented during the study	Knowledge of the allocated interventions adequately prevented during the study supporting text	Protection against contamination	Protection against contamination supporting text	Incomplete outcome data (attrition bias)	Incomplete outcome data (attrition bias) supporting text	Selective outcome reporting (reporting bias)	Selective outcome reporting (reporting bias) supporting text	Other risks of bias	Other risks of bias supporting text
														available for these 3 participants, and they were excluded from all analyses. A further 21 participants (n = 12 IJS; n = 9 SAU) were treated in residential rehabilitation facilities and did not have any level of face-to-face judicial supervision. These 21 participants were excluded from the analyses in this study. Intention-to-treat analyses in an interim				

Study ID	Random sequence generation (selection bias)	Random sequence generation (selection bias) supporting text	Allocation concealment (selection bias)	Allocation concealment (selection bias) supporting text	Baseline outcome measurements (similar)	Baseline outcome measurements (similar) supporting text	Baseline characteristics (comparable)	Baseline characteristics (comparable) supporting text	Knowledge of the allocated interventions adequately prevented during the study	Knowledge of the allocated interventions adequately prevented during the study supporting text	Protection against contamination	Protection against contamination supporting text	Incomplete outcome data (attrition bias)	Incomplete outcome data (attrition bias) supporting text	Selective outcome reporting (reporting bias)	Selective outcome reporting (reporting bias) supporting text	Other risks of bias	Other risks of bias supporting text
														report found that that the exclusion of these participants did not introduce any bias into the results (Jones, 2011)				
(MacDonald et al., 2007)	Low	Assignment envelopes were numbered and opened sequentially, allowing research staff to monitor judicial compliance with the random assignment procedure. No deviations from the random assignment protocol were detected	Low	Sealed random assignment envelopes	Low	No significant difference across groups	Low	Table of characteristics present	Unclear	Not reported.	Low	Separate procedures (Driving under influence court and mandatory minimum sanctions)	Low	The difference in attrition between the DUI court treatment assignment (n = 22; 15.8%) and the control condition (n = 26; 17.9%) was not statistically significant (t = 0.47; p = .63), suggesting no substantive pattern	Unclear		Unclear	See notes in paper

Study ID	Random sequence generation (selection bias)	Random sequence generation (selection bias) supporting text	Allocation concealment (selection bias)	Allocation concealment (selection bias) supporting text	Baseline outcome measurements (similar)	Baseline outcome measurements (similar) supporting text	Baseline characteristics (comparable)	Baseline characteristics (comparable) supporting text	Knowledge of the allocated interventions adequately prevented during the study	Knowledge of the allocated interventions adequately prevented during the study supporting text	Protection against contamination	Protection against contamination supporting text	Incomplete outcome data (attrition bias)	Incomplete outcome data (attrition bias) supporting text	Selective outcome reporting (reporting bias)	Selective outcome reporting (reporting bias) supporting text	Other risks of bias	Other risks of bias supporting text
														of attrition bias in our experimental design				
(NCT02978417, 2016)	Unclear	Not reported	Unclear	Not reported	Low	Similar characteristics	Low	Similar characteristics	Unclear	Not reported	Unclear	No details to prevent contamination reported	Low	Small numbers included in study and therefore only small numbers reported	Low	No evidence of this	Unclear	Limitations not discussed
(Rodriguez-Monguio et al., 2021)	High	Probationers were not randomly assigned to courts.	Low	unique identifiers used	Low	Propensity score matched groups...We also included probation offices that were similar to the drug courts in their sociodemographic composition (race/ethnicity and percentage of the population living in poverty) and population density at baseline but did not have a certified drug court (Gardner/Winchendon, Chicopee, and Woburn and Middlesex	Low	We controlled for these differences in our analyses	High	No blinding	Low	Different courts	Low	Missing data reported and levels are low.	Low		High	The BSAS dataset did not include dates of provision for treatment services so unclear how comparable the intervention was between groups

Study ID	Random sequence generation (selection bias)	Random sequence generation (selection bias) supporting text	Allocation concealment (selection bias)	Allocation concealment (selection bias) supporting text	Baseline outcome measurements (similar)	Baseline outcome measurements (similar) supporting text	Baseline characteristics (comparable)	Baseline characteristics (comparable) supporting text	Knowledge of the allocated interventions adequately prevented during the study	Knowledge of the allocated interventions adequately prevented during the study supporting text	Protection against contamination	Protection against contamination supporting text	Incomplete outcome data (attrition bias)	Incomplete outcome data (attrition bias) supporting text	Selective outcome reporting (reporting bias)	Selective outcome reporting (reporting bias) supporting text	Other risks of bias	Other risks of bias supporting text
						Superior Court). Per intention,Äêto,Äêtreat methodology, all matched probationers were included in the analysis. BSAS treatment services utilization and expenditures analyses controlled for relevant probationers,Äô de-mographic, substance use,Äêrelated, and recidivism,Äêrelated character-istics that remained statistically significant between probationers in drug and traditional courts after propensity score matching and probationer's length of enrollment in BSAS												

References

Deschenes, E. P., Turner, S., & Greenwood, P. W. (1995). Drug court or probation? An experimental evaluation of Maricopa County's drug court. *Justice System Journal 18*(1), 55-73.

Desland, M. L., & Batey, R. G. (1992). A 12-month prospective comparison of court-diverted with self-referred heroin users. *Drug Alcohol Rev*, *11*(2), 121-129. https://doi.org/10.1080/09595239200185591

Festinger, D. S., Dugosh, K. L., Kurth, A. E., & Metzger, D. S. (2016). Examining the efficacy of a computer facilitated HIV prevention tool in drug court. *Drug Alcohol Depend*, *162*, 44-50. https://doi.org/10.1016/j.drugalcdep.2016.02.026

Gottfredson, D. C., & Exum, M. L. (2002). The Baltimore City Drug Treatment Court: One year results from a randomized study. *Journal of Research in Crime and Delinquency 39*(3), 337-356.

Green, M., & Rempel, M. (2012). Beyond crime and drug use: Do adult drug courts produce other psychosocial benefits. *Journal of Drug Issues 42*(2), 156-177.

Harrell, A., Cavanagh, S., & Roman, J. (1998). *Findings from the evaluation of the D.C. Superior Court drug intervention program*.

Harrell, A., Roman, J., & Sack, E. (2001). *Drug court services for female offenders, 1996-1999: Evaluation of the Brooklyn Treatment Court*.

Jones, C. G. A. (2013). Early-phase outcomes from a randomized trial of intensive judicial supervision in an Australian drug court. *Criminal Justice and Behavior*, *40*(4), 453-468.

MacDonald, J. M., Morral, A. R., Raymond, B., & Eibner, C. (2007). The efficacy of the Rio Hondo DUI court: a 2-year field experiment. *Eval Rev*, *31*(1), 4-23. https://doi.org/10.1177/0193841X06287189

NCT02978417. (2016). *Feasibility study of extended-release Naltrexone (Vivitrol) in drug court settings*. https://clinicaltrials.gov/study/NCT02978417

Rodriguez-Monguio, R., Montgomery, B., Drawbridge, D., Packer, I., & Vincent, G. M. (2021). Substance use treatment services utilization and outcomes among probationers in drug courts compared to a matched cohort of probationers in traditional courts. *Am J Addict 30*, 505-513.
