# Supplementary material for: Effectiveness of legally mandated non-custodial drug and alcohol treatment orders for improved health, well-being, global functioning and quality of life: a systematic review and meta-analysis
Source: Health Justice. 2026 Jan 27;14:11. doi: 10.1186/s40352-025-00354-4 (PMC12958499; doi:10.1186/s40352-025-00354-4)

Additional file 13. Forest plots

13.1: Biological alcohol and/or drug use using objective measures

Number of positive drug screens at 12 months

	Drug Court			Control				Mean Difference
**Study or Subgroup**	**Mean**	**SD**	**Total**	**Mean**	**SD**	**Total**	**Weight**	**IV, Fixed, 95% CI**
NCT02978417	2	1.87	5	2.8	2.59	5	100.0%	−0.80 [−3.60, 2.00]
**Total (95% CI)**			**5**			**5**	**100.0%**	**−0.80 [−3.60, 2.00]**

Heterogeneity: Not applicable
Test for overall effect: Z = 0.56 (P = 0.58)

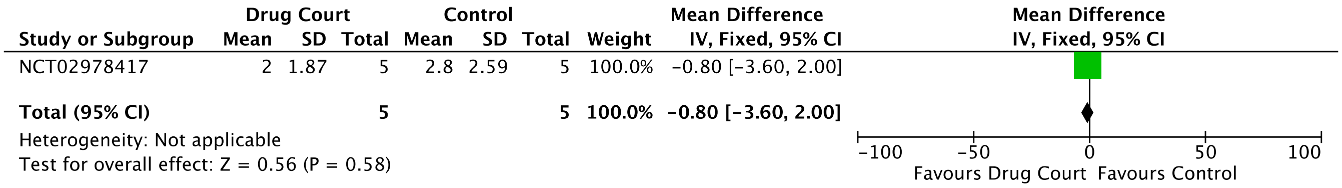

13.2: Depression outcome measures

Numbers of participants reported as depressed at 6 month and 18 months

	Drug Court		Control			Risk Ratio
**Study or Subgroup**	**Events**	**Total**	**Events**	**Total**	**Weight**	**M-H, Fixed, 95% CI**
**1.9.1 Numbers reported as depressed at 6 months**						
Green 2012	262	1009	147	524	100.0%	0.93 [0.78, 1.10]
**Subtotal (95% CI)**		**1009**		**524**	**100.0%**	**0.93 [0.78, 1.10]**
Total events	262		147			
Heterogeneity: Not applicable						
Test for overall effect: Z = 0.88 (P = 0.38)						
**1.9.2 Numbers reported as depressed at 18 months**						
Green 2012	257	951	152	523	100.0%	0.93 [0.78, 1.10]
**Subtotal (95% CI)**		**951**		**523**	**100.0%**	**0.93 [0.78, 1.10]**
Total events	257		152			
Heterogeneity: Not applicable						
Test for overall effect: Z = 0.84 (P = 0.40)						

Test for subgroup differences: Chi^2 = 0.00, df = 1 (P = 0.97), I^2 = 0%

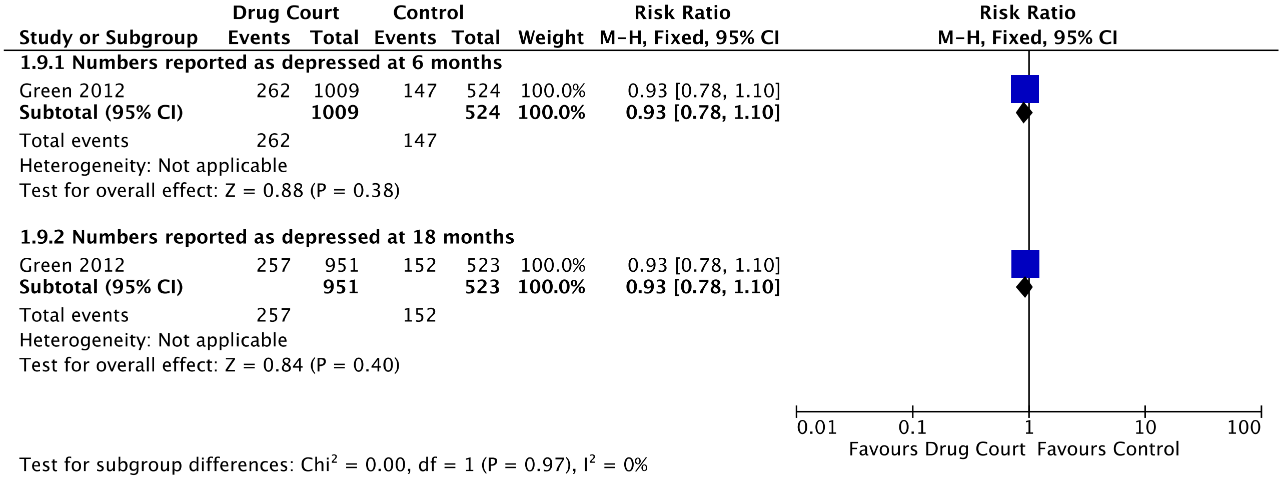

13.3: Adverse events

Serious adverse events

	Drug Court Intervention		Control			Risk Ratio
**Study or Subgroup**	**Events**	**Total**	**Events**	**Total**	**Weight**	**M-H, Fixed, 95% CI**
NCT02978417	0	5	1	5	100.0%	0.33 [0.02, 6.65]
**Total (95% CI)**		**5**		**5**	**100.0%**	**0.33 [0.02, 6.65]**
Total events	0		1			

Heterogeneity: Not applicable
Test for overall effect: Z = 0.72 (P = 0.47)

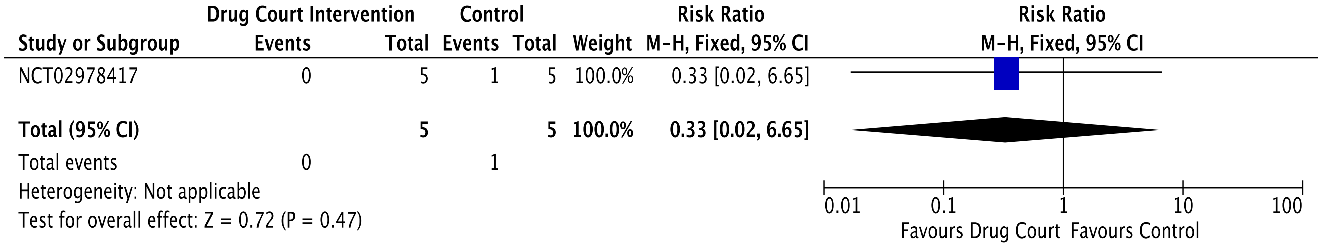

Supplement: Supplementary file 13 — Additional file 13. Forest plots. Graphical data related to the forest plots conducted meta-analyses conducted [file 40352_2025_354_MOESM13_ESM.pdf]
