# Supplementary material for: Effectiveness of legally mandated non-custodial drug and alcohol treatment orders for improved health, well-being, global functioning and quality of life: a systematic review and meta-analysis
Source: Health Justice. 2026 Jan 27;14:11. doi: 10.1186/s40352-025-00354-4 (PMC12958499; doi:10.1186/s40352-025-00354-4)
Supplement: Supplementary file 14 — Additional file 14. Reflexivity. Details of the authors’ backgrounds, expertise and assumptions relevant to the review [file 40352_2025_354_MOESM14_ESM.pdf]

# Additional file 14. Reflexivity

The core team consistently maintained a reflexive position throughout the review process. This was facilitated by weekly meetings which allowed the core team to review process by interrogating how professional and personal assumptions could influence interpretation of the data and interpretation of our own findings. We made clear any potential conflicts of interest, for example, regarding views and attitudes towards particular findings and conclusions.

The core team has varied professional and academic backgrounds including psychology, development of qualitative evidence synthesis methodology, information specialist/systematic review methodologist, Cochrane systematic reviews (all), health professions and health and social care services research (all).

Some of the core team have personal experience of substance use problems through affected family members; for confidentiality and anonymity we have chosen not to disclose which team members. Members of the core team believe that people with substance use problems have a fundamental right to compassionate treatment, others have no strong views on the use of mandatory treatment orders. Core team members have not published any eligible studies and so there is a low risk of biased appraisal when assessing study methodological limitations. The core team had no preconceptions of what the findings of our reviews might reveal. The review process and progress were regularly assessed and discussed between the review authors, topic experts and PPI and stakeholder contributors.

Our review topic experts have expertise in drug and alcohol research and on people with involved in the criminal justice system and have authored several study publications on these topics. Our PPI lead has expertise in public health. Engagement with our wider PPI and stakeholder contributor group throughout the review has contributed further topic expertise and minimised the risk of our preconceptions and backgrounds influencing our selection of qualitative studies for objective 2 and analysis and the interpretation of the findings.

## Equality, Diversity and Inclusion (EDI)

To improve the transparency of this review our team undertook to complete an Equality Diversity and Inclusion evaluation for systematic reviews prior to running the literature searches. (Centre for Ethnic Health Research, 2023) Copies of this documentation are available on request.

References

Centre for Ethnic Health Research. (2023). *Equality impact assessment (EqIA) form for systematic reviews*. NIHR Applied Research Collaboration East Midlands. Retrieved 1 September from https://arc-em.nihr.ac.uk/clahrcs-store/equality-impact-assessment-eqia-toolkit
